# Supplementary material for: Discovering covalent cyclic peptide inhibitors of peptidyl arginine deiminase 4 (PADI4) using mRNA-display with a genetically encoded electrophilic warhead
Source: Commun Chem. 2024 Dec 19;7:304. doi: 10.1038/s42004-024-01388-9 (PMC11659602; doi:10.1038/s42004-024-01388-9)
Supplement: Supplementary file 2 — Supplementary Information [file 42004_2024_1388_MOESM2_ESM.pdf]

Discovering Covalent Cyclic Peptide Inhibitors of PADI4 Using mRNA-display with a Genetically Encoded Electrophilic Warhead

Supplementary Information

Isabel R. Mathiesen,[1,2] Ewen D. D. Calder,[1,2] Simone Kunzelmann,[3] Louise J. Walport[1,2*]

[1] Protein-Protein Interaction Laboratory, The Francis Crick Institute, London NW1 1AT, United Kingdom

[2] Department of Chemistry, Molecular Sciences Research Hub, Imperial College London, London W12 0BZ, United Kingdom

[3]Structural Biology Scientific Technology Platform, The Francis Crick Institute, London NW1 1AT, United Kingdom

*To whom correspondence should be addressed: l.walport@imperial.ac.uk

Table of Contents

Supplementary Methods

S1. mRNA synthesis

S2. Aminoacylation of microhelix RNA and tRNA

S3. Test translation and MALDI-TOF mass spectrometry

S4. Bead-protein stability

S5. Clone assay

S6. Chemical Synthesis

S7. Peptide Synthesis

S8. Surface plasmon resonance (SPR)

S9. Intact Mass Spectrometry

S10. In-Fusion Cloning

S11. Mutagenesis

S12. Protein Expression

Supplementary Figures

Supplementary Figure S1. Translation with FAO optimisation

Supplementary Figure S2. Bead binding assessment for PADI4

Supplementary Figure S3. Intact mass spectrometry of PADI4 and PADI4_3_H4(**2**)

Supplementary Figure S4. Characterisation of PADI4 C645A

Supplementary Figure S5. Intact mass spec of PADI4 C645A and PADI4_3_H4(**2**)

Supplementary Figure S6. Test translation

Supplementary Figure S7. Multiple sequence alignment

Supplementary Figure S8. Intact mass spectrometry for selection 1 peptides

Supplementary Figure S9. Surface plasmon resonance for selection 1 peptides

Supplementary Figure S10. Characterisation of PADIs 1-3.

Supplementary Figure S11. COLDER assays with cP4_4/cP4_15 and PADIs 1-4.

Supplementary Figure S12. Surface plasmon resonance for peptide 4 variants

Supplementary Figure S13. Intact mass spectroscopy with Cl4

Supplementary Figure S14. Intact mass spectrometry for selection 2 peptides

Supplementary Figure S15. COLDER assays for selection 2 peptides

Supplementary Figure S16. Surface plasmon resonance for selection 2 peptides

Supplementary Figure S17. Biotinylation of PADI4 C645A

Supplementary Figure S18. Number of sequences for C645A selection

Supplementary Figure S19. Analytical HPLC traces for PADI4 peptides and variants

Supplementary Figure S20-25. Full size SDS-PAGE gels

Supplementary Tables

Supplementary Table S1. List of oligonucleotides

Supplementary Table S2. List of peptides synthesised

Supplementary Table S3. HPLC gradients used for the purification of all peptides

Supplementary Table S4. Covalent peptide SPR data

Supplementary References

Supplementary Methods

S1. mRNA synthesis

mRNA Templates:

mRNA templates PADI4_3, PADI4_3_R2M, PADI4_3_H4M and HiBiT_EloAsn were made by two rounds of overlapping PCR. The first of which was 7 cycles at 100 µL scale (1X KOD polymerase buffer, 1 mM $MgCl_2$, 0.2 mM dNTPs, 0.6 µM T7g10M.F46 primer, 0.5 µM template primer, 0.8 µL KOD polymerase), the annealing temperature was 55 °C. The second PCR round was 6 cycles on a 1 mL scale, using 100 µL of the first-round product to extend from (1X KOD polymerase buffer, 1 mM $MgCl_2$, 0.2 mM dNTPs, 0.25 µM T7g10M.F46 primer, 0.25 µM CGS3an13.R39 primer, 1.6 µL KOD polymerase), the annealing temperature was 61 °C. See Supplementary Table S1 for primer information.

The PCR product was purified by phenol-chloroform extraction and ethanol precipitation. Overnight transcription of the PCR product was performed on a 1000 µL scale at 37 °C, with 600 units T7 RNA polymerase (Thermo Scientific), 1X T7 buffer, 10 mM DTT, 20 mM $MgCl_2$, 3.75 mM NTPs. Then 1x DNase buffer and 30 units DNase (Thermo Scientific) were added for 1 hour. The RNA was precipitated with isopropanol and purified by separation on and extraction from a 4 M urea denaturing 8% PAGE gel (19:1 acrylamide/bis-acrylamide).

mRNA Libraries:

To make the mRNA libraries (NNK6-NNK10) the above protocol was followed but with 0.5 µM stdlib_NNK6-10 in the first PCR mix, rather than the template primer.

S2. Aminoacylation of microhelix RNA and tRNA

With ClAc-D-Tyr-CME:

5 mM ClAc-D-Tyr-CME was mixed with 600 mM $MgCl_2$, 20% DMSO, 25 µM eFx, 25 µM initiator $tRNA^{fMet}_{CAU}$ and 50 mM HEPES-KOH for translations and selections.

With N-δ-fluoroacetimidoyl ornithine-CBT:

4 mM FAO-CBT was mixed with 600 mM $MgCl_2$, 20% DMSO, 25 µM eFx and either 25 µM microhelix (FAM-MiHx_23b, Integrated DNA Technologies) for determining aminoacylation efficiency or 25 µM elongator $tRNA^{AsnE2}_{CAU}$ (T-stems #1-4 were tested) and 50 mM HEPES-KOH for translations.

After 2 hours on ice, the resulting aminoacyl-microhelix/tRNA was purified by ethanol precipitation. Pellets were washed twice with 70% ethanol containing 0.1 M sodium acetate (pH 5.2). For the microhelix, they were analysed on a 20% polyacrylamide gel containing 50 mM sodium acetate (pH 5.2) by detection of the FAM label on a Typhoon FLA 9500 (GE Healthcare) and quantified with Fiji. Flexizyme eFx, initiator tRNA and elongator tRNAs #1-4 were synthesised according to the previously described protocols.[1,2]

S3. Test translation and MALDI-TOF mass spectrometry

The PURExpress™ Δ (aa, tRNA) *in vitro* protein synthesis kit (NEB) was used to translate mRNA templates HiBiT_EloAsn, PADI4_3, PADI4_3_R2M, PADI4_3_H4M. A homemade solution A was used (100 mM potassium acetate, 50 mM HEPES-KOH (pH 7.6), 20 mM creatine phosphate, 6 mM magnesium acetate, 2 mM spermidine, 2 mM ATP, 2 mM GTP, 1 mM CTP, 1 mM UTP (all NTPs from Jena Bioscience), 1.5 mg/mL *E. coli* tRNA (Roche) and 14 µM DTT). Translation reactions consisted of 0.78 µL solution A, 1.5 µL solution B, 0.5 µL each aminoacyl-tRNA (ClAc-D-Tyr-$tRNA^{fMet}_{CAU}$ and FAO-$tRNA^{AsnE2\#1-4}_{CAU}$) in 1 mM NaOAc, 1 µL mRNA template (5 µM), 0.5 µL amino acid mix (5 µM each, 19aa -Met) and 0.22 µL water. After 1 hour incubation at 37 °C, 1 µL EDTA (pH 8, 100 mM) was added to promote cyclisation and the mixture incubated for a further 30 min at 37 °C. The mixture was desalted and concentrated with $ZipTip_{u\text{-}c18}$ (Millipore), co-crystallised with α-cyano-4-hydroxycinnamic acid and analysed using a MALDI-TOF (Shimadzu MALDI-8030) in positive linear mode.

The HiBiT assay was performed in a 96-well white plate (Greiner). The translation products from the HiBiT_EloAsn template, translated with or without each of the FAO-$tRNA^{AsnE2\#1-4}_{CAU}$ T-stem variants, were diluted 500-fold in PBS-T (PBS, 0.1 % Tween20). 1 µL of each diluted translation product was added to 99 µL detection solution containing furimazine substrate

(1:100, Promega) and LgBiT protein (1:200, Promega) in PBS-T. A standard curve for HiBiT quantification was also prepared in the same way but with dilution series of HiBiT-HaloTag control protein (Promega) in place of the translation product. The plate was incubated at rt for 10 min with gentle rocking and read using a CLARIOstar PLUS (BMG LABTECH) with a 470 nm (80 nm bandpass) filter.

S4. Bead-protein stability

Magnetic streptavidin beads (Life Technologies) were washed with 3 x 20 µL PADI4 selection buffer (50 mM HEPES, 150 mM NaCl, 10 mM $CaCl_2$, 2 mM DTT, 0.1% Tween, pH 7.5). 1 µM biotinylated PADI4 was added for 15 min on ice. 25 µM biotin was added for 15 mins on ice, prior to washes with PADI4 selection buffer. Streptavidin bead-immobilised PADI4 was incubated on ice with 0, 3, 5 or 8 M guanidine HCl (2x 20 min). After washes with PADI4 selection buffer (3 x 1 min) the beads were heated at 95 °C for 5 min. The samples were separated by SDS-PAGE on a 4–20% TruPAGE™ Precast gel (Sigma-Aldrich) and visualised by Quick Coomassie staining (Neo Biotech).

S5. Clone assay

The PURExpress™ Δ (aa, tRNA) *in vitro* protein synthesis kit (NEB) was used to translate mRNA templates HiBiT_EloAsn, PADI4_3, PADI4_3 R2M, PADI4_3_H4M. A homemade solution A was used (100 mM potassium acetate, 50 mM HEPES-KOH (pH 7.6), 20 mM creatine phosphate, 6 mM magnesium acetate, 2 mM spermidine, 2 mM ATP, 2 mM GTP, 1 mM CTP, 1 mM UTP (all NTPs from Jena Bioscience), 1.5 mg/mL *E. coli* tRNA (Roche) and 14 µM DTT). Translation mixes were made from 0.78 µL solution A, 1.5 µL solution B, 0.5 µL each aminoacyl-tRNA (ClAc-D-Tyr-tRNA$^{fMet}_{CAU}$ and FAO-tRNA$^{AsnE2\#4}_{CAU}$) in 1 mM NaOAc, 1 µL mRNA template (5 µM), 0.5 µL amino acid mix (19aa -Met) and 0.22 µL water. After 1 hour incubation at 37 °C, 1 µL EDTA (pH 8, 100 mM) was added to promote cyclisation and the mixture incubated for a further 30 min at 37 °C. The mixture was reverse transcribed with M-MLV RTase, RNase H minus and then buffer exchanged with a 1 mL sephadex column into PADI4 selection buffer (50 mM HEPES, 150 mM NaCl, 10 mM $CaCl_2$, 2 mM DTT, 0.1% Tween, pH 7.5). An equal volume of blocking solution was added (PADI4 selection buffer with 0.2% (w/v) acetyl-BSA and 2 mg/mL salmon sperm DNA). The mixture was incubated with magnetic

streptavidin bead (Life Technologies)-immobilised PADI4 (200 nM, 30 min) and then subject to 5 M guanidine HCl (2 x 20 min) washes at 0 °C, apart from the positive control. Additional washes with assay buffer (3 x 1 min) were performed. The cDNA was recovered from the beads by heating to 95 °C for 5 min. DNA recovery was measured by quantitative real-time PCR, using primers T7g10M.F46 and CGS3an13.R22, and calculated as a percentage of the input DNA.

S6. Chemical Synthesis

All NMR spectra are available in Supplementary Data 4.

Ethyl 2-fluoroethanimidate hydrochloride (S1)[3]:

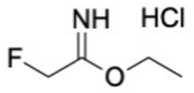

2-Fluoroacetonitrile (500 mg, 8.30 mmol, 531 µL, 1 equiv) and EtOH (430 mg, 9.33 mmol, 374 µL, 1.1 equiv) were added to 2 M HCl in Et_2O (20.0 mmol, 10.0 mL, 4.72 equiv) under argon. The reaction was left overnight at rt with continual stirring. The solids were collected by filtration and dried under reduced pressure. This yielded ethyl 2-fluoroethanimidate hydrochloride (**S1**) (452 mg, 3.19 mmol, 38%). ^{1}H NMR (400 MHz, CD_3OD) δ 5.36 (d, $^2J_{H\text{-}F}$ 44.9 Hz, 2H, CH_2F), 3.61 (q, J 7.0 Hz, 2H, CH_2), 1.18 (t, J 7.0 Hz, 3H, CH_3). ^{19}F NMR (376 MHz, CD_3OD) δ –234.49; LC/MS could not be observed under positive or negative mode.

2,2,2-Trichloroethyl acetimidate hydrochloride (S2)[4]:

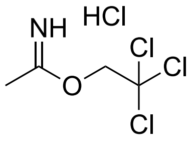

Acetonitrile (416 mg, 7.97 mmol, 327 µL, 1.2 equiv) and trichloroethanol (1.00 g, 6.69 mmol, 1.55 mL, 1 equiv) were stirred on ice whilst HCl gas (generated from $CaCl_2$ and conc. HCl as per Arnáiz, 1995[5]) was bubbled through the solution for 5 h. The solution was refrigerated for 66 h and the crystals were washed with acetonitrile to yield 2,2,2-trichloroethyl acetimidate

hydrochloride (**S2**) (666 mg, 2.94 mmol, 44%). ^{1}H NMR (400 MHz, DMSO-d_6) δ 12.66 (s, 1H), 5.35 (s, 2H), 2.55 (s, 3H). Spectroscopic data as previously reported.[4]

Ethyl 2-chloroethanimidate hydrochloride (S3)[6]:

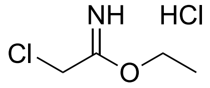

2-Chloroacetonitrile (627 mg, 8.30 mmol, 748 µL, 1 equiv) and EtOH (430 mg, 9.33 mmol, 374 µL, 1.1 equiv) were added to 2 M HCl in Et_2O (20.0 mmol, 10.0 mL, 4.72 equiv) under argon. The reaction was left for 1 h at rt with continual stirring. The solids were collected by filtration and dried under reduced pressure. This yielded ethyl 2-chloroethanimidate hydrochloride (**S3**) (636 mg, 4.02 mmol, 48%). ^{1}H NMR (400 MHz, CD_3OD) δ 4.60 (s, 2H, CH_2Cl), 3.61 (q, *J* 7.0 Hz, 2H, CH_2), 1.18 (t, *J* 7.0 Hz, 3H, CH_3).

***N*-α-Boc-*N*-δ-(2-fluoroacetimidoyl)-ornithine (S4):**

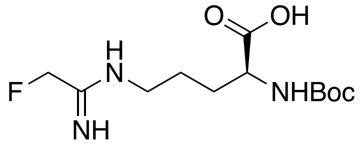

N-α-Boc-ornithine (232 mg, 1.00 mmol, 1.0 eq) was suspended in a mixture of absolute ethanol (3.3 mL) and *N,N*-diisopropylethylamine (0.523 mL, 3.00 mmol, 3.0 eq). Ethyl 2-fluoroethanimidate hydrochloride (**S3**) (210 mg, 2.00 mmol, 2.0 eq) was added and the suspension was stirred at rt for 2 h then concentrated *in vacuo*. Purification by reverse phase column chromatography (0 to 30%, water:acetonitrile (+0.1% formic acid)) then concentration by lyophilisation yielded the title compound (**S4**) (263 mg, 90%) as an off white solid. ^{1}H NMR (400 MHz, CD_3OD) δ_H 5.28 (2H, d, $^{2}J_{H-F}$ 45.4), 4.05 – 3.97 (1H, m), 3.46 – 3.34 (2H, m), 1.91 – 1.79 (1H, m), 1.78 – 1.67 (3H, m), 1.46 (9H, s); ^{13}C NMR (101 MHz, DMSO-d_6) δ_C 178.6, 164.1 (d, $^{2}J_{C-F}$ 19.6), 157.8, 80.2, 79.0 (d, $^{1}J_{C-F}$ 178.7), 56.0, 43.1, 31.5, 28.8, 24.8; ^{19}F NMR (376 MHz, $CDCl_3$) δ_F –229.5.; LC/MS Rt 0.86 min, >99%, m/z (ESI$^+$) 292 (100%, $[M+H]^+$), (ESI$^-$) 290 (100%, $[M–H]^-$).

***N*-δ-(2-Fluoroacetimidoyl)-ornithine chlorobenzylthioester (FAO-CBT) (3):**

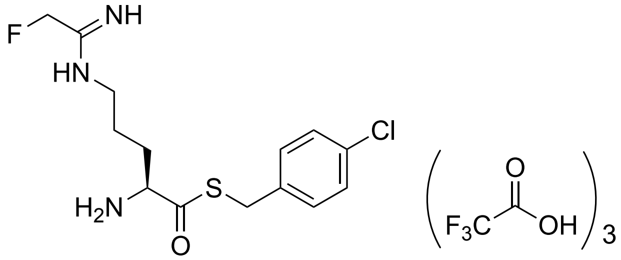

N-α-Boc-*N*-δ-(2-fluoroacetimidoyl)-ornithine (**S4**) (0.10 g, 0.34 mmol, 1.0 eq) was dissolved in *N,N*-dimethylformamide (5.0 mL). Triethylamine (0.14 mL, 1.0 mmol, 3.0 eq) was added to the solution followed by PyBOP (0.21 mg, 0.41 mmol, 1.2 eq) and it was stirred for 15 min at room temperature (rt). 4-Chlorobenzenemethanethiol (0.091 mL, 0.69 mmol, 2.0 eq) was added to the solution and it was stirred for 18 h at rt then concentrated *in vacuo* and partially purified by RP column chromatography (0 to 100%, water:acetonitrile (+0.1% formic acid)). Fractions containing the desired product were concentrated by lyophilisation. The resulting solid was dissolved in water (7.0 mL) and cooled to 0 °C, trifluoroacetic acid (7.0 mL) was added and the solution was stirred while warming to rt over 2 h. Purification by RP preparative HPLC (0 to 50% water:acetonitrile (+0.1% formic acid)) and concentration of the fractions containing the desired product (**3**) by lyophilisation yielded the title compound (9.7 mg, 4%) as a colourless, highly hygroscopic solid. ^{1}H NMR (400 MHz, DMSO-d_6) δ_H 9.45 (1H, s), 9.23 (1H, br.s), 8.62 (2H, br.s), 7.44 – 7.33 (4H, m), 5.28 (2H, d, $^{2}J_{H\text{-}F}$ 45.3), 4.32 (1H, t, *J* 6.3), 4.26 (2H, s), 3.30 (2H, t, *J* 6.9), 1.94 – 1.74 (2H, m), 1.72 – 1.51 (2H, m); ^{13}C NMR (101 MHz, DMSO-d_6) δ_C 196.1, 162.0 (d, $^{2}J_{C\text{-}F}$ 19.3), 158.2 (q, $^{2}J_{C\text{-}F}$ 31.6), 136.1, 132.1, 130.7, 128.5, 117.0 (q, $^{1}J_{C\text{-}F}$ 299.1), 78.1 (d, $^{1}J_{C\text{-}F}$ 175.6), 57.8, 40.9, 31.7, 28.3, 22.7; ^{19}F NMR (376 MHz, $CDCl_3$) δ_F –73.7 (1F), –229.1 (9F); LC/MS Rt 0.85 min, 91%, m/z (ESI$^+$) 332 (100%, $[M+H]^+$), (ESI$^-$) not observed in negative mode.

S7. Peptide Synthesis

Solid-phase peptide synthesis was performed using Fmoc-protected amino acids (5.0 equiv) and low loading Rink Amide resin (172.4 mg, 0.29 mmole/g, Novabiochem) on a 0.05 mM scale, with a Gyros Protein Technologies PreludeX automated synthesizer (Gyros Protein Technologies AB, Sweden). Amino acid couplings occurred in DMF using DIC (5 equiv) in DMF and Oxyma (5 equiv) in DMF. Capping was performed after each coupling with acetic

anhydride (20 equiv) in DMF and DIPEA (20 equiv) in DMF. Fmoc groups were removed with 20% piperidine in DMF. Fmoc-Orn-DDE was used to prepared FAO warhead-containing peptides, Me4 and Cl4; Fmoc-Cit-OH (Fluorochem) was used to prepare Cit4 (Table S2).

FAO warhead-containing peptides:

The peptide-loaded resins were incubated with *N*-chloroacetoxysuccinimide (47.9 mg, 0.30 mmol, 6.0 eq) in DMF for 1 h and then MMT deprotection of Cys was performed with TFA (1%), TIPS (5%) in DCM (8 x 2 min). Cyclisation was promoted by incubation with DIPEA for 16 h (2% in DMF). The resins were then incubated in 3 x 5 mL of hydrazine (2% in DMF) for 5 min to remove the DDE protecting group. Ethyl 2-fluoroethanimidate (**S1**) (28.3 mg, 0.20 mmol, 4.0 eq) and triethylamine (22.9 mg, 31.5 µL, 0.23 mmol, 4.5 eq) were dissolved in EtOH (1.5 mL) and DMF (2.5 mL) and added to the resins for 2 h.[7] Bulk cleavage and deprotection was performed with a mixture of TFA (95%), H_2O (2.5%) and TIPS (2.5%) at rt for 2 h. The peptides were precipitated with cold diethyl ether and dissolved in DMSO.

Warhead-free peptides Arg4 and Cit4:

The peptide-loaded resins were incubated with *N*-chloroacetoxysuccinimide (47.9 mg, 0.30 mmol, 6.0 eq) in DMF for 1 h. Bulk cleavage and deprotection was performed with a mixture of TFA (95%), H_2O (2.5%) and TIPS (2.5%) at rt for 2 h. The peptides were precipitated with diethyl ether, dissolved in DMSO and pH adjusted to 8 with TEA to promote cyclisation.

Peptide variants Me4 and Cl4:

The peptide-loaded resins were incubated with *N*-chloroacetoxysuccinimide (47.9 mg, 0.30 mmol, 6.0 eq) in DMF for 1 h and then MMT deprotection of Cys was performed with TFA (1%), TIPS (5%) in DCM (8 x 2 min). Cyclisation was promoted by incubation with DIPEA (2% in DMF). The resins were then incubated in 3 x 5 mL of hydrazine (2% in DMF) for 5 min to remove DDE.

Me4: 2,2,2-Trichloroethyl acetimidate hydrochloride (**S2**) (43.0 mg, 0.20 mmol, 4.0 eq) and triethylamine (22.9 mg, 31.5 µL, 0.23 mmol, 4.5 eq) were dissolved in EtOH (1.5 mL) and DMF (2.5 mL) was added to the resins for 2 h.[7]

Cl4: Ethyl 2-chloroethanimidate (**S3**) (31.6 mg, 0.20 mmol, 4.0 eq) and triethylamine (22.9 mg, 31.5 µL, 0.23 mmol, 4.5 eq) were dissolved in EtOH (1.5 mL) and DMF (2.5 mL) was added to the resins for 2 h.[7]

Bulk cleavage and deprotection was performed with a mixture of TFA (95%), H_2O (2.5%) and TIPS (2.5%) at rt for 2 h. The peptides were precipitated with diethyl ether and dissolved in DMSO.

All peptides were purified by reverse phase HPLC with a preparative C18 column (XBridge prep BEH C18, 19x250 mm, 10 µm). A flow rate of 24 mL/min was used with an optimised linear gradient over 32 or 47 min [solvent A: 99.9% (v/v) water, 0.1% (v/v) trifluoroacetic acid; solvent B: 99.9% (v/v) acetonitrile and 0.1% (v/v) trifluoroacetic acid] (see Table S2-S3). The purified cyclic peptides were analysed by reverse phase HPLC with an analytical C18 column (XBridge prep BEH C18, 4.6x250 mm, 5 µm) (see Fig. S20).

S8. Surface plasmon resonance (SPR)

Using a Biacore S200 (GE Healthcare), biotinylated His-PADI4 (80 nM) was immobilised on a CAP chip (GE Healthcare) to a response unit of approx. 3000. At 25 °C, single cycle kinetics mode was used with 5 samples of a 3-fold dilution series of peptides PADI4_3_H4(**2**), cP4_2, cP4_4, cP4_7, cP4_10, cP4_13, cP4_15, cP4_18 and cP4_165. Running buffer contained 50 mM HEPES, 150 mM NaCl, 10 mM $CaCl_2$, 0.05% Tween-20, 0.1% DMSO (pH 7.5). After each cycle, regeneration of the chip was carried out as per the manufacturer's protocol. Data were analysed with the Biacore S200 Evaluation Software, each in triplicate. Warhead-containing peptides were analysed using the two-state reaction model, with the rate constant for the reverse second step (k_{-2}) set to zero. The equilibrium constant of the reversible binding step, K_i, was calculated as the ratio of k_{-1}/k_{+1} and the rate constant of the irreversible chemical step, k_{inact}, as k_{+2}. The Arg4, Cit4 and Me4 peptides were fitted using a 1:1 binding model.

S9. Intact Mass Spectrometry

Samples were prepared with 20 µM PADI4 and 200 µM peptide ligand in 50 mM HEPES, 150 mM NaCl, 10 mM $CaCl_2$, 2 mM DTT (pH 7.5) and incubated for 1 hour at rt. Denatured proteins were injected onto a C4 BEH 1.7 µm, 1.0 x 100 mm UPLC column using an Acquity I class LC (Waters, UK). Proteins were eluted with a 15-minute gradient of acetonitrile (2% v/v to 80% v/v) in 0.1% v/v formic acid using a flow rate of 50 µl min^{-1}. The analytical column outlet was directly interfaced via an electrospray ionisation source, with a time-of-flight (TOF) mass

spectrometer (BioAccord, Waters, UK). Data was acquired over a m/z range of 300 –8000, in positive ion mode with a cone voltage of 40 V. Scans were summed together manually and deconvoluted using MaxEnt1 (Masslynx, Waters, UK).

S10. In-Fusion Cloning

The coding sequences of human *PADI1* (residue 1-663), *PADI3* (residue 1-664) and *PADI4* (residues 1-663) were amplified and each cloned into PCR linearised pQE80L vector. After resolving the PCR fragments and vector by gel electrophoresis on a 1% agarose gel, they were purified using the PureLink™ Quick Gel Extraction Kit (Invitrogen) following the manufacturer's protocol. Three mixtures were made with 50 ng of vector, 50 ng PADI1/3/4 fragment and 2 µL 5X In-Fusion HD Enzyme Premix (Takara Bio). The 10 µL mixtures were incubated for 15 min at 50 ^{o}C and 5 µL transformed into 50 µL *E. Coli* NEB® 5-alpha. A single LB agar plate colony was picked for each construct into 4 mL of LB media with ampicillin, incubated at 37 ^{o}C overnight and miniprepped (QIAprep Spin Miniprep Kit, Qiagen). The plasmid sequences were confirmed by Sanger sequencing (GeneWiz) or plasmid sequencing (FullCircle).

S11. Mutagenesis

Mutagenesis of pQE80L_hPADI4 was performed by PCR with Mut_C645A.F33 and Mut_C645A.R33 (Table S1), using PfuTurbo Polymerase (Agilent). 1 µL Dpn1 restriction enzyme (NEB) was added to the 50 µL PCR mixture and the sample incubated at 37 ^{o}C for 1 h. The mixture was transformed into *E. Coli* NEB® 5-alpha (High Efficiency, NEB) and plated on agar plates containing ampicillin (50 µg/mL). A single colony was picked into 4 mL of LB media (Crick Media Preparation) with ampicillin, incubated at 37 ^{o}C overnight and miniprepped (QIAprep Spin Miniprep Kit, Qiagen). Mutation C645A was confirmed by plasmid sequencing (FullCircle).

S12. Protein Expression

N-terminally biotinylated PADI2 and PADI4 with a His6 tag was expressed following previously described protocols using the p28BIOH-LIC vectors.[8] PADI4 with a His6 tag was also expressed, without biotinylation, following previously described protocols, for use in intact MS.[8]

pQE80L_hPADI1, pQE80L_hPADI3 and pQE80L-hPADI4_C645A were transformed into NiCo21(DE3) *E. coli* cells (NEB). A single colony for each was picked into LB with ampicillin and incubated at 37 ^{o}C overnight. Terrific broth was inoculated with the starter culture (1:50). At OD_{600} = 1 the cells were induced with 0.2 mM IPTG and 20 µM D-biotin (Fluorochem Limited) and the temperature dropped to 18 °C. After 16 hours, cells were harvested by centrifugation and resuspended in binding buffer (50 mM HEPES, pH 7.5, 500 mM NaCl, 5 mM imidazole, 5% glycerol supplemented with DNase1 and 1 x EDTA-free protease inhibitors (Roche)). Cells were lysed using sonication and the lysate was purified using an AKTA Pure (Cytiva) with a Ni-NTA 5 mL column. After washes (15 mM imidazole), the PADI4 C645A was eluted (200 mM imidazole). Fractions containing bio-His-PADI1/bio-His-PADI3/bio-His-PADI4_C645A were concentrated and further purified using an S200 size exclusion column (HiLoad 16/600 Superdex 200 (Cytiva)) in 50 mM HEPES, pH 7.5, 150 mM NaCl, 2 mM DTT, 5% glycerol. Pure fractions of protein were aliquoted, flash frozen and stored at -80 °C until use. Biotinylation of PADI4_C645A was confirmed through streptavidin bead binding (Figure S17).

Supplementary Figures

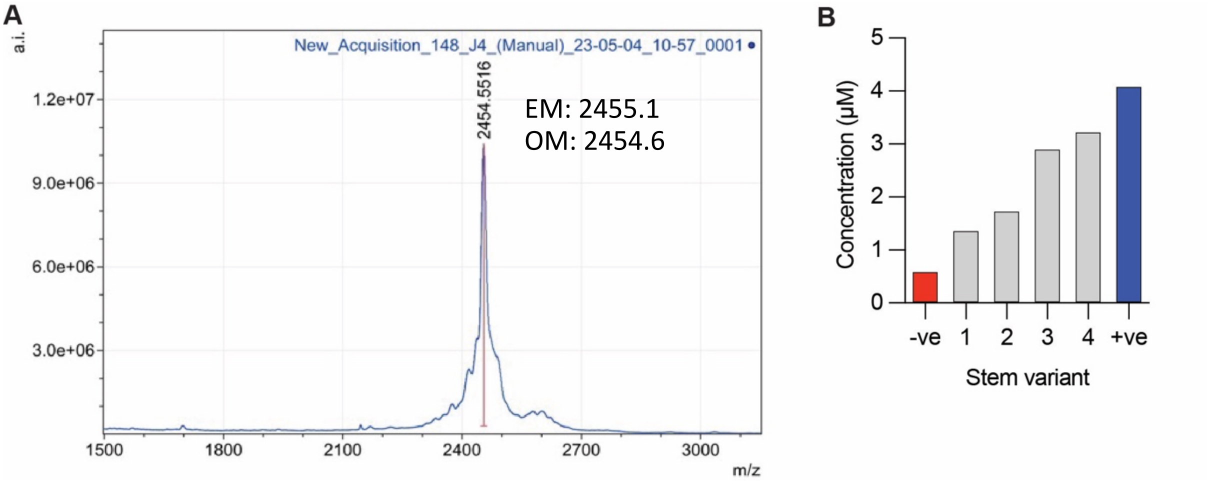

Figure S1. Translation with FAO optimisation. **A** Example MALDI-TOF spectra from the translation of the HiBiT_EloAsn template showing successful incorporation of FAO using tRNA T-stem variant #4.[2] The expected mass (EM) of the peptide was 2455.1 Da while the observed mass (OM) was 2454.6 Da. **B** The HiBiT_EloAsn mRNA template was translated using FAO-$tRNA^{AsnE2\#1-4}_{CAU}$ T-stem variants. Negative and positive controls were lacking any aminoacylated elongator tRNA, but the positive had methionine added back in. Quantification of translation efficiency with the HiBiT assay and standard curve showed differential translational efficiency of FAO into the HiBiT_EloAsn peptide depending on the T-stem variant.[2]

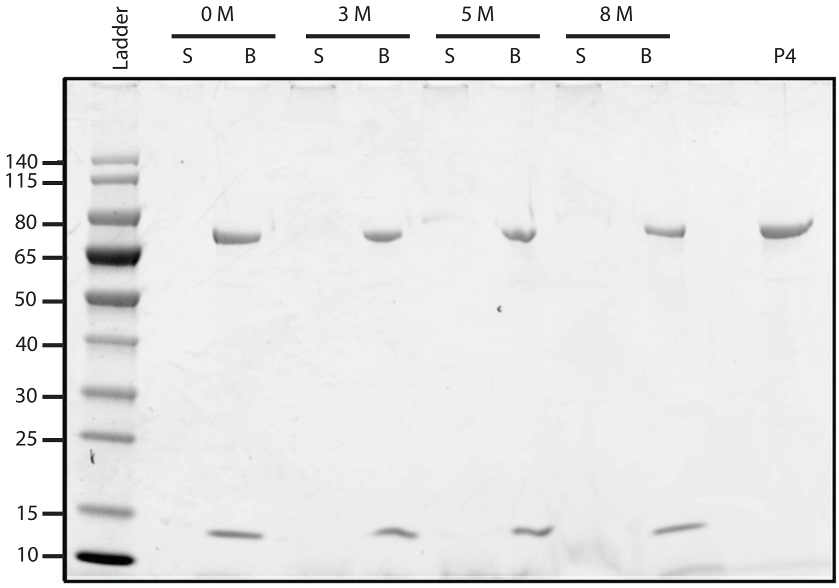

Figure S2. Bead binding assessment for PADI4. Varied concentrations of guanidinium HCl (3–8 M) do not cause significant disruption of biotinylated PADI4-streptavidin interactions. S – supernatant, B – beads, P4 – PADI4 alone.

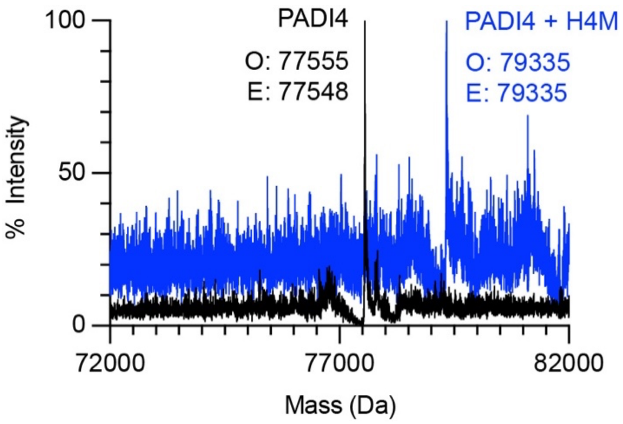

Figure S3. Intact mass spectrum of PADI4 with (blue) and without (grey) incubation with PADI4_3_H4(**2**) showing single labelling. E – expected molecular weight, O – observed molecular weight.

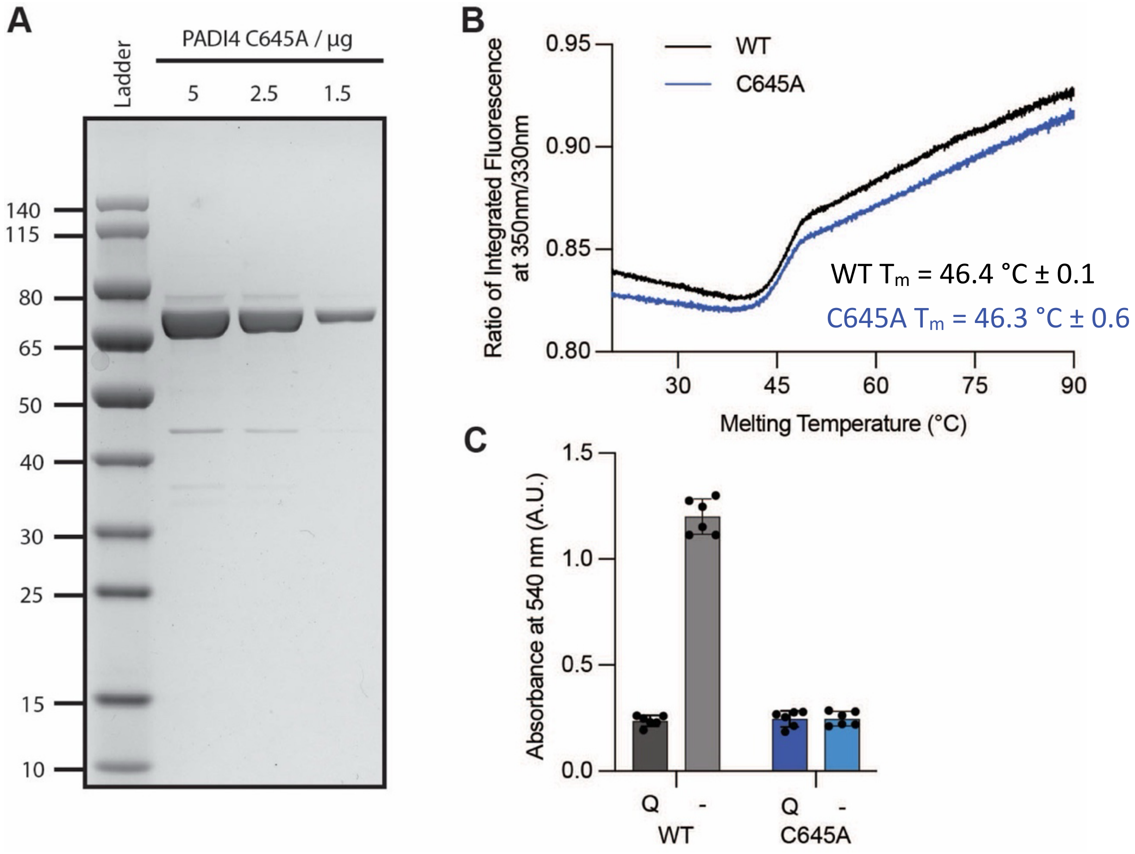

Figure S4. Characterisation of PADI4 C645A. **A** SDS PAGE gel with different concentrations of PADI4 C645A shows high level of purity. **B** NanoDST shows there is no significant difference between the melting points (T_m) for the wild type PADI4 (WT) and variant PADI4 C645A. **C** COLDER assay shows that the mutant is catalytically inactive, while the WT PADI4 is active unless it is quenched with 50 mM EDTA (Q). Individual data points are shown from two repeats each with three replicates and bars represent the mean ± 1 s.d..

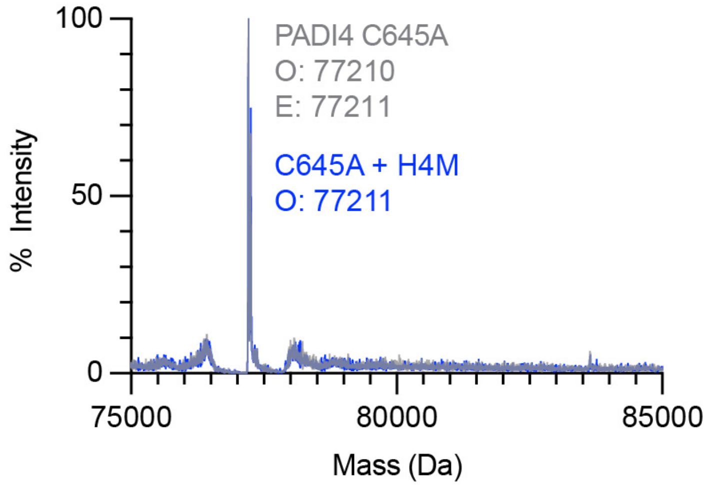

Figure S5. Intact mass spectrum of PADI4 C645A with (blue) and without (grey) incubation with PADI4_3_H4(2) showing no labelling.

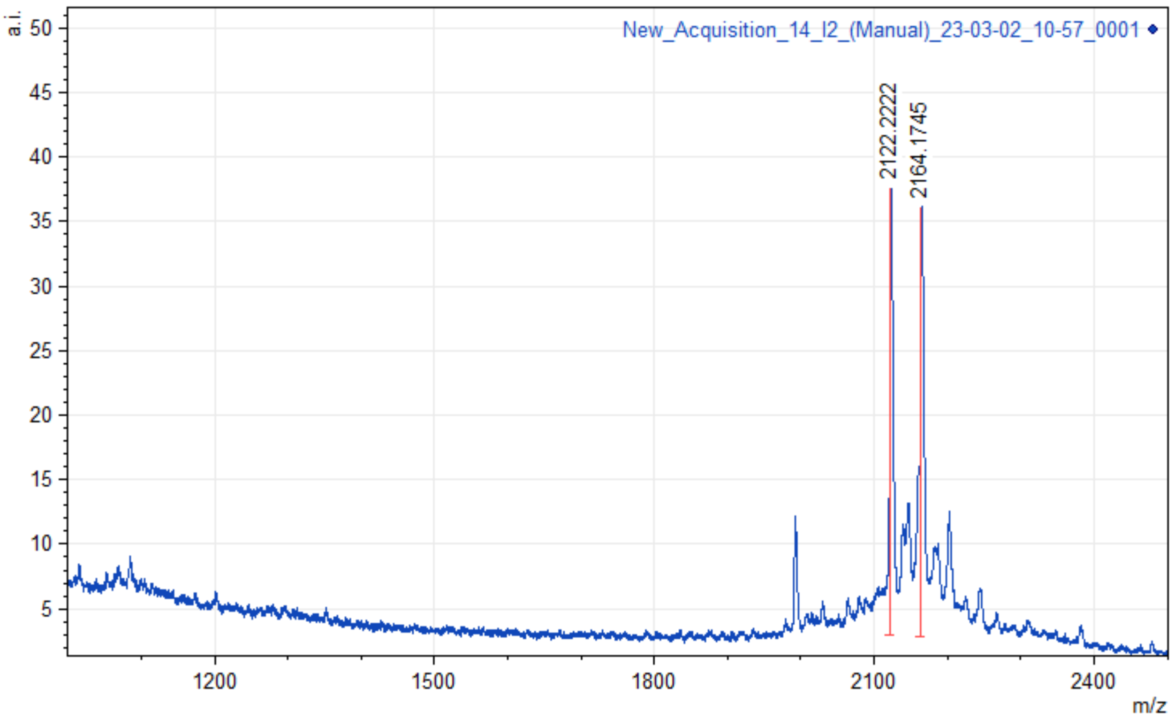

Figure S6. Test translation. Example MALDI-TOF spectra of PADI4_3_R2M showing successful incorporation of FAO where 2164.17 is the non-cyclised mass and 2122.22 is the cyclised mass.

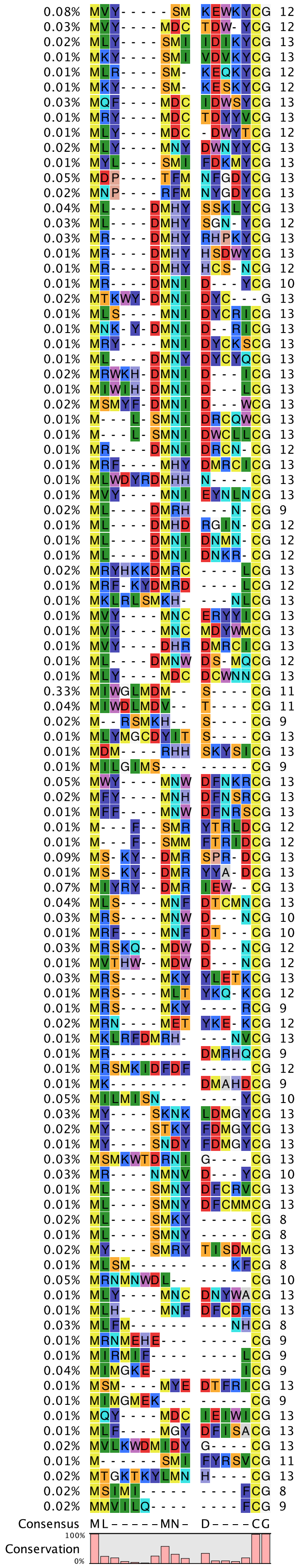

Figure S7. Multiple sequence alignment. Round 5 multiple sequence alignment of the top 100 most enriched sequences containing M≥2.

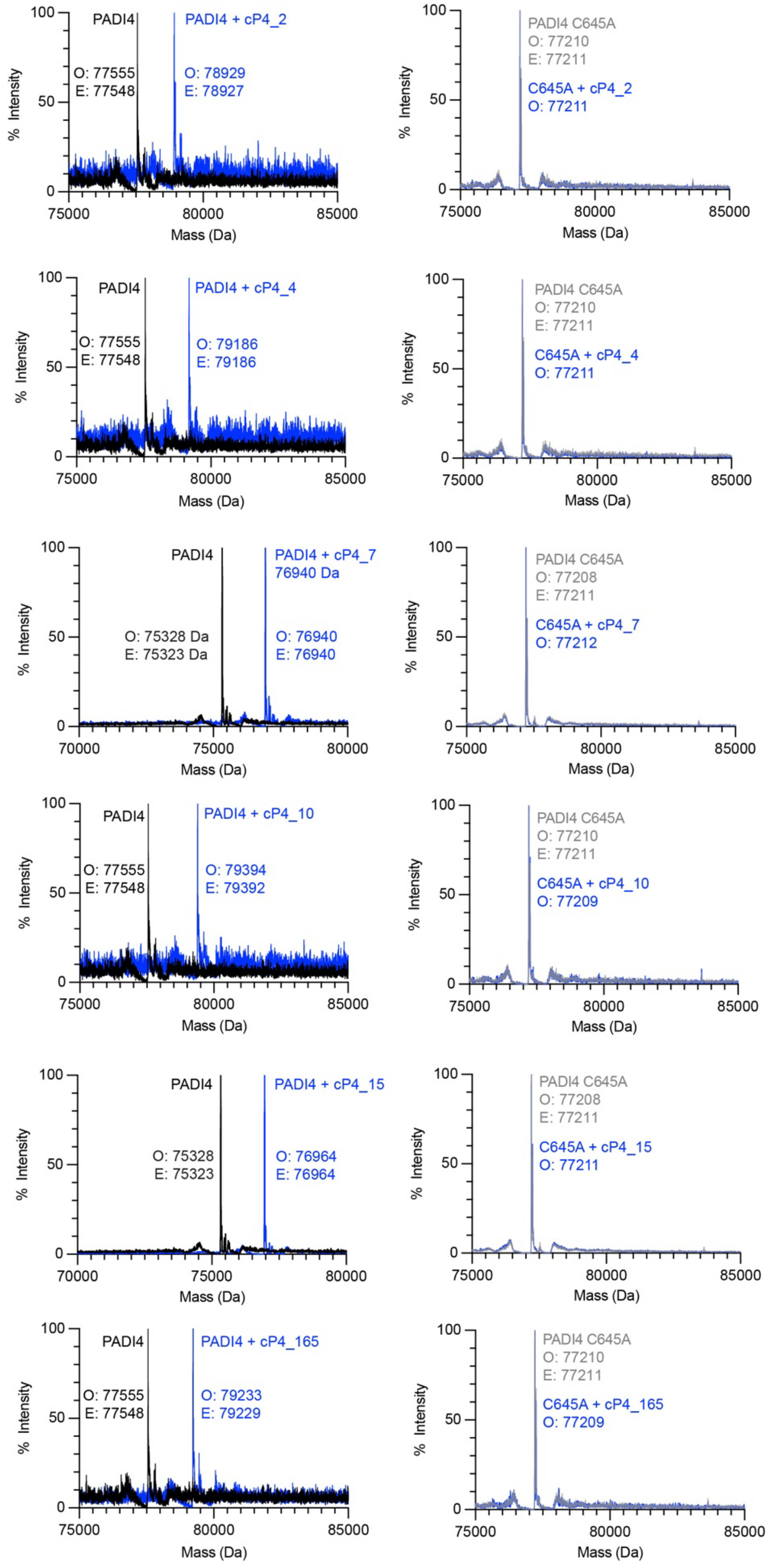

Figure S8. LHS: Intact mass spectrum of PADI4 with (blue) and without (black) incubation with peptides from selection 1, all showing single labelling of PADI4. RHS: Intact mass spectrum of

PADI4 C645A with (blue) and without (grey) incubation with peptide from selection 1, showing no labelling. E – expected molecular weight, O – observed molecular weight.

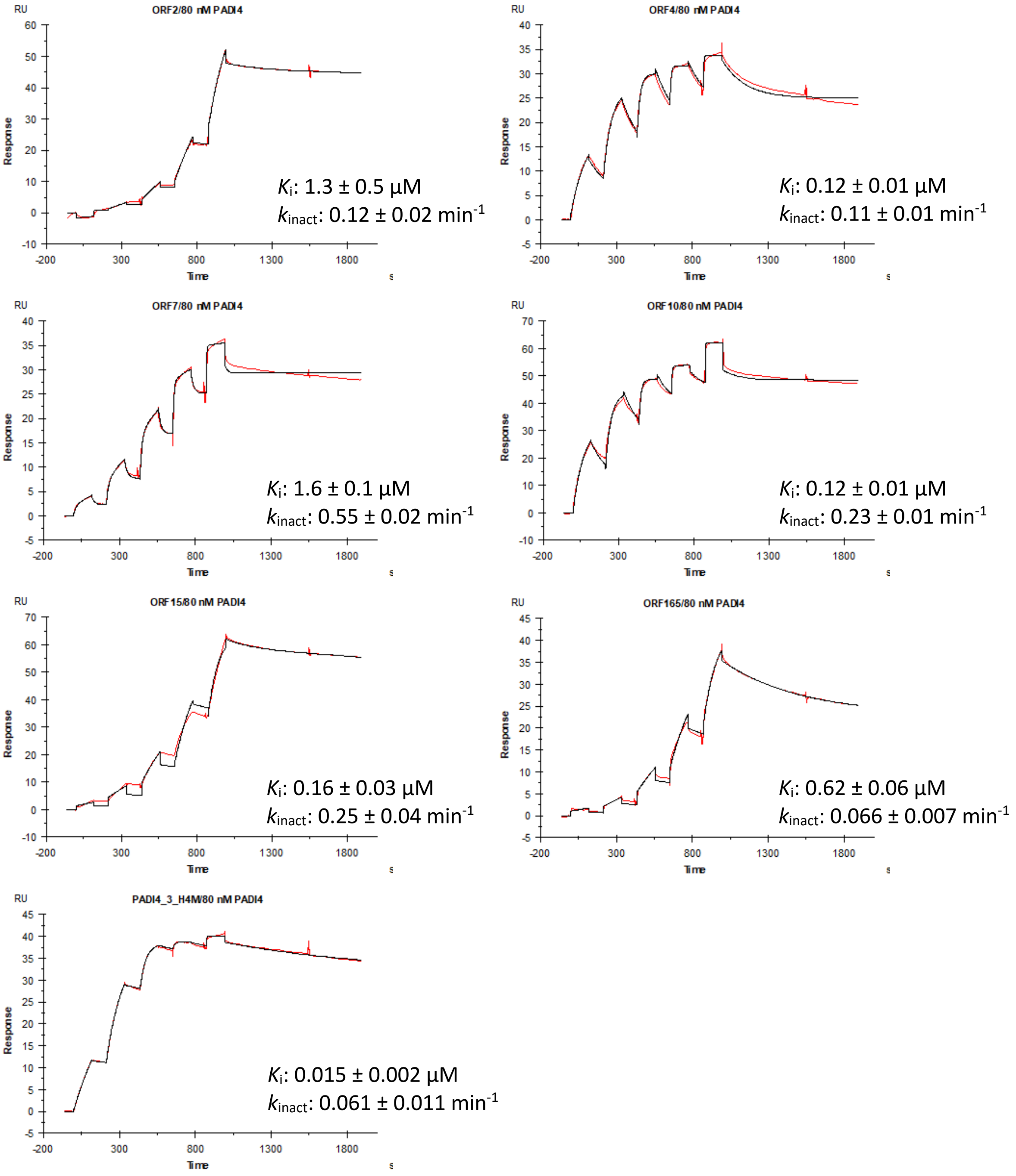

Figure S9. Surface plasmon resonance (SPR) for selection 1 peptides. Representative SPR data for peptide hits (0.12 µM - 10 µM) against PADI4. For cP4_15 peptide concentrations were 0.037 µM – 3 µM. Red lines show experimental data and black lines the fit. Reported values are the mean K_i and k_{inact} ± SEM calculated from three replicates.

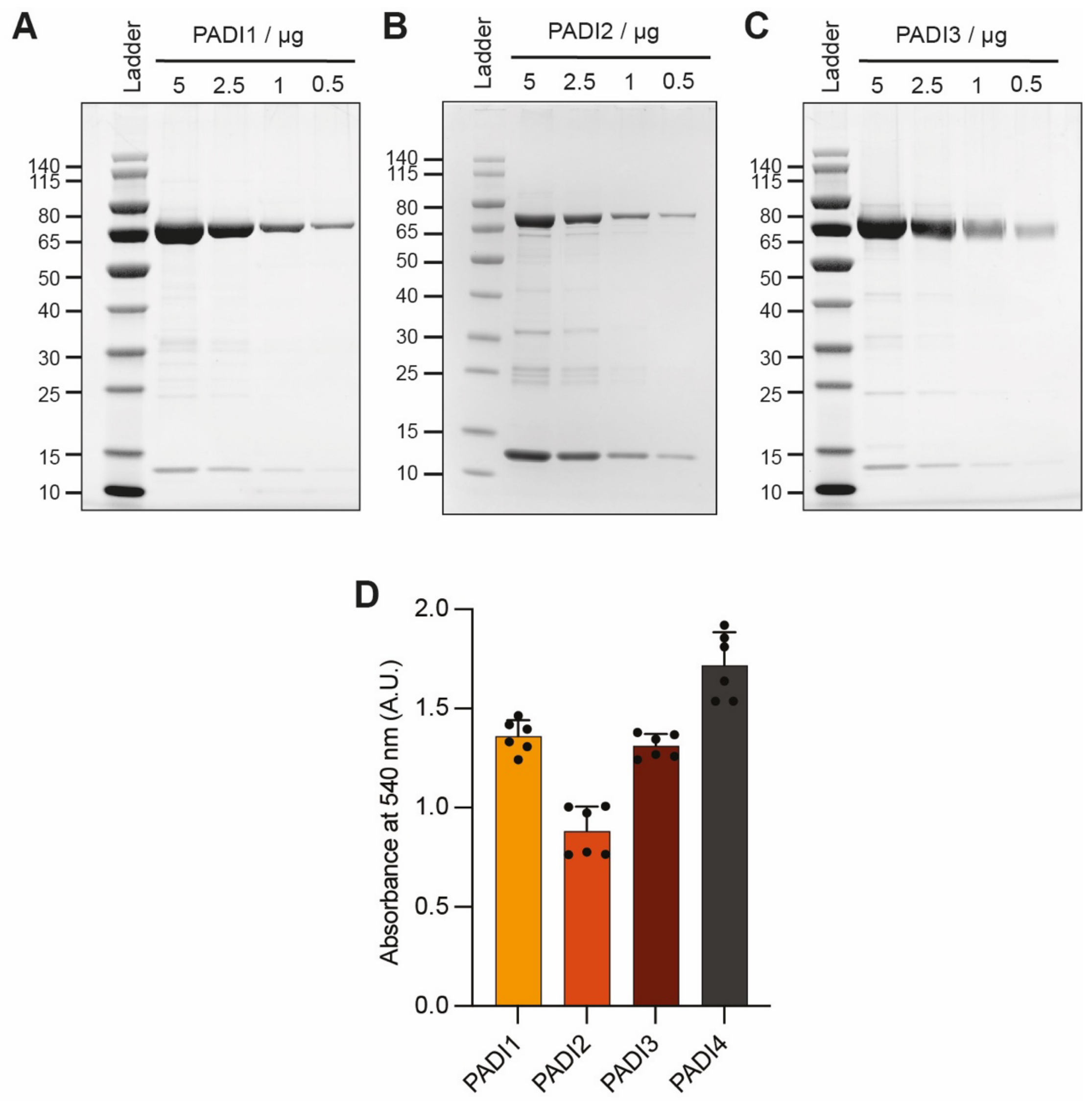

Figure S10. Characterisation of other PADIs. **A** SDS PAGE gel of different quantities of purified PADI1 **B** SDS PAGE gel of different quantities of purified PADI2 **C** SDS PAGE gel of different quantities of PADI3. **D** COLDER assays show that all four PADIs are catalytically active. Individual data points are shown from two repeats each with three replicates and error bars represent the mean ± 1 s.d..

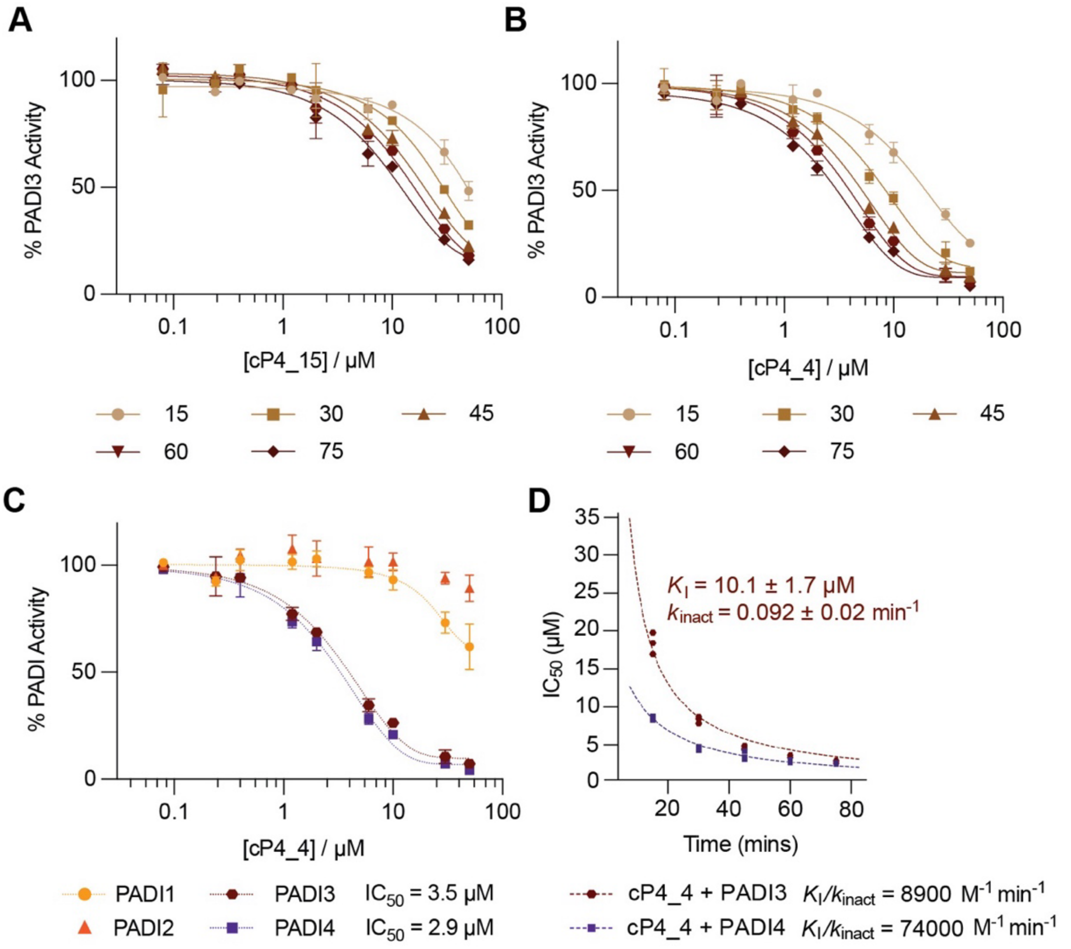

Figure S11. COLDER assays with cP4_4/cP4_15 and PADIs 1-4. **A** and **B** COLDER assays of cP4_15 (**A**) or cP4_4 (**B**) with PADI3. COLDER assays were performed at different peptide concentration (50 – 0.08 µM) in the presence of 10 mM $CaCl_2$ without preincubation with PADI and quenched at 15 min intervals. Data is normalised to activity of each PADI in the presence of 0.1% DMSO. Data shows mean ± SEM of at least two independent replicates. **C** COLDER assays to determine selectivity of cP4_4 for PADI4 over PADIs 1-3. Apparent IC_{50} curves were determined at 15-minute intervals with each of PADIs 1-4. Data for the 60-minute time point is shown. Other time points for PADI3 are shown in Figure S11B. COLDER assays were performed at different peptide concentration (50 – 0.08 µM) in the presence of 10 mM $CaCl_2$ without preincubation with PADI protein. Data is normalised to activity of each PADI in the presence of 0.1% DMSO. Data shows mean ± SEM of at least two independent replicates. **D** COLDER assays to determine K_I and k_{inact}. Apparent IC_{50} values calculated from COLDER assays displayed in part **B** with cP4_4 and PADI3 were determined at 15-minute intervals from three independent replicates and the Krippendorff equation was fitted. Data for PADI4 is included from Fig 3C for comparison.[9]

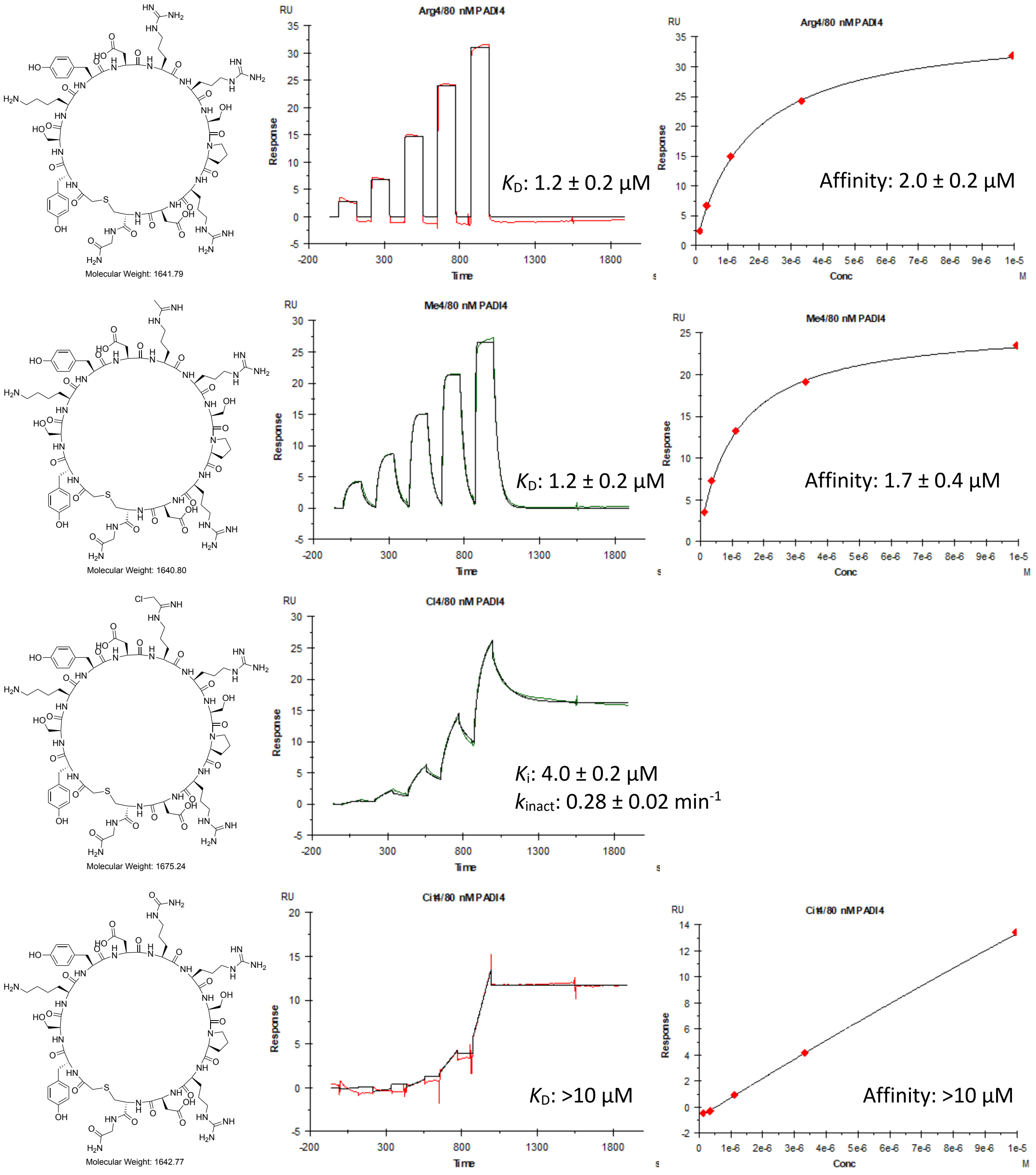

Figure S12. Surface plasmon resonance for cP4_4 variants. Representative SPR data for variants of peptide cP4_4 (0.12 µM - 10 µM) against PADI4. Red/green lines show experimental data and black lines the fit. Reported values are the mean K_D, K_i and k_{inact} ± SEM calculated from three replicates.

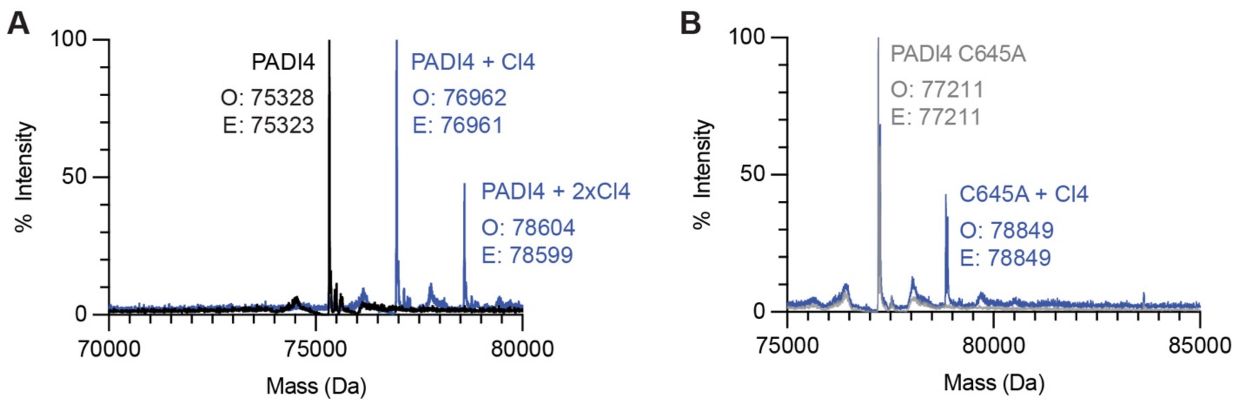

Figure S13. Intact mass spectroscopy with Cl4. **A** Intact mass spectrum of PADI4 with (blue) and without (black) incubation with Cl4, showing single and double labelling of PADI4. **B** Intact mass spectrum of PADI4 C645A with (blue) and without (grey) incubation with Cl4 showing single labelling. E – expected molecular weight, O – observed molecular weight.

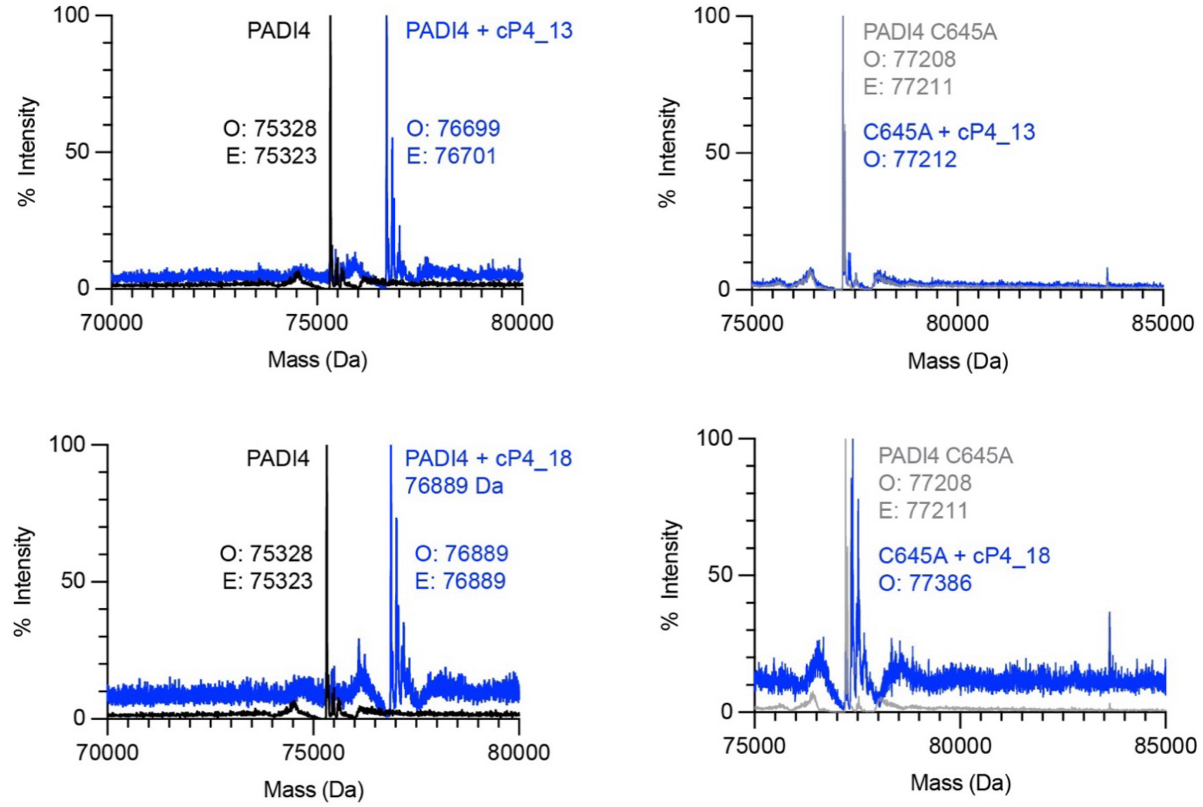

Figure S14. Intact mass spectrometry for selection 2 peptides. LHS: Intact mass spectrum of PADI4 with (blue) and without (black) incubation with cP4_13 or cP4_18, showing single labelling of PADI4. RHS: Intact mass spectrum of PADI4 C645A with (blue) and without (grey) incubation with cP4_13 or cP4_18, showing no labelling. E – expected molecular weight, O – observed molecular weight.

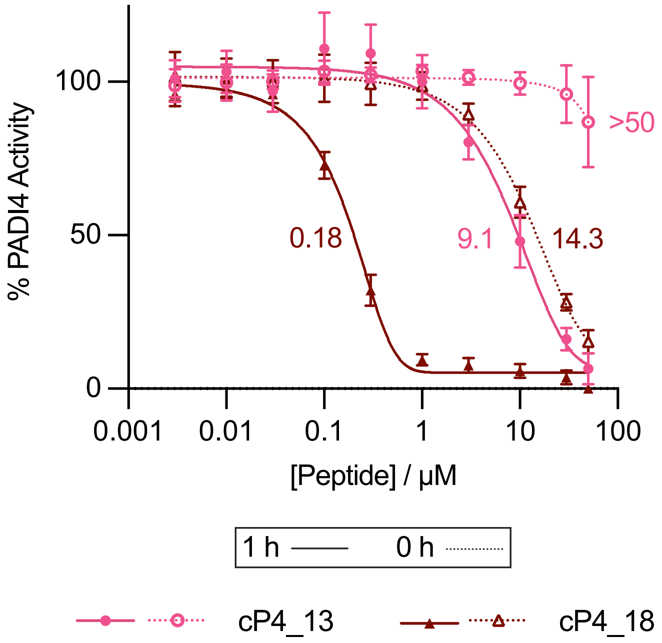

Figure S15. COLDER assays for selection 2 peptides. Inhibition COLDER assays with PADI4 and cP4_13 and cP4_18. COLDER assays were performed at different peptide concentration (50 – 0.003 µM) in the presence of 10 mM $CaCl_2$. Data is normalised to activity of PADI4 in the presence of 0.1% DMSO. Data shows mean ± SEM of two independent replicates. Each replicate was done in triplicate. The mean IC_{50} value is also reported in µM.

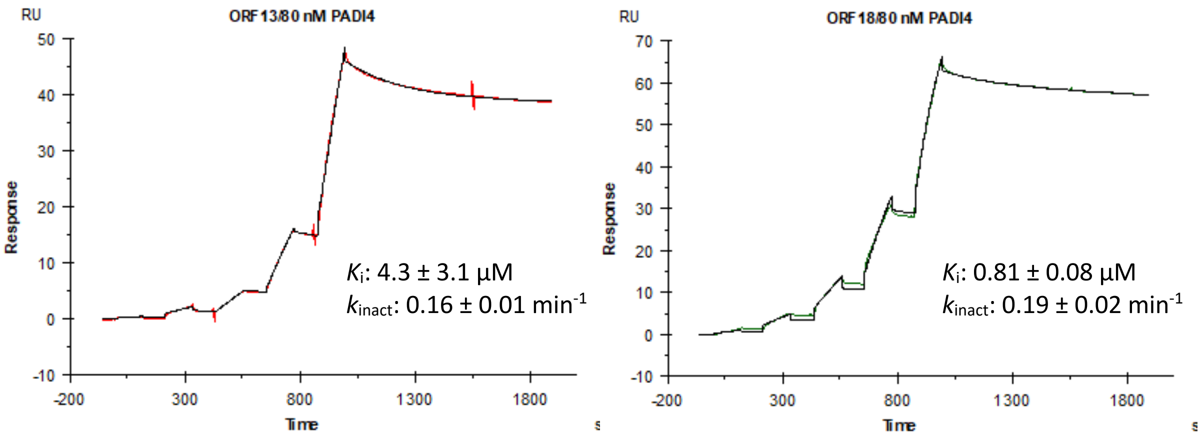

Figure S16. Surface plasmon resonance for selection 2 peptides. Representative SPR data for peptide hits, cP4_13 and cP4_18 (0.12 µM – 10 µM), against PADI4. Red/green lines show experimental data and black lines the fit. Reported values are the mean K_i and k_{inact} ± SEM calculated from three replicates.

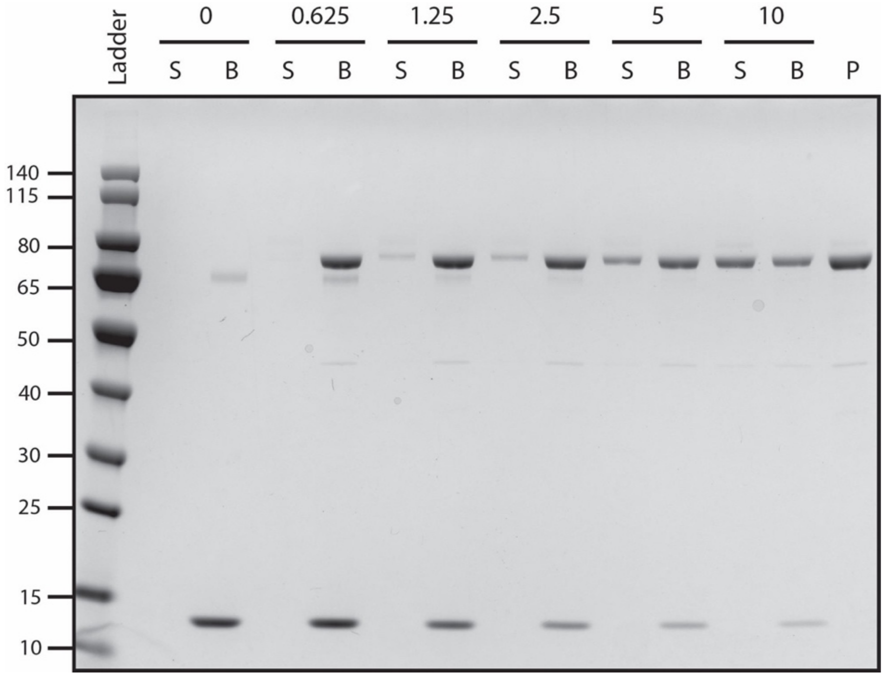

Figure S17. Biotinylation of PADI4 C645A. Confirmation of biotinylation of PADI4 C645A by testing loading onto streptavidin beads. Where S is the supernatant which shows residual PADI4 C645A which wasn't loaded onto the beads, B. 0–10 pmol PADI4 C645A protein/bead was assessed.

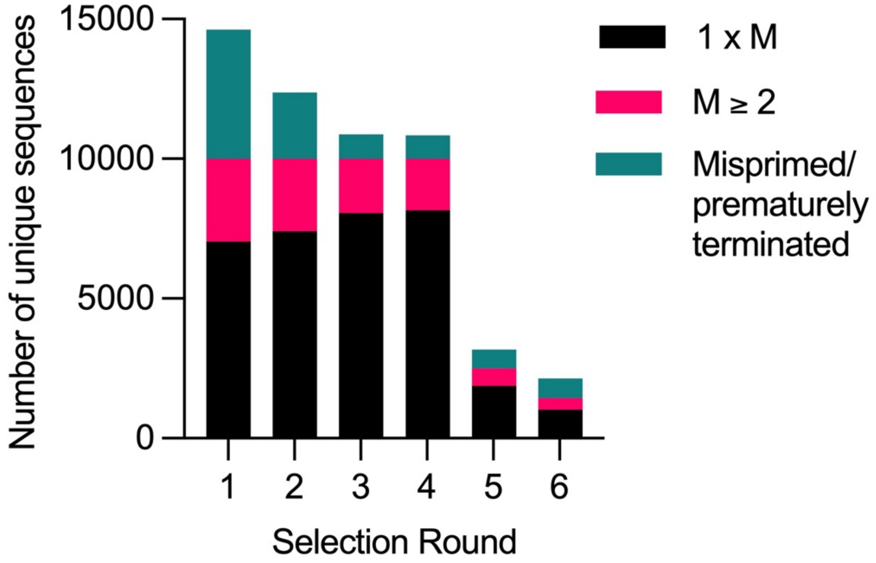

Figure S18. Number of sequences for C645A selection. Analysis of sequences from R1–R6 for C645A selection showing high proportion of misprimed peptide sequences and low levels of warhead-containing peptides.

Me4

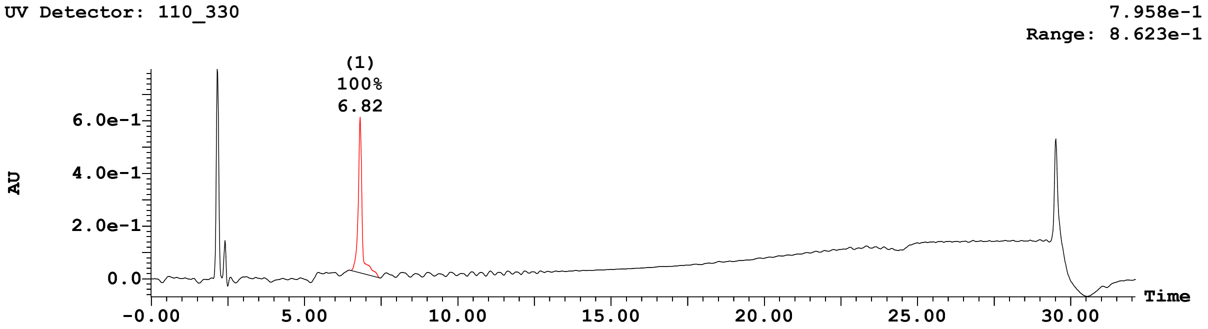

Cl4

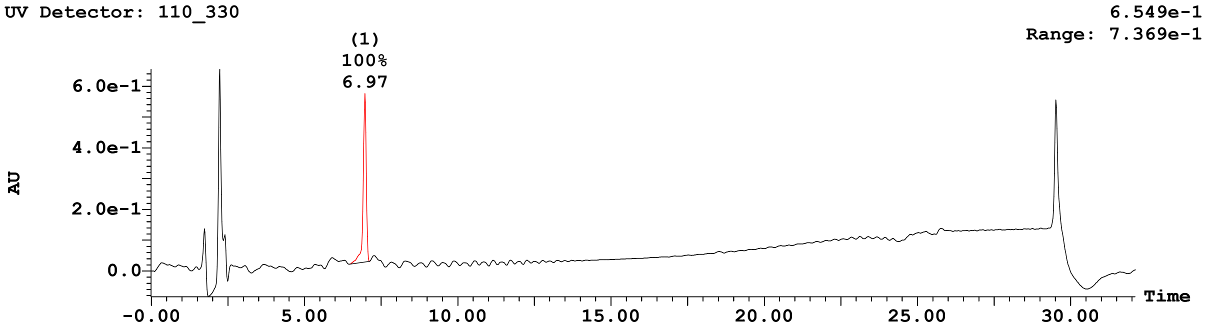

Cit4

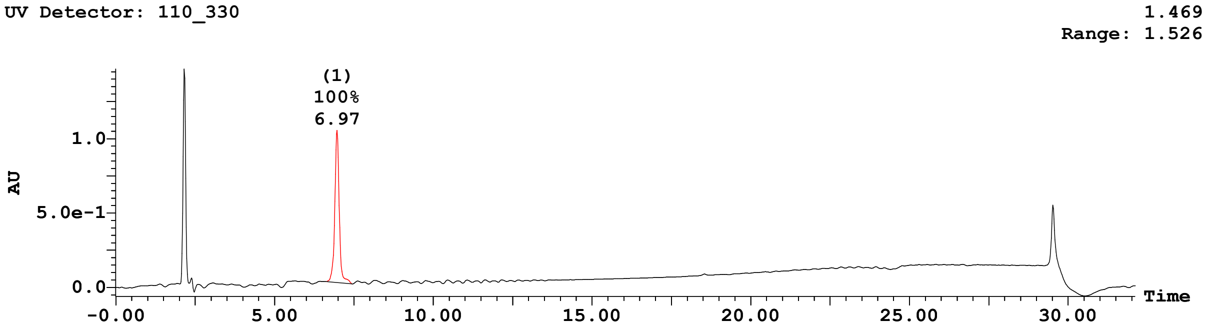

Arg4

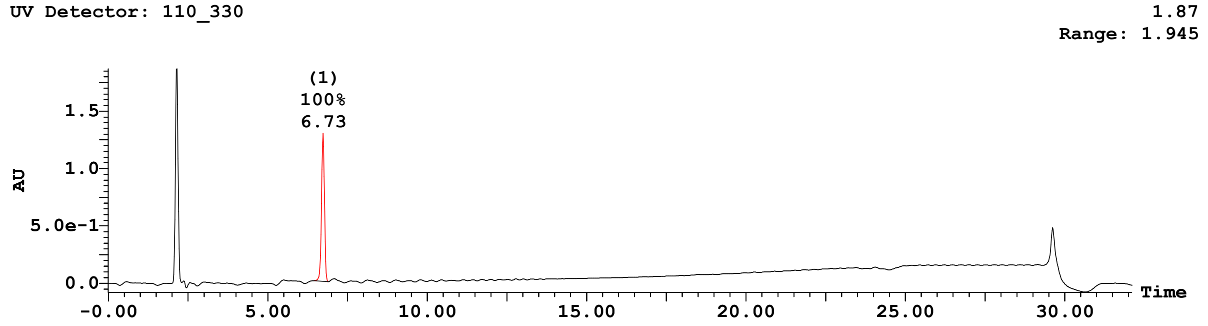

PADI4_3_H4(**2**)

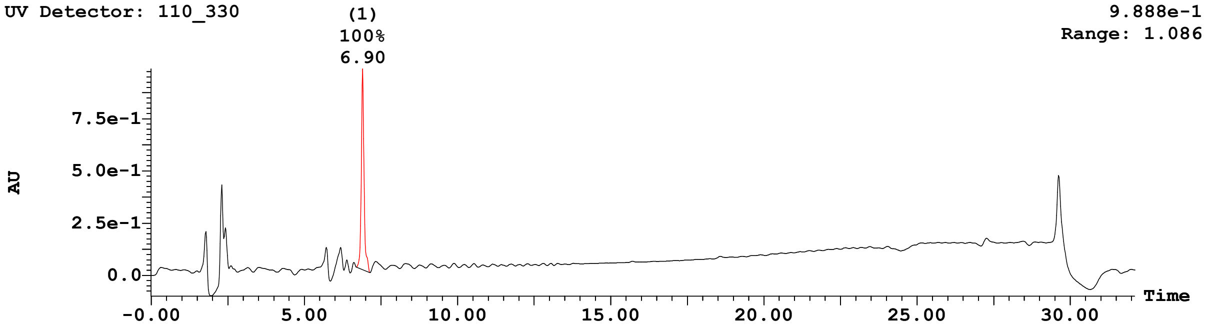

cP4_2

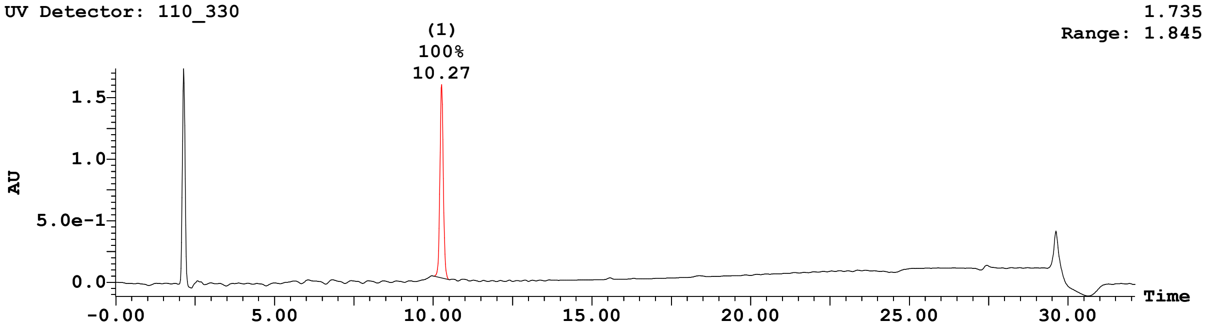

cP4_4

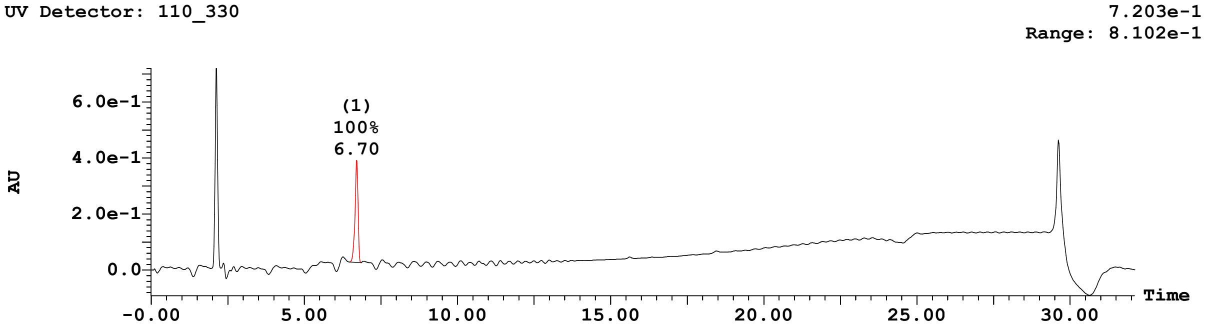

cP4_7

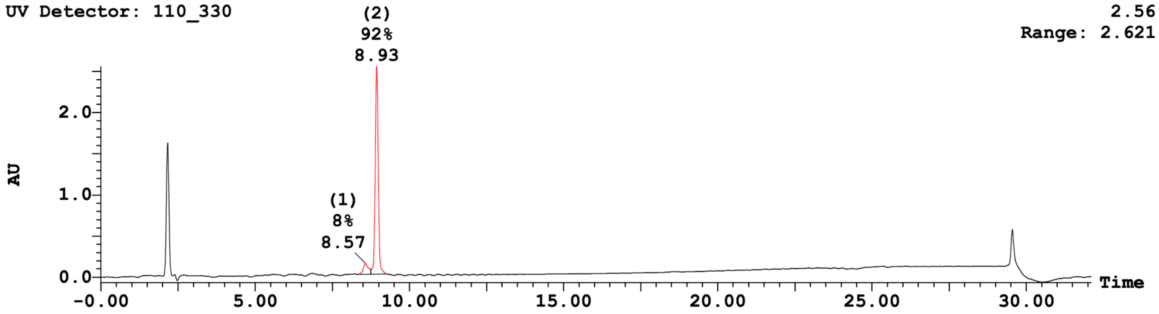

cP4_10

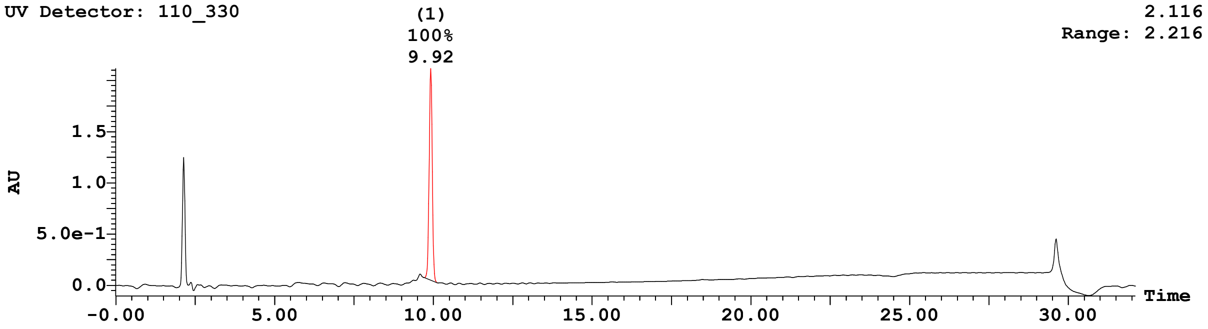

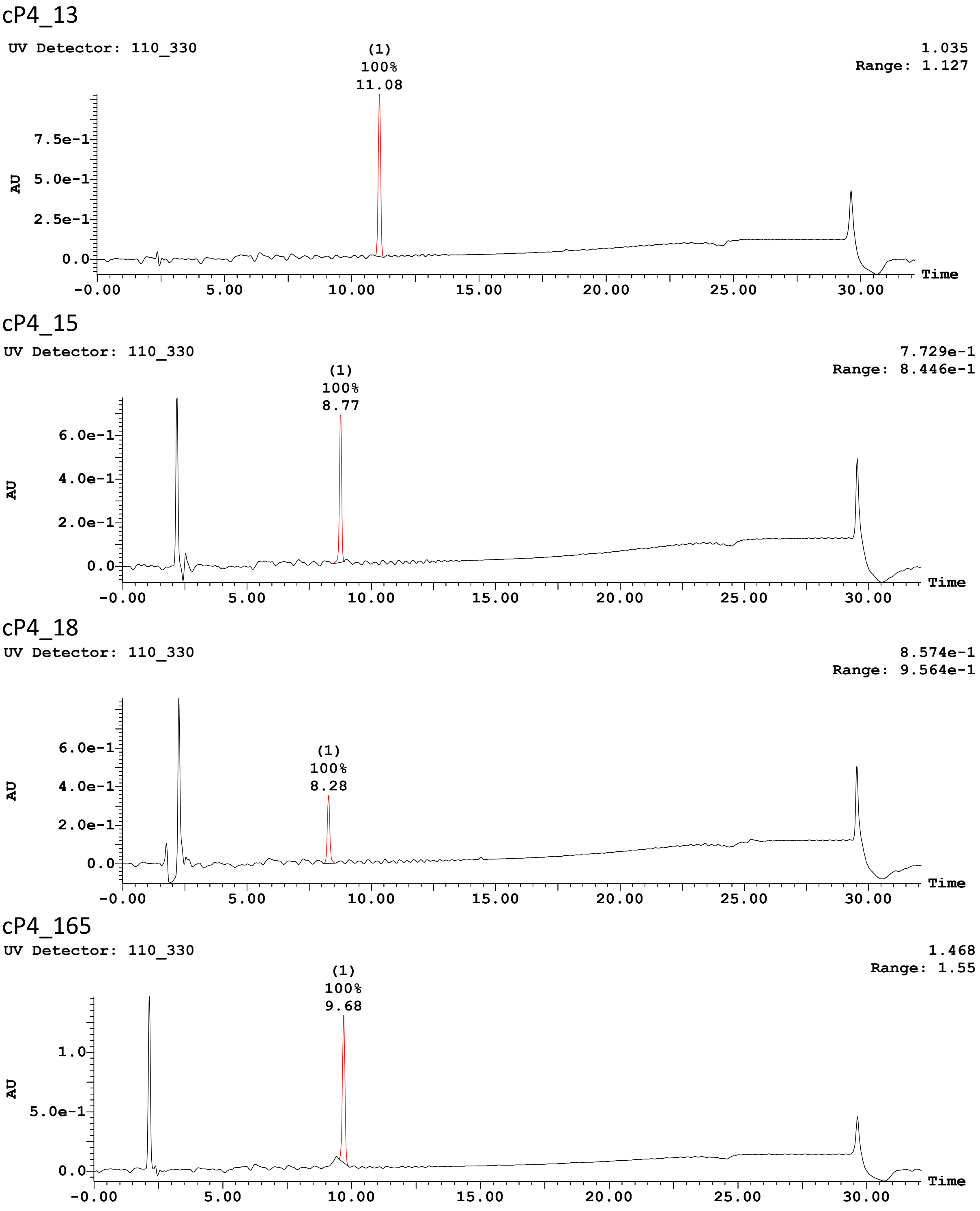

Figure S19. Analytical HPLC traces for PADI4 peptides and variants.

Full-Size, Uncropped Gels

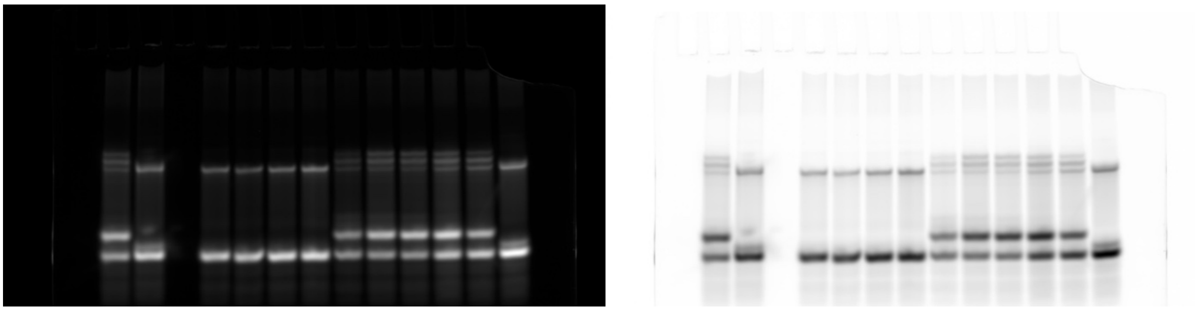

Figure S20. Microhelix assay (Figure 1D)

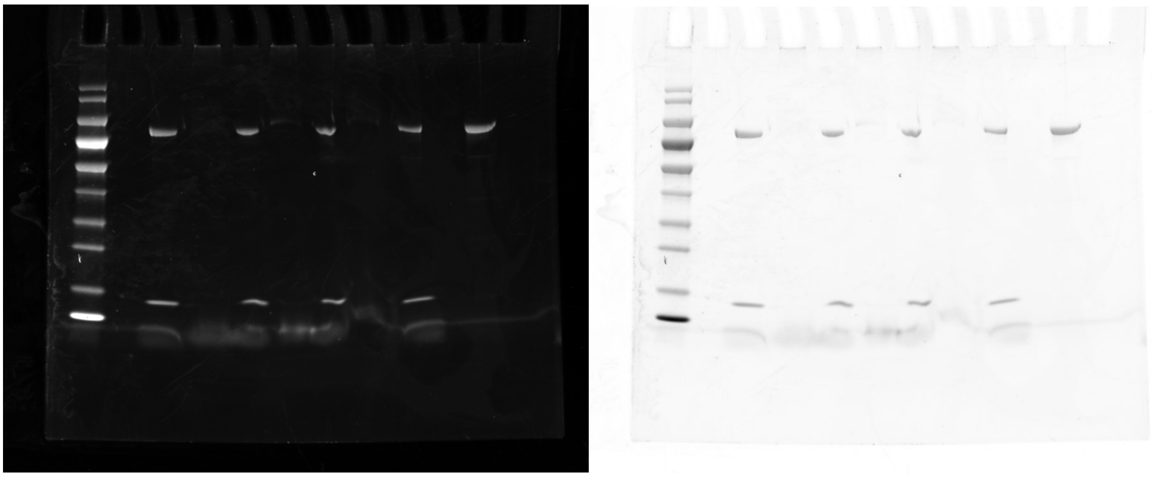

Figure S21. Guanidine washes with PADI4 (Figure S2)

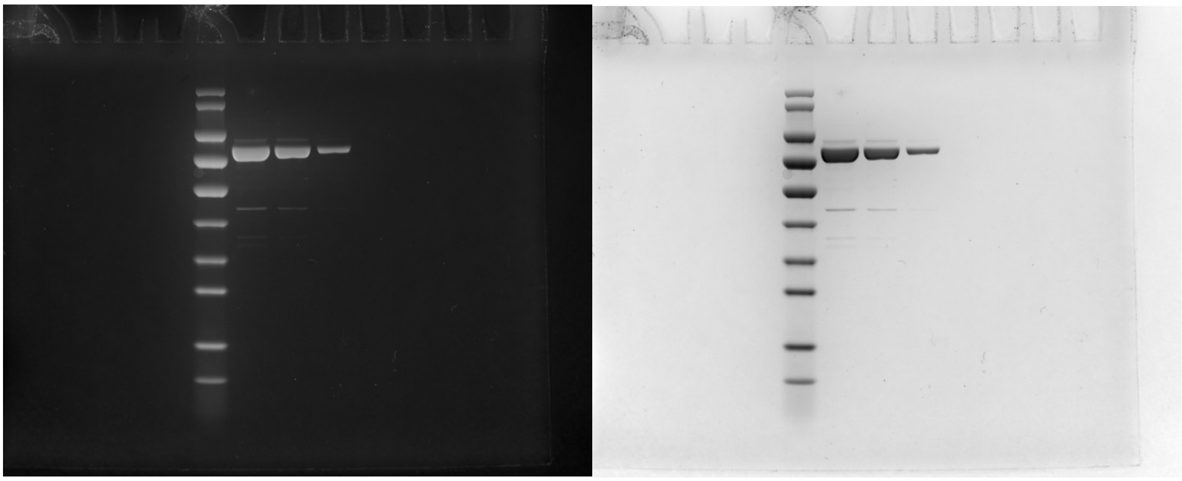

Figure S22. Purity gel of PADI4 C645A (Figure S4A)

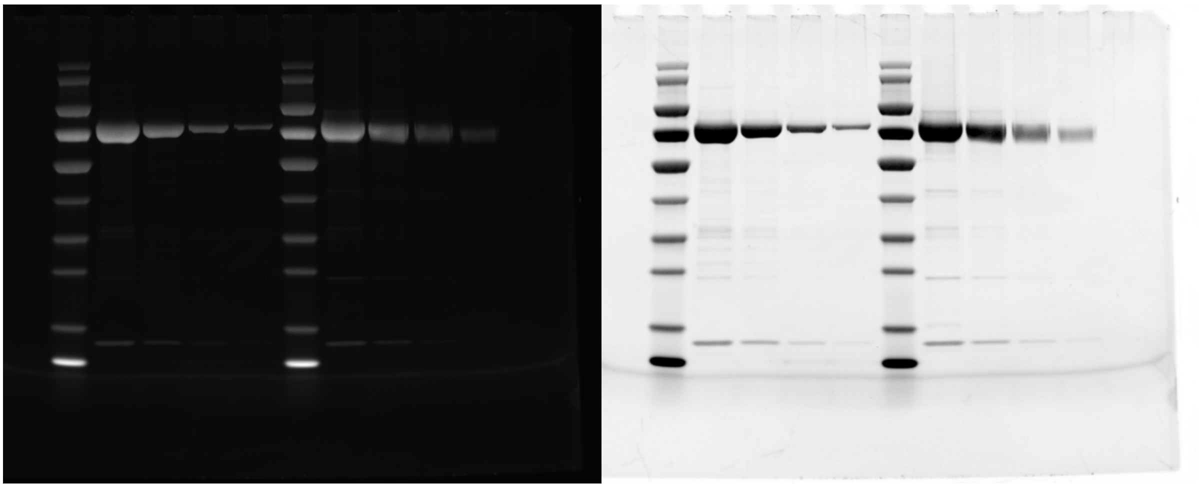

Figure S23. SDS PAGE gels for PADI1 (LHS) and PADI3 (RHS) (Figure S10A, S10C)

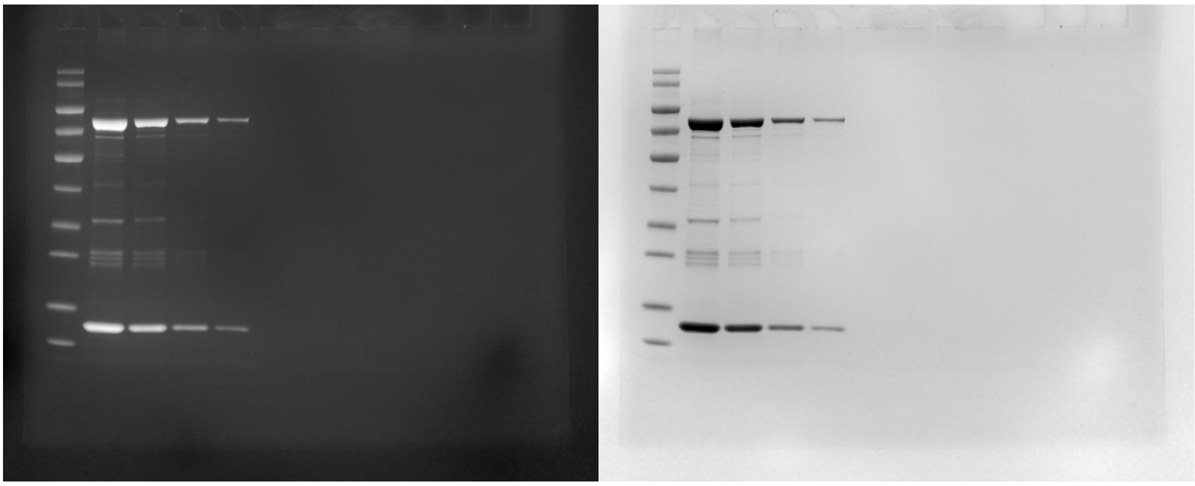

Figure S24. SDS PAGE gel for PADI2 (Figure S10B)

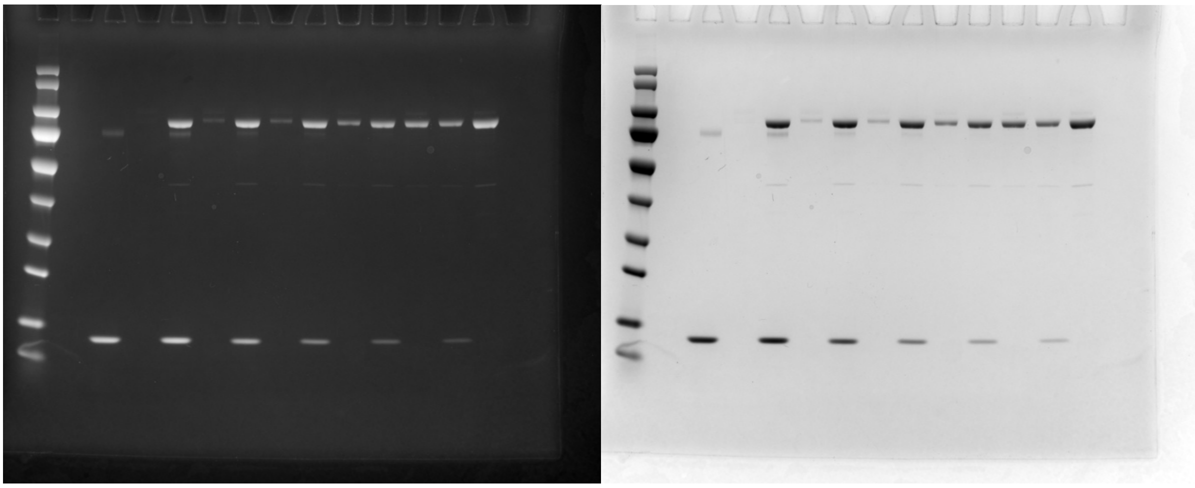

Figure S25. Bead binding assay PADI4 C645A (Figure S17)

Supplementary Tables

Supplementary Table S1. List of oligonucleotides. M represents a 1:1 mixture of C and A, N represents a 1:1:1:1 mixture of all four DNA bases. Gm = 2'-Methoxylated G.

**Oligo ID**	**Sequence**
T7g10M.F46	TAATACGACTCACTATAGGGTTAACTTTAAGAAGGAGATATACATA
CGS3an13.R39	TTTCCGCCCCCCGTCCTAGCTGCCGCTGCCGCTGCCGCA
T7ex5.F22	GGCGTAATACGACTCACTATAG
CGS3an13.R22	TTTCCGCCCCCCGTCCTAGCTG
Ini-5'.F49	GTAATACGACTCACTATAGGCGGGGTGGAGCAGCCTGGTAGCTCGTCGG
Ini-cat.R44	GAACCGACGATCTTCGGGTTATGAGCCCGACGAGCTACCAGGCT
Ini-3'.R38	TGGTTGCGGGGGCCGGATTTGAACCGACGATCTTCGGG
Ini-3'.R20-Me	TGmGTTGCGGGGGCCGGATTT
AsnE2.EX.F (#1-#4)	GTAATACGACTCACTATAGGCTCTGTAGTTCAGTCGGTAGAACGGCGG
AsnE2.EX.R1 (#1)	GCGGCTCTGCAATGACTCGAACACTGGACATACGGAATATGAATCCGCCGTTCTACCG
AsnE2.EX.R2 (#2)	GCGGCTCTGACTGGACTCGAACCAGTGACATACGGAATATGAATCCGCCGTTCTACCG
AsnE2.EX.R3 (#3)	GCGGCTCTGAGGGGACTCGAACCCCTGACATACGGAATATGAATCCGCCGTTCTACCG
AsnE2.EX.R4 (#4)	GCGGCTCTGACGGGACTCGAACCCGCGACATACGGAATATGAATCCGCCGTTCTACCG
AsnE2_PCR_R1 (#1)	TGmGCGGCTCTGCAATG
AsnE2_PCR_R2 (#2)	TGmGCGGCTCTGACTGG
AsnE2_PCR_R3 (#3)	TGmGCGGCTCTGAGGGG
AsnE2_PCR_R4 (#4)	TGmGCGGCTCTGACGGG
HiBiT_EloAsn (MGSMGSVSGWRLFKKISGSGSGS)	GCTGCCGCTGCCGCTGCCAGAGATTTTCTTGAACAGACGCCAACCAGAAACGCTGCCC ATGCTGCCCATATGTATATCTCCTTCTTAAAG
Primer 1 - PADI4_3 template (MRDHHYRHPKYCGSGSGS)	GCTGCCGCTGCCGCTGCCGCAATACTTCGGATGCCTATAATGATGATCCCTCATATGTA TATCTCCTCTTAAAG
Primer 2 - PADI4_3_R2M template (MMDHHYRHPKYCGSGSGS)	GCTGCCGCTGCCGCTGCCGCAATACTTCGGATGCCTATAATGATGATCCATCATATGTA TATCTCCTCTTAAAG
Primer 3 – PADI4_3_H4M template (MRDMHYRHPKYCGSGSGS)	GCTGCCGCTGCCGCTGCCGCAATACTTCGGATGCCTATAATGCATATCCCTCATATGTA TATCTCCTCTTAAAG
stdlib_NNK6_R63	GCTGCCGCTGCCGCTGCCGCAMNNMNNMNNMNNMNNMNNCATATGTATATCTCCT TCTTAAAG
stdlib_NNK7_R66	GCTGCCGCTGCCGCTGCCGCAMNNMNNMNNMNNMNNMNNMNNCATATGTATATC TCCTTCTTAAAG
stdlib_NNK8_R69	GCTGCCGCTGCCGCTGCCGCAMNNMNNMNNMNNMNNMNNMNNMNNCATATGTA TATCTCCTTCTTAAAG
stdlib_NNK9_R72	GCTGCCGCTGCCGCTGCCGCAMNNMNNMNNMNNMNNMNNMNNMNNMNNCATA TGTATATCTCCTTCTTAAAG
stdlib_NNK10_R75	GCTGCCGCTGCCGCTGCCGCAMNNMNNMNNMNNMNNMNNMNNMNN MNNMNNCATATGTATATCTCCTTCTTAAAG
Library DNA sequences	TAATACGACTCACTATAGGGTTAACTTTAAGAAGGAGATATACATATG(NNK)nTGCGGCA GCGGCAGCGGCAGCTAGGACGGGGGGCGGAAA
FAM-MiHx_23b	56-FAM/rArGrGrCrUrCrUrGrUrUrCrGrCrArGrArGrCrCrGrCrCrA
Pu_Linker	[5'Phos]CTCCCGCCCCCCGTCC[SP18][SP18][SP18][SP18][SP18]CC[Puromycin]

pQE80L Vector.REV	GGATCCGTGGTGGTGATGATGG
pQE80L Vector.FOR	AATTCAGGGTACCCAATAAGCTTACAAT
PADI1 Fragment.REV	TGGGTACCCTGAATTTCAGGGCACCATGTTCCACC
PADI1 Fragment.FOR	CACCACCACGGATCCATGGCCCCAAAGAGAGTTGTG
PADI3 Fragment.REV	TGGGTACCCTGAATTTCAGGGCACCATGTTCCACC
PADI3 Fragment.FOR	CACCACCACGGATCCATGTCGCTGCAGAGAATCGTG
PADI4 Fragment.REV	TGGGTACCCTGAATTTTAGGGCACCATGTTCCACCACTTGAAGGAG
PADI4 Fragment.FOR	CACCACCACGGATCCATGGCCCAGGGGACATTGATC
Mut_C645A.F33	CATGGGGAGGTGCACGCCGGCACCAACGTGCGC
Mut_C645A.R33	GCGCACGTTGGTGCCGGCGTGCACCTCCCCATG

Supplementary Table S2. List of peptides synthesised. Where y is D-Tyr which has been chloroacetylated and is cyclised via a thioether bond with the thiol of a Cys side chain. Modified unnatural amino acids are indicated within brackets, where FAO is fluoroacetimidoyl ornithine; ClAO is chloroacetimidoyl ornithine; and HAO is acetimidoyl ornithine; and Cit is citrulline. The optimised HPLC gradient which was run for 15 min during the purification is also indicated (or for 32.5 min for P4_3_H4(**2**)).

**Peptide**	**Sequence**	**Expected MW (g mol^{-1})**	**Observed MW (g mol^{-1})**	**HPLC solvent B gradient X-Y (%)**
PADI4_3_H4(**2**)	yRD(FAO)HYRHPKYCG	1807.0	1806.8	12-15
cP4_2	yIWGL(FAO)D(FAO)SCG	1398.6	1397.8	20-50
cP4_4	ySKYD(FAO)RSPRDCG	1657.7	1659.3	5-35
cP4_7	yVYS(FAO)KEWKYCG	1637.9	1637.5	20-28
cP4_10	yWY(FAO)NWDFNKRCG	1864.1	1865.1	20-40
cP4_13	yIWDL(FAO)DVTCG	1397.5	1397.9	30-44
cP4_15	yLD(FAO)HYSSKLYCG	1660.8	1661.4	20-25
cP4_18	yLN(FAO)ERYRISCG	1585.8	1586.2	15-25
cP4_165	yVY(FAO)DCEWINRAG	1700.9	1701.8	20-35
Arg4	ySKYDRRSPRDCG	1643.8	1644.1	10-25
Cit4	ySKYD(Cit)RSPRDCG	1642.8	1643.2	10-25
Cl4	ySKYD(ClAO)RSPRDCG	1675.2	1674.5	11-19
Met4	ySKYD(HAO)RSPRDCG	1640.8	1639.3	10-25

Supplementary Table S3: HPLC gradients used for the purification of all peptides. Where X and Y are the optimised gradients for solvent B, defined in Table S2 for all peptides. The gradients follow the same template except for the purification of P4_3_H4(**2**) and cP4_4 which both used alternative gradients.

<table>
<tr><th rowspan="2">Time (min)</th><th rowspan="2">Flow rate (ml min^{-1})</th><th colspan="3">HPLC solvent B gradient (%)</th></tr>
<tr><th>All peptides</th><th>P4_3_H4(2)</th><th>cP4_4</th></tr>
<tr><td>0</td><td>24</td><td>10</td><td>10</td><td>5</td></tr>
<tr><td>2.5</td><td>24</td><td>10</td><td>12</td><td>5</td></tr>
<tr><td>5</td><td>24</td><td>X</td><td rowspan="6">Gradient</td><td>5</td></tr>
<tr><td>20</td><td>24</td><td>Y</td><td>35</td></tr>
<tr><td>22</td><td>24</td><td>98</td><td>98</td></tr>
<tr><td>27</td><td>24</td><td>98</td><td>98</td></tr>
<tr><td>28</td><td>24</td><td>10</td><td>5</td></tr>
<tr><td>32</td><td>24</td><td>10</td><td>5</td></tr>
<tr><td>35</td><td>24</td><td>-</td><td>45</td><td>-</td></tr>
<tr><td>37</td><td>24</td><td>-</td><td>98</td><td>-</td></tr>
<tr><td>42</td><td>24</td><td>-</td><td>98</td><td>-</td></tr>
<tr><td>43</td><td>24</td><td>-</td><td>10</td><td>-</td></tr>
<tr><td>47</td><td>24</td><td>-</td><td>10</td><td>-</td></tr>
</table>

Supplementary Table S4: Covalent peptide SPR data from each replicate where k_1 and k_{-1} are the reversible rate constants. k_2 is the is the inactivation rate constant which is reported as k_i in Table 1 and Table 2.

**Peptide**	**k_1 (M^{-1} s^{-1})**			**k_{-1} (s^{-1})**			**k_2 / k_{inact} (s^{-1})**		
cP4_2	430	690	360	5.2E-04	3.1E-04	8.1E-04	0.0014	0.0026	0.0019
cP4_4	4.8E+04	4.4E+04	4.3E+04	0.0055	0.0056	0.0049	0.0017	0.0019	0.0017
cP4_7	1.7E+04	3.3E+04	2.4E+04	0.027	0.047	0.042	0.0086	0.0094	0.0094
cP4_10	7.5E+04	6.9E+04	7.5E+04	0.0097	0.0066	0.0092	0.0041	0.0034	0.0041
cP4_13	6.7	49	180	7.0E-04	8.8E-04	8.8E-04	0.0025	0.0029	0.0025
cP4_15	4800	8100	5100	5.1E-04	0.0016	8.8E-04	0.0028	0.0051	0.0047
cP4_18	380	700	710	3.5E-04	3.3E-04	5.0E-04	0.0027	0.0019	0.0037
cP4_165	1.5E+03	1.2E+03	1.6E+03	9.7E-04	8.7E-04	8.0E-04	0.0013	0.0011	9.1E-04
P4_3_H4(**2**)	3.0E+04	3.3E+04	2.42E+04	4.5E-04	5.7E-04	3.0E-04	0.0010	0.0013	0.00072

Supplementary References

1. Goto, Y., Katoh, H & Suga, H. Preparation of materials for flexizyme reactions and genetic code reprogramming. *Nat. Protoc. Exch.* **6**, 779–790 (2011).
2. Iwane, Y., Kimura, H., Katoh, T. & Suga, H. Uniform affinity-tuning of N-methyl-aminoacyl-tRNAs to EF-Tu enhances their multiple incorporation. *Nucleic Acids Res.* **49**, 10807–10817 (2021).
3. Hughes, L. R. *et al.* Quinazoline antifolate thymidylate synthase inhibitors: alkyl, substituted alkyl, and aryl substituents in the C-2 position. *J. Med. Chem.* **33**, 3060–3067 (1990).
4. Preparation of N1-Phenylacetamidine 4-Bromobenzoate Using 2,2,2-Trichloroethyl Acetimidate Hydrochloride. *Org. Synth.* **90**, 174–181 (2013).
5. Arnáiz, F. J. A Convenient Way to Generate Hydrogen Chloride in the Freshman Lab. *J. Chem. Educ.* **72**, 1139 (1995).
6. Stillings, M. R., Welbourn, A. P. & Walter, D. S. Substituted 1,3,4-thiadiazoles with anticonvulsant activity. 2. Aminoalkyl derivatives. *J. Med. Chem.* **29**, 2280–2284 (1986).
7. Jones, J. E. *et al.* Synthesis and Screening of a Haloacetamidine Containing Library To Identify PAD4 Selective Inhibitors. *ACS Chem. Biol.* **7**, 160–165 (2012).
8. Bertran, M. T. *et al.* A cyclic peptide toolkit reveals mechanistic principles of peptidylarginine deiminase IV regulation. *Nat. Commun.* **15**, 9746 (2024).
9. Krippendorff, B.-F., Neuhaus, R., Lienau, P., Reichel, A. & Huisinga, W. Mechanism-Based Inhibition: Deriving KI and kinact Directly from Time-Dependent IC50 Values. *J. Biomol. Screen.* **14**, 913–923 (2009).

hPADI1 plasmid

GATCGTATCGGAGAGCTTAAATCGGGCGATGCCTGTGTTGCAGAATACCAGCAGGCTGGCCGTGGTCGCCGGGGGCGGAAATGGTTTTCGCCTTTTGGCGCAAACTT
ATATTTGTCGATGTTCTGGCGTCTGGAACAAGGCCCGGCGGCGGCGATTGGTTTAAGTCTGGTTATCGGTATCGTGATGGCGGAAGTATTACGCAAGCTGGGAGCAG
ATAAAGTTCGTGTCAAATGGCCTAATGACCTCTATCTGCAGGATCGCAAGCTGGCAGGCATTCTTGTGGAGCTGACTGGCAAAACTGGCGATGCGGCGCAAATAGTC
ATTGGAGCCGGGATCAACATGGCAATGCGCCGTGTTGAAGAGAGTGTCGTTAATCAGGGGTGGATCACGCTGCAGGAAGCGGGGATCAATCTCGATCGTAATACGTT
GGCGGCCATGCTAATACGTGAATTACGTGCTGCGTTGGAACTCTTCGAACAAGAAGGATTGGCACCTTATCTGTCGCGCTGGGAAAAGCTGGATAATTTTATTAATC
GCCCAGTGAAACTTATCATTGGTGATAAAGAAATATTTGGCATTTCACGCGGAATAGACAAACAGGGGGCTTTATTACTTGAGCAGGATGGAATAATAAAACCCTGG
ATGGGCGGTGAAATATCCCTGCGTAGTGCAGAAAAATAAGCGGCCGCAGCTTAATTAGCTGAGCTTGGACTCCTGTTGATAGATCCAGTAATGACCTCAGAACTCCA
TCTGGATTTGTTCAGAACGCTCGGTTGCCGCCGGGCGTTTTTTATTGGTGAGAATCCAAGCTAGCTTGGCGAGATTTTCAGGAGCTAAGGAAGCTAAAATGGAGAAA
AAAATCACTGGATATACCACCGTTGATATATCCCAATGGCATCGTAAAGAACATTTTGAGGCATTTCAGTCAGTTGCTCAATGTACCTATAACCAGACCGTTCAGCT
GGATATTACGGCCTTTTTAAAGACCGTAAAGAAAAATAAGCACAAGTTTTATCCGGCCTTTATTCACATTCTTGCCCGCCTGATGAATGCTCATCCGGAATTTCGTA
TGGCAATGAAAGACGGTGAGCTGGTGATATGGGATAGTGTTCACCCTTGTTACACCGTTTTCCATGAGCAAACTGAAACGTTTTCATCGCTCTGGAGTGAATACCAC
GACGATTTCCGGCAGTTTCTACACATATATTCGCAAGATGTGGCGTGTTACGGTGAAAACCTGGCCTATTTCCCTAAAGGGTTTATTGAGAATATGTTTTTCGTCTC
AGCCAATCCCTGGGTGAGTTTCACCAGTTTTGATTTAAACGTGGCCAATATGGACAACTTCTTCGCCCCCGTTTTCACCATGGGCAAATATTATACGCAAGGCGACA
AGGTGCTGATGCCGCTGGCGATTCAGGTTCATCATGCCGTTTGTGATGGCTTCCATGTCGGCAGAATGCTTAATGAATTACAACAGTACTGCGATGAGTGGCAGGGC
GGGGCGTAATTTTTTTAAGGCAGTTATTGGTGCCCTTAAACGCCTGGGGTAATGACTCTCTAGCTTGAGGCATCAAATAAAACGAAAGGCTCAGTCGAAAGACTGGG
CCTTTCGTTTTATCTGTTGTTTGTCGGTGAACGCTCTCCTGAGTAGGACAAATCCGCCCTCTAGATTACGTGCAGTCGATGATAAGCTGTCAAACATGAGAATTGTG
CCTAATGAGTGAGCTAACTTACATTAATTGCGTTGCGCTCACTGCCCGCTTTCCAGTCGGGAAACCTGTCGTGCCAGCTGCATTAATGAATCGGCCAACGCGCGGGG
AGAGGCGGTTTGCGTATTGGGCGCCAGGGTGGTTTTTCTTTTCACCAGTGAGACGGGCAACAGCTGATTGCCCTTCACCGCCTGGCCCTGAGAGAGTTGCAGCAAGC
GGTCCACGCTGGTTTGCCCCAGCAGGCGAAAATCCTGTTTGATGGTGGTTAACGGCGGGATATAACATGAGCTGTCTTCGGTATCGTCGTATCCCACTACCGAGATA
TCCGCACCAACGCGCAGCCCGGACTCGGTAATGGCGCGCATTGCGCCCAGCGCCATCTGATCGTTGGCAACCAGCATCGCAGTGGGAACGATGCCCTCATTCAGCAT
TTGCATGGTTTGTTGAAAACCGGACATGGCACTCCAGTCGCCTTCCCGTTCCGCTATCGGCTGAATTTGATTGCGAGTGAGATATTTATGCCAGCCAGCCAGACGCA
GACGCGCCGAGACAGAACTTAATGGGCCCGCTAACAGCGCGATTTGCTGGTGACCCAATGCGACCAGATGCTCCACGCCCAGTCGCGTACCGTCTTCATGGGAGAAA
ATAATACTGTTGATGGGTGTCTGGTCAGAGACATCAAGAAATAACGCCGGAACATTAGTGCAGGCAGCTTCCACAGCAATGGCATCCTGGTCATCCAGCGGATAGTT
AATGATCAGCCCACTGACGCGTTGCGCGAGAAGATTGTGCACCGCCGCTTTACAGGCTTCGACGCCGCTTCGTTCTACCATCATCACCACCACGCTGGCACCCAGTT
GATCGGCGCGAGATTTAATCGCCGCGACAATTTGCGACGGCGCGTGCAGGGCCAGACTGGAGGTGGCAACGCCAATCAGCAACGACTGTTTGCCCGCCAGTTGTTGT
GCCACGCGGTTGGGAATGTAATTCAGCTCCGCCATCGCCGCTTCCACTTTTTCCCGCGTTTTCGCAGAAACGTGGCTGGCCTGGTTCACCACGCGGGAAACGGTCTG
ATAAGAGACACCGGCATACTCTGCGACATCGTATAACGTTACTGGTTTCACATTCACCACCCTGAATTGACTCTCTTCCGGGCGCTATCATGCCATACCGCGAAAGG
TTTTGCACCATTCGATGGTGTCGGAATTTCGGGCAGCGTTGGGTCCTGGCCACGGGTGCGCATGATCTAGAGCTGCCTCGCGCGTTTCGGTGATGACGGTGAAAACC
TCTGACACATGCAGCTCCCGGAGACGGTCACAGCTTGTCTGTAAGCGGATGCCGGGAGCAGACAAGCCCGTCAGGGCGCGTCAGCGGGTGTTGGCGGGTGTCGGGGC
GCAGCCATGACCCAGTCACGTAGCGATAGCGGAGTGTATACTGGCTTAACTATGCGGCATCAGAGCAGATTGTACTGAGAGTGCACCATATGCGGTGTGAAATACCG
CACAGATGCGTAAGGAGAAAATACCGCATCAGGCGCTCTTCCGCTTCCTCGCTCACTGACTCGCTGCGCTCGGTCGTTCGGCTGCGGCGAGCGGTATCAGCTCACTC
AAAGGCGGTAATACGGTTATCCACAGAATCAGGGGATAACGCAGGAAAGAACATGTGAGCAAAAGGCCAGCAAAAGGCCAGGAACCGTAAAAAGGCCGCGTTGCTGG
CGTTTTTCCATAGGCTCCGCCCCCCTGACGAGCATCACAAAAATCGACGCTCAAGTCAGAGGTGGCGAAACCCGACAGGACTATAAAGATACCAGGCGTTTCCCCCT
GGAAGCTCCCTCGTGCGCTCTCCTGTTCCGACCCTGCCGCTTACCGGATACCTGTCCGCCTTTCTCCCTTCGGGAAGCGTGGCGCTTTCTCATAGCTCACGCTGTAG
GTATCTCAGTTCGGTGTAGGTCGTTCGCTCCAAGCTGGGCTGTGTGCACGAACCCCCCGTTCAGCCCGACCGCTGCGCCTTATCCGGTAACTATCGTCTTGAGTCCA
ACCCGGTAAGACACGACTTATCGCCACTGGCAGCAGCCACTGGTAACAGGATTAGCAGAGCGAGGTATGTAGGCGGTGCTACAGAGTTCTTGAAGTGGTGGCCTAAC
TACGGCTACACTAGAAGGACAGTATTTGGTATCTGCGCTCTGCTGAAGCCAGTTACCTTCGGAAAAAGAGTTGGTAGCTCTTGATCCGGCAAACAAACCACCGCTGG
TAGCGGTGGTTTTTTTGTTTGCAAGCAGCAGATTACGCGCAGAAAAAAAGGATCTCAAGAAGATCCTTTGATCTTTTCTACGGGGTCTGACGCTCAGTGGAACGAAA
ACTCACGTTAAGGGATTTTGGTCATGAGATTATCAAAAAGGATCTTCACCTAGATCCTTTTAAATTAAAAATGAAGTTTTAAATCAATCTAAAGTATATATGAGTAA
ACTTGGTCTGACAGTTACCAATGCTTAATCAGTGAGGCACCTATCTCAGCGATCTGTCTATTTCGTTCATCCATAGTTGCCTGACTCCCCGTCGTGTAGATAACTAC
GATACGGGAGGGCTTACCATCTGGCCCCAGTGCTGCAATGATACCGCGAGACCCACGCTCACCGGCTCCAGATTTATCAGCAATAAACCAGCCAGCCGGAAGGGCCG
AGCGCAGAAGTGGTCCTGCAACTTTATCCGCCTCCATCCAGTCTATTAATTGTTGCCGGGAAGCTAGAGTAAGTAGTTCGCCAGTTAATAGTTTGCGCAACGTTGTT
GCCATTGCTACAGGCATCGTGGTGTCACGCTCGTCGTTTGGTATGGCTTCATTCAGCTCCGGTTCCCAACGATCAAGGCGAGTTACATGATCCCCCATGTTGTGCAA
AAAAGCGGTTAGCTCCTTCGGTCCTCCGATCGTTGTCAGAAGTAAGTTGGCCGCAGTGTTATCACTCATGGTTATGGCAGCACTGCATAATTCTCTTACTGTCATGC
CATCCGTAAGATGCTTTTCTGTGACTGGTGAGTACTCAACCAAGTCATTCTGAGAATAGTGTATGCGGCGACCGAGTTGCTCTTGCCCGGCGTCAATACGGGATAAT
ACCGCGCCACATAGCAGAACTTTAAAAGTGCTCATCATTGGAAAACGTTCTTCGGGGCGAAAACTCTCAAGGATCTTACCGCTGTTGAGATCCAGTTCGATGTAACC
CACTCGTGCACCCAACTGATCTTCAGCATCTTTTACTTTCACCAGCGTTTCTGGGTGAGCAAAAACAGGAAGGCAAAATGCCGCAAAAAAGGGAATAAGGGCGACAC
GGAAATGTTGAATACTCATACTCTTCCTTTTTCAATATTATTGAAGCATTTATCAGGGTTATTGTCTCATGAGCGGATACATATTTGAATGTATTTAGAAAAATAAA
CAAATAGGGGTTCCGCGCACATTTCCCCGAAAAGTGCCACCTGACGTCTAAGAAACCATTATTATCATGACATTAACCTATAAAAATAGGCGTATCACGAGGCCCTT
TCGTCTTCACCTCGATTCGTCTTCACCTCGAGAAATCATAAAAAATTTATTTGCTTTGTGAGCGGATAACAATTATAATAGATTCAATTGTGAGCGGATAACAATTT
CACACAGAATTTATTAAAGAGGAGAAATTACATATGTCCGGCCTGAACGACATCTTCGAGGCTCAGAAAATCGAATGGCACGAAGGTCACCATCATCACCACCACGG
ATCCATGGCCCCAAAGAGAGTTGTGCAGCTGTCCCTGAAGATGCCTACCCATGCCGTGTGTGTGGTGGGAGTCGAGGCACATGTGGACATTCACAGTGATGTGCCCA
AGGGTGCCAACAGCTTCAGGGTCTCTGGAAGCTCCGGGGTGGAGGTCTTCATGGTCTACAACCGCACACGTGTGAAAGAGCCCATAGGCAAGGCCCGTTGGCCGCTA
GACACTGATGCAGACATGGTCGTATCTGTGGGCACAGCCAGTAAGGAATTAAAGGACTTCAAGGTGAGGGTCTCCTACTTTGGGGAGCAGGAAGACCAAGCTCTGGG
CCGCAGCGTGCTTTACCTCACTGGCGTCGATATTTCCCTTGAGGTTGACACAGGCCGCACAGGCAAGGTGAAGAGGAGCCAAGGGGACAAGAAAACCTGGCGCTGGG
GCCCTGAGGGCTATGGGGCTATCTTGCTGGTGAACTGTGACCGGGACAATCACAGGTCCGCAGAGCCTGACCTCACCCACAGCTGGCTGATGTCGCTGGCTGACCTG
CAGGACATGTCCCCAATGCTGCTGAGCTGCAATGGCCCCGACAAGCTCTTCGACAGCCACAAGCTTGTCTTGAACGTGCCCTTTTCTGATTCCAAAAGAGTGAGGGT
CTTCTGTGCCAGGGGTGGGAATTCTCTCTCGGACTACAAACAGGTGCTGGGGCCCCAGTGTCTGTCCTATGAAGTTGAGCGACAGCCAGGGGAGCAGGAGATCAAGT
TCTATGTGGAGGGGCTGACCTTCCCCGATGCCGATTTCCTAGGGCTGGTTTCCCTCAGTGTCAGCCTGGTGGACCCGGGGACCCTGCCCGAGGTGACCCTCTTCACA
GACACTGTGGGCTTCCGCATGGCCCCCTGGATCATGACGCCCAACACTCAGCCTCCTGAGGAGCTGTATGTGTGCAGAGTGATGGACACTCATGGCTCCAATGAGAA
ATTCCTGGAGGACATGTCTTATCTGACATTGAAAGCCAACTGCAAGCTGACCATCTGCCCTCAAGTTGAAAATCGAAATGACCGCTGGATCCAGGACGAGATGGAGT
TTGGCTACATCGAGGCCCCTCACAAATCCTTCCCCGTGGTCTTTGACTCCCCCCGGAACAGGGGCCTGAAAGATTTCCCCTATAAGAGGATCCTGGGTCCTGACTTT
GGATATGTTACCCGGGAGATCCCGCTCCCTGGTCCCTCCAGCCTTGACTCCTTCGGCAACCTGGACGTCAGCCCGCCCGTCACGGTGGCGGCACGGAATACCCCCT
GGGCCGGATCCTCATCGGGAGCAGCTTCCCCAAGTCCGGTGGGCGGCAGATGGCCAGGGCAGTGCGGAACTTCCTGAAGGCACAGCAGGTGCAGGCACCCGTGGAGC
TCTACTCGGACTGGCTCTCTGTGGGCCATGTGGACGAGTTTCTGACCTTTGTGCCTACCTCTGACCAAAAGGGCTTCCGGCTGCTCCTGGCTAGCCCCAGCGCTTGC
CTCAAACTCTTCCAAGAGAAGAAAGAAGAGGGTTATGGGGAGGCAGCCCAGTTTGATGGGTTAAAACACCAGGCAAAAAGAAGCATTAATGAGATGCTGGCAGACAG
ACACCTCCAGAGAGACAATCTTCATGCACAGAAATGCATTGACTGGAACCGTAATGTGCTGAAGCGGGAGCTGGGCCTGGCAGAGAGTGACATCGTGGACATTCCCC
AGCTCTTCTTCCTGAAAAACTTCTACGCGGAAGCCTTCTTCCCAGACATGGTTAACATGGTGGTCTTAGGCAAGTACCTGGGCATCCCCAAGCCCTACGGGCCCATC
ATCAATGGCCGCTGCTGCCTGGAGGAGAAGGTGCAGTCCCTGCTGGAGCCTCTGGGCCTGCACTGCATCTTCATTGATGACTACTTGTCCTACCACGAGCTGCAGGG
GGAGATCCACTGTGGCACCAACGTGCGCAGGAAGCCCTTTCCCTTCAAATGGTGGAACATGGTGCCTTGAAATTCAGGGTACCCAATAAGCTTACAATTGGTGGTAC
ATAGAATGGTTAACTTTAACAAGGAGAAAAACATGAAGGATAACACCGTGCCACTGAAATTGATTGCCCTGTTAGCGAACGGTGAATTTCACTCTGGCGAGCAGTTG
GGTGAAACGCTGGGAATGAGCCGGGCGGCTATTAATAAACACATTCAGACACTGCGTGACTGGGGCGTTGATGTCTTTACCGTTCCGGGTAAAGGATACAGCCTGCC
TGAGCCCATCCAGTTACTTAATGCTGAACAGATATTGGGTCAGCTGGATGGCGGTAGTGTAGCCGTGCTGCCAGTTATTGACTCCACGAATCAGTACCTTCTT

His Tag
Avi Tag
hPADI1

hPADI2 plasmid

```
GTCATCAAAATCACTCGCATCAACCAAACCGTTATTCATTCGTGATTGCGCCTGAGCGAGACGAAATACGCGATCGCTGTTAAAAGGACAATTACAAACAGGAATCG
AATGCAACCGGCGCAGGAACACTGCCAGCGCATCAACAATATTTTCACCTGAATCAGGATATTCTTCTAATACCTGGAATGCTGTTTTCCCGGGGATCGCAGTGGTG
AGTAACCATGCATCATCAGGAGTACGGATAAAATGCTTGATGGTCGGAAGAGGCATAAATTCCGTCAGCCAGTTTAGTCTGACCATCTCATCTGTAACATCATTGGC
AACGCTACCTTTGCCATGTTTCAGAAACAACTCTGGCGCATCGGGCTTCCCATACAATCGATAGATTGTCGCACCTGATTGCCCGACATTATCGCGAGCCCATTTAT
ACCCATATAAATCAGCATCCATGTTGGAATTTAATCGCGGCCTAGAGCAAGACGTTTCCCGTTGAATATGGCTCATAACACCCCTTGTATTACTGTTTATGTAAGCA
GACAGTTTTATTGTTCATGACCAAAATCCCTTAACGTGAGTTTTCGTTCCACTGAGCGTCAGACCCCGTAGAAAAGATCAAAGGATCTTCTTGAGATCCTTTTTTTC
TGCGCGTAATCTGCTGCTTGCAAACAAAAAAACCACCGCTACCAGCGGTGGTTTGTTTGCCGGATCAAGAGCTACCAACTCTTTTTCCGAAGGTAACTGGCTTCAGC
AGAGCGCAGATACCAAATACTGTCCTTCTAGTGTAGCCGTAGTTAGGCCACCACTTCAAGAACTCTGTAGCACCGCCTACATACCTCGCTCTGCTAATCCTGTTACC
AGTGGCTGCTGCCAGTGGCGATAAGTCGTGTCTTACCGGGTTGGACTCAAGACGATAGTTACCGGATAAGGCGCAGCGGTCGGGCTGAACGGGGGGTTCGTGCACAC
AGCCCAGCTTGGAGCGAACGACCTACACCGAACTGAGATACCTACAGCGTGAGCTATGAGAAAGCGCCACGCTTCCCGAAGGGAGAAAGGCGGACAGGTATCCGGTA
AGCGGCAGGGTCGGAACAGGAGAGCGCACGAGGGAGCTTCCAGGGGGAAACGCCTGGTATCTTTATAGTCCTGTCGGGTTTCGCCACCTCTGACTTGAGCGTCGATT
TTTGTGATGCTCGTCAGGGGGGCGGAGCCTATGGAAAAACGCCAGCAACGCGGCCTTTTTACGGTTCCTGGCCTTTTGCTGGCCTTTTGCTCACATGTTCTTTCCTG
CGTTATCCCCTGATTCTGTGGATAACCGTATTACCGCCTTTGAGTGAGCTGATACCGCTCGCCGCAGCCGAACGACCGAGCGCAGCGAGTCAGTGAGCGAGGAAGCG
GAAGAGCGCCTGATGCGGTATTTTCTCCTTACGCATCTGTGCGGTATTTCACACCGCAATGGTGCACTCTCAGTACAATCTGCTCTGATGCCGCATAGTTAAGCCAG
TATACACTCCGCTATCGCTACGTGACTGGGTCATGGCTGCGCCCCGACACCCGCCAACACCCGCTGACGCGCCCTGACGGGCTTGTCTGCTCCCGGCATCCGCTTAC
AGACAAGCTGTGACCGTCTCCGGGAGCTGCATGTGTCAGAGGTTTTCACCGTCATCACCGAAACGCGCGAGGCAGCTGCGGTAAAGCTCATCAGCGTGGTCGTGAAG
CGATTCACAGATGTCTGCCTGTTCATCCGCGTCCAGCTCGTTGAGTTTCTCCAGAAGCGTTAATGTCTGGCTTCTGATAAAGCGGGCCATGTTAAGGGCGGTTTTTT
CCTGTTTGGTCACTGATGCCTCCGTGTAAGGGGGATTTCTGTTCATGGGGGTAATGATACCGATGAAACGAGAGAGGATGCTCACGATACGGGTTACTGATGATGAA
CATGCCCGGTTACTGGAACGTTGTGAGGGTAAACAACTGGCGGTATGGATGCGGCGGGACCAGAGAAAAATCACTCAGGGTCAATGCCAGCGCTTCGTTAATACAGA
TGTAGGTGTTCCACAGGGTAGCCAGCAGCATCCTGCGATGCAGATCCGGAACATAATGGTGCAGGGCGCTGACTTCCGCGTTTCCAGACTTTACGAAACACGGAAAC
CGAAGACCATTCATGTTGTTGCTCAGGTCGCAGACGTTTTGCAGCAGCAGTCGCTTCACGTTCGCTCGCGTATCGGTGATTCATTCTGCTAACCAGTAAGGCAACCC
CGCCAGCCTAGCCGGGTCCTCAACGACAGGAGCACGATCATGCGCACCCGTGGGGCCGCCATGCCGGCGATAATGGCCTGCTTCTCGCCGAAACGTTTGGTGGCGGG
ACCAGTGACGAAGGCTTGAGCGAGGGCGTGCAAGATTCCGAATACCGCAAGCGACAGGCCGATCATCGTCGCGCTCCAGCGAAAGCGGTCCTCGCCGAAAATGACCC
AGAGCGCTGCCGGCACCTGTCCTACGAGTTGCATGATAAAGAAGACAGTCATAAGTGCGGCGACGATAGTCATGCCCCGCGCCCACCGGAAGGAGCTGACTGGGTTG
AAGGCTCTCAAGGGCATCGGTCGAGATCCCGGTGCCTAATGAGTGAGCTAACTTACATTAATTGCGTTGCGCTCACTGCCCGCTTTCCAGTCGGGAAACCTGTCGTG
CCAGCTGCATTAATGAATCGGCCAACGCGCGGGGAGAGGCGGTTTGCGTATTGGGCGCCAGGGTGGTTTTTCTTTTCACCAGTGAGACGGGCAACAGCTGATTGCCC
TTCACCGCCTGGCCCTGAGAGAGTTGCAGCAAGCGGTCCACGCTGGTTTGCCCCAGCAGGCGAAAATCCTGTTTGATGGTGGTTAACGGCGGGATATAACATGAGCT
GTCTTCGGTATCGTCGTATCCCACTACCGAGATATCCGCACCAACGCGCAGCCCGGACTCGGTAATGGCGCGCATTGCGCCCAGCGCCATCTGATCGTTGGCAACCA
GCATCGCAGTGGGAACGATGCCCTCATTCAGCATTTGCATGGTTTGTTGAAAACCGGACATGGCACTCCAGTCGCCTTCCCGTTCCGCTATCGGCTGAATTTGATTG
CGAGTGAGATATTTATGCCAGCCAGCCAGACGCAGACGCGCCGAGACAGAACTTAATGGGCCCGCTAACAGCGCGATTTGCTGGTGACCCAATGCGACCAGATGCTC
CACGCCCAGTCGCGTACCGTCTTCATGGGAGAAAATAATACTGTTGATGGGTGTCTGGTCAGAGACATCAAGAAATAACGCCGGAACATTAGTGCAGGCAGCTTCCA
CAGCAATGGCATCCTGGTCATCCAGCGGATAGTTAATGATCAGCCCACTGACGCGTTGCGCGAGAAGATTGTGCACCGCCGCTTTACAGGCTTCGACGCCGCTTCGT
TCTACCATCGACACCACCACGCTGGCACCCAGTTGATCGGCGCGAGATTTAATCGCCGCGACAATTTGCGACGGCGCGTGCAGGGCCAGACTGGAGGTGGCAACGCC
AATCAGCAACGACTGTTTGCCCGCCAGTTGTTGTGCCACGCGGTTGGGAATGTAATTCAGCTCCGCCATCGCCGCTTCCACTTTTTCCCGCGTTTTCGCAGAAACGT
GGCTGGCCTGGTTCACCACGCGGGAAACGGTCTGATAAGAGACACCGGCATACTCTGCGACATCGTATAACGTTACTGGTTTCACATTCACCACCCTGAATTGACTC
TCTTCCGGGCGCTATCATGCCATACCGCGAAAGGTTTTGCGCCATTCGATGGTGTCCGGGATCTCGACGCTCTCCCTTATGCGACTCCTGCATTAGGAAGCAGCCCA
GTAGTAGGTTGAGGCCGTTGAGCACCGCCGCCGCAAGGAATGGTGCATGCAAGGAGATGGCGCCCAACAGTCCCCCGGCCACGGGGCCTGCCACCATACCCACGCCG
AAACAAGCGCTCATGAGCCCGAAGTGGCGAGCCCGATCTTCCCCATCGGTGATGTCGGCGATATAGGCGCCAGCAACCGCACCTGTGGCGCCGGTGATGCCGGCCAC
GATGCGTCCGGCGTAGAGGATCGAGATCTCGATCCCGCGAAATTAATACGACTCACTATAGGGGAATTGTGAGCGGATAACAATTCCCCTCTAGAAATAATTTTGTT
TAACTTTAAGAAGGAGATATACCATGAGCGGCCTGAACGATATTTTTGAAGCGCAGAAAATTGAATGGCATGAAGGCAGCGCTGGAGGTTCAGGTCATCACCACCAT
CATCACGGTAGTGGTGGAATGCTGCGCGAGCGGACCGTGCGGCTGCAGTACGGGAGCCGCGTGGAGGCGGTGTACGTGCTGGGCACCTACCTCTGGACCGATGTCTA
CAGCGCGGCCCCAGCCGGGGCCCAAACCTTCAGCCTGAAGCACTCGGAACACGTGTGGGTGGAGGTGGTGCGTGATGGGGAGGCTGAGGAGGTGGCCACCAATGGCA
AGCAGCGCTGGCTTCTCTCGCCCAGCACCACCCTGCGGGTCACCATGAGCCAGGCGAGCACCGAGGCCAGCAGTGACAAGGTCACCGTCAACTACTATGACGAGGAA
GGGAGCATTCCCATCGACCAGGCGGGGCTCTTCCTCACAGCCATTGAGATCTCCCTGGATGTGGACGCAGACCGGGATGGTGTGGTGGAGAAGAACAACCCAAAGAA
GGCATCCTGGACCTGGGGCCCCGAGGGCCAGGGGGCCATCCTGCTGGTGAACTGTGACCGAGAGACACCCTGGTTGCCCAAGGAGGACTGCCGTGATGAGAAGGTCT
ACAGCAAGGAAGATCTCAAGGACATGTCCCAGATGATCCTGCGGACCAAAGGCCCCGACCGCCTCCCCGCCGGATACGAGATAGTTCTGTACATTTCCATGTCAGAC
TCAGACAAAGTGGGCGTGTTCTACGTGGAGAACCCGTTCTTCGGCCAACGCTATATCCACATCCTGGGCCGGCGGAAGCTCTACCATGTGGTCAAGTACACGGGTGG
CTCCGCGGAGCTGCTGTTCTTCGTGGAAGGCCTCTGTTTCCCCGACGAGGGCTTCTCAGGCCTGGTCTCCATCCATGTCAGCCTGCTGGAGTACATGGCCCAGGACA
TTCCCCTGACTCCCATCTTCACGGACACCGTGATATTCCGGATTGCTCCGTGGATCATGACCCCCAACATCCTGCCTCCCGTGTCGGTGTTTGTGTGCTGCATGAAG
GATAATTACCTGTTCCTGAAAGAGGTGAAGAACCTTGTGGAGAAAACCAACTGTGAGCTGAAGGTCTGCTTCCAGTACCTAAACCGAGGCGATCGCTGGATCCAGGA
TGAAATTGAGTTTGGCTACATCGAGGCCCCCCATAAAGGCTTCCCCGTGGTGCTGGACTCTCCCCGAGATGGAAACCTAAAGGACTTCCCTGTGAAGGAGCTCCTGG
GCCCAGATTTTGGCTACGTGACCCGGGAGCCCCTCTTTGAGTCTGTCACCAGCCTTGACTCATTTGGAAACCTGGAGGTCAGTCCCCCAGTGACCGTGAATGGCAAG
ACATACCCGCTTGGCCGCATCCTCATCGGGAGCAGCTTTCCTCTGTCTGGTGGTCGGAGGATGACCAAGGTGGTGCGTGACTTCCTGAAGGCCCAGCAGGTGCAGGC
ACCCGTGGAGCTCTACTCAGACTGGCTGACTGTGGGCCACGTGGATGAGTTCATGTCCTTTGTCCCCATCCCCGGCACAAAGAAATTCCTGCTACTCATGGCCAGCA
CCTCGGCCTGCTACAAGCTCTTCCGAGAGAAGCAGAAGGACGGCCATGGAGAGGCCATCATGTTCAAAGGCTTGGGTGGGATGAGCAGCAAGCGAATCACCATCAAC
AAGATTCTGTCCAACGAGAGCCTTGTGCAGGAGAACCTGTACTTCCAGCGCTGCCTGGACTGGAACCGTGACATCCTCAAGAAGGAGCTGGGACTGACAGAGCAGGA
CATCATTGACCTGCCCGCTCTGTTCAAGATGGACGAGGACCACCGTGCCAGAGCCTTCTTCCCAAACATGGTGAACATGATCGTGCTGGACAAGGACCTGGGCATCC
CCAAGCCATTCGGGCCACAGGTTGAGGAGGAATGCTGCCTGGAGATGCACGTGCGTGGCCTCCTGGAGCCCCTGGGCCTCGAATGCACCTTCATCGACGACATTTCT
GCCTACCACAAATTTCTGGGGGAAGTCCACTGTGGCACCAACGTCCGCAGGAAGCCCTTCACCTTCAAGTGGTGGCACATGGTGCCCTAAGGTGGAAGTGGTCATCA
CCACCATCATCACTGATGACGAAGCTTGCGGCCGCACTCGAGCACCACCACCACCACCACTGAGATCCGGCTGCTAACAAAGCCCGAAAGGAAGCTGAGTTGGCTGC
TGCCACCGCTGAGCAATAACTAGCATAACCCCTTGGGGCCTCTAAACGGGTCTTGAGGGGTTTTTTGCTGAAAGGAGGAACTATATCCGGATTGGCGAATGGGACGC
GCCCTGTAGCGGCGCATTAAGCGCGGCGGGTGTGGTGGTTACGCGCAGCGTGACCGCTACACTTGCCAGCGCCCTAGCGCCCGCTCCTTTCGCTTTCTTCCCTTCCT
TTCTCGCCACGTTCGCCGGCTTTCCCCGTCAAGCTCTAAATCGGGGGCTCCCTTTAGGGTTCCGATTTAGTGCTTTACGGCACCTCGACCCCAAAAAACTTGATTAG
GGTGATGGTTCACGTAGTGGGCCATCGCCCTGATAGACGGTTTTTCGCCCTTTGACGTTGGAGTCCACGTTCTTTAATAGTGGACTCTTGTTCCAAACTGGAACAAC
ACTCAACCCTATCTCGGTCTATTCTTTTGATTTATAAGGGATTTTGCCGATTTCGGCCTATTGGTTAAAAAATGAGCTGATTTAACAAAAATTTAACGCGAATTTTA
ACAAAATATTAACGCTTACAATTTAGGTGGCACTTTTCGGGGAAATGTGCGCGGAACCCCTATTTGTTTATTTTTCTAAATACATTCAAATATGTATCCGCTCATGA
ATTAATTCTTAGAAAAACTCATCGAGCATCAAATGAAACTGCAATTTATTCATATCAGGATTATCAATACCATATTTTTGAAAAAGCCGTTTCTGTAATGAAGGAGA
AAACTCACCGAGGCAGTTCCATAGGATGGCAAGATCCTGGTATCGGTCTGCGATTCCGACTCGTCCAACATCAATACAACCTATTAATTTCCCCTCGTCAAAAATAA
GGTTATCAAGTGAGAAATCACCATGAGTGACGACTGAATCCGGTGAGAATGGCAAAAGTTTATGCATTTCTTTCCAGACTTGTTCAACAGGCCAGCCATTACGCTC
```

His Tag
Avi Tag
hPADI2

hPADI3 plasmid

```
CCTTCTTGATCGTATCGGAGAGCTTAAATCGGGCGATGCCTGTGTTGCAGAATACCAGCAGGCTGGCCGTGGTCGCCGGGGGCGGAAATGGTTTTCGCCTTTTGGCG
CAAACTTATATTTGTCGATGTTCTGGCGTCTGGAACAAGGCCCGGCGGCGGCGATTGGTTTAAGTCTGGTTATCGGTATCGTGATGGCGGAAGTATTACGCAAGCTG
GGAGCAGATAAAGTTCGTGTCAAATGGCCTAATGACCTCTATCTGCAGGATCGCAAGCTGGCAGGCATTCTTGTGGAGCTGACTGGCAAAACTGGCGATGCGGCGCA
AATAGTCATTGGAGCCGGGATCAACATGGCAATGCGCCGTGTTGAAGAGAGTGTCGTTAATCAGGGGTGGATCACGCTGCAGGAAGCGGGGATCAATCTCGATCGTA
ATACGTTGGCGGCCATGCTAATACGTGAATTACGTGCTGCGTTGGAACTCTTCGAACAAGAAGGATTGGCACCTTATCTGTCGCGCTGGGAAAAGCTGGATAATTTT
ATTAATCGCCCAGTGAAACTTATCATTGGTGATAAAGAAATATTTGGCATTTCACGCGGAATAGACAAACAGGGGGCTTTATTACTTGAGCAGGATGGAATAATAAA
ACCCTGGATGGGCGGTGAAATATCCCTGCGTAGTGCAGAAAAAATAAGCGGCCGCAGCTTAATTAGCTGAGCTTGGACTCCTGTTGATAGATCCAGTAATGACCTCAG
AACTCCATCTGGATTTGTTCAGAACGCTCGGTTGCCGCCGGGCGTTTTTTATTGGTGAGAATCCAAGCTAGCTTGGCGAGATTTTCAGGAGCTAAGGAAGCTAAAAT
GGAGAAAAAAATCACTGGATATACCACCGTTGATATATCCCAATGGCATCGTAAAGAACATTTTGAGGCATTTCAGTCAGTTGCTCAATGTACCTATAACCAGACCG
TTCAGCTGGATATTACGGCCTTTTTAAAGACCGTAAAGAAAAATAAGCACAAGTTTTATCCGGCCTTTATTCACATTCTTGCCCGCCTGATGAATGCTCATCCGGAA
TTTCGTATGGCAATGAAAGACGGTGAGCTGGTGATATGGGATAGTGTTCACCCTTGTTACACCGTTTTCCATGAGCAAACTGAAACGTTTTCATCGCTCTGGAGTGA
ATACCACGACGATTTCCGGCAGTTTCTACACATATATTCGCAAGATGTGGCGTGTTACGGTGAAAACCTGGCCTATTTCCCTAAAGGGTTTATTGAGAATATGTTTT
TCGTCTCAGCCAATCCCTGGGTGAGTTTCACCAGTTTTGATTTAAACGTGGCCAATATGGACAACTTCTTCGCCCCCGTTTTCACCATGGGCAAATATTATACGCAA
GGCGACAAGGTGCTGATGCCGCTGGCGATTCAGGTTCATCATGCCGTTTGTGATGGCTTCCATGTCGGCAGAATGCTTAATGAATTACAACAGTACTGCGATGAGTG
GCAGGGCGGGGCGTAATTTTTTTAAGGCAGTTATTGGTGCCCTTAAACGCCTGGGGTAATGACTCTCTAGCTTGAGGCATCAAATAAAACGAAAGGCTCAGTCGAAA
GACTGGGCCTTTCGTTTTATCTGTTGTTTGTCGGTGAACGCTCTCCTGAGTAGGACAAATCCGCCCTCTAGATTACGTGCAGTCGATGATAAGCTGTCAAACATGAG
AATTGTGCCTAATGAGTGAGCTAACTTACATTAATTGCGTTGCGCTCACTGCCCGCTTTCCAGTCGGGAAACCTGTCGTGCCAGCTGCATTAATGAATCGGCCAACG
CGCGGGGAGAGGCGGTTTGCGTATTGGGCGCCAGGGTGGTTTTTCTTTTCACCAGTGAGACGGGCAACAGCTGATTGCCCTTCACCGCCTGGCCCTGAGAGAGTTGC
AGCAAGCGGTCCACGCTGGTTTGCCCCAGCAGGCGAAAATCCTGTTTGATGGTGGTTAACGGCGGGATATAACATGAGCTGTCTTCGGTATCGTCGTATCCCACTAC
CGAGATATCCGCACCAACGCGCAGCCCGGACTCGGTAATGGCGCGCATTGCGCCCAGCGCCATCTGATCGTTGGCAACCAGCATCGCAGTGGGAACGATGCCCTCAT
TCAGCATTTGCATGGTTTGTTGAAAACCGGACATGGCACTCCAGTCGCCTTCCCGTTCCGCTATCGGCTGAATTTGATTGCGAGTGAGATATTTATGCCAGCCAGCC
AGACGCAGACGCGCCGAGACAGAACTTAATGGGCCCGCTAACAGCGCGATTTGCTGGTGACCCAATGCGACCAGATGCTCCACGCCCAGTCGCGTACCGTCTTCATG
GGAGAAAATAATACTGTTGATGGGTGTCTGGTCAGAGACATCAAGAAATAACGCCGGAACATTAGTGCAGGCAGCTTCCACAGCAATGGCATCCTGGTCATCCAGCG
GATAGTTAATGATCAGCCCACTGACGCGTTGCGCGAGAAGATTGTGCACCGCCGCTTTACAGGCTTCGACGCCGCTTCGTTCTACCATCGACACCACCACGCTGGCA
CCCAGTTGATCGGCGCGAGATTTAATCGCCGCGACAATTTGCGACGGCGCGTGCAGGGCCAGACTGGAGGTGGCAACGCCAATCAGCAACGACTGTTTGCCCGCCAG
TTGTTGTGCCACGCGGTTGGGAATGTAATTCAGCTCCGCCATCGCCGCTTCCACTTTTTCCCGCGTTTTCGCAGAAACGTGGCTGGCCTGGTTCACCACGCGGGAAA
CGGTCTGATAAGAGACACCGGCATACTCTGCGACATCGTATAACGTTACTGGTTTCACATTCACCACCCTGAATTGACTCTCTTCCGGGCGCTATCATGCCATACCG
CGAAAGGTTTTGCACCATTCGATGGTGTCGGAATTTCGGGCAGCGTTGGGTCCTGGCCACGGGTGCGCATGATCTAGAGCTGCCTCGCGCGTTTCGGTGATGACGGT
GAAAACCTCTGACACATGCAGCTCCCGGAGACGGTCACAGCTTGTCTGTAAGCGGATGCCGGGAGCAGACAAGCCCGTCAGGGCGCGTCAGCGGGTGTTGGCGGGTG
TCGGGGCGCAGCCATGACCCAGTCACGTAGCGATAGCGGAGTGTATACTGGCTTAACTATGCGGCATCAGAGCAGATTGTACTGAGAGTGCACCATATGCGGTGTGA
AATACCGCACAGATGCGTAAGGAGAAAATACCGCATCAGGCGCTCTTCCGCTTCCTCGCTCACTGACTCGCTGCGCTCGGTCGTTCGGCTGCGGCGAGCGGTATCAG
CTCACTCAAAGGCGGTAATACGGTTATCCACAGAATCAGGGGATAACGCAGGAAAGAACATGTGAGCAAAAGGCCAGCAAAAGGCCAGGAACCGTAAAAAGGCCGCG
TTGCTGGCGTTTTTCCATAGGCTCCGCCCCCCTGACGAGCATCACAAAAATCGACGCTCAAGTCAGAGGTGGCGAAACCCGACAGGACTATAAAGATACCAGGCGTT
TCCCCCTGGAAGCTCCCTCGTGCGCTCTCCTGTTCCGACCCTGCCGCTTACCGGATACCTGTCCGCCTTTCTCCCTTCGGGAAGCGTGGCGCTTTCTCATAGCTCAC
GCTGTAGGTATCTCAGTTCGGTGTAGGTCGTTCGCTCCAAGCTGGGCTGTGTGCACGAACCCCCCGTTCAGCCCGACCGCTGCGCCTTATCCGGTAACTATCGTCTT
GAGTCCAACCCGGTAAGACACGACTTATCGCCACTGGCAGCAGCCACTGGTAACAGGATTAGCAGAGCGAGGTATGTAGGCGGTGCTACAGAGTTCTTGAAGTGGTG
GCCTAACTACGGCTACACTAGAAGGACAGTATTTGGTATCTGCGCTCTGCTGAAGCCAGTTACCTTCGGAAAAAGAGTTGGTAGCTCTTGATCCGGCAAACAAACCA
CCGCTGGTAGCGGTGGTTTTTTTGTTTGCAAGCAGCAGATTACGCGCAGAAAAAAAGGATCTCAAGAAGATCCTTTGATCTTTTCTACGGGGTCTGACGCTCAGTGG
AACGAAAACTCACGTTAAGGGATTTTGGTCATGAGATTATCAAAAAGGATCTTCACCTAGATCCTTTTAAATTAAAAATGAAGTTTTAAATCAATCTAAAGTATATA
TGAGTAAACTTGGTCTGACAGTTACCAATGCTTAATCAGTGAGGCACCTATCTCAGCGATCTGTCTATTTCGTTCATCCATAGTTGCCTGACTCCCCGTCGTGTAGA
TAACTACGATACGGGAGGGCTTACCATCTGGCCCCAGTGCTGCAATGATACCGCGAGACCCACGCTCACCGGCTCCAGATTTATCAGCAATAAACCAGCCAGCCGGA
AGGGCCGAGCGCAGAAGTGGTCCTGCAACTTTATCCGCCTCCATCCAGTCTATTAATTGTTGCCGGGAAGCTAGAGTAAGTAGTTCGCCAGTTAATAGTTTGCGCAA
CGTTGTTGCCATTGCTACAGGCATCGTGGTGTCACGCTCGTCGTTTGGTATGGCTTCATTCAGCTCCGGTTCCCAACGATCAAGGCGAGTTACATGATCCCCCATGT
TGTGCAAAAAAGCGGTTAGCTCCTTCGGTCCTCCGATCGTTGTCAGAAGTAAGTTGGCCGCAGTGTTATCACTCATGGTTATGGCAGCACTGCATAATTCTCTTACT
GTCATGCCATCCGTAAGATGCTTTTCTGTGACTGGTGAGTACTCAACCAAGTCATTCTGAGAATAGTGTATGCGGCGACCGAGTTGCTCTTGCCCGGCGTCAATACG
GGATAATACCGCGCCACATAGCAGAACTTTAAAAGTGCTCATCATTGGAAAACGTTCTTCGGGGCGAAAACTCTCAAGGATCTTACCGCTGTTGAGATCCAGTTCGA
TGTAACCCACTCGTGCACCCAACTGATCTTCAGCATCTTTTACTTTCACCAGCGTTTCTGGGTGAGCAAAAACAGGAAGGCAAAATGCCGCAAAAAAGGGAATAAGG
GCGACACGGAAATGTTGAATACTCATACTCTTCCTTTTTCAATATTATTGAAGCATTTATCAGGGTTATTGTCTCATGAGCGGATACATATTTGAATGTATTTAGAA
AAATAAACAAATAGGGGTTCCGCGCACATTTCCCCGAAAAGTGCCACCTGACGTCTAAGAAACCATTATTATCATGACATTAACCTATAAAAATAGGCGTATCACGA
GGCCCTTTCGTCTTCACCTCGATTCGTCTTCACCTCGAGAAATCATAAAAAATTTATTTGCTTTGTGAGCGGATAACAATTATAATAGATTCAATTGTGAGCGGATA
ACAATTTCACACAGAATTTATTAAAGAGGAGAAATTACATATGTCCGGCCTGAACGACATCTTCGAGGCTCAGAAAATCGAATGGCACGAAGGTCACCATCATCACC
ACCACGGATCCATGTCGCTGCAGAGAATCGTGCGTGTGTCCCTGGAGCATCCCACCAGCGCGGTGTGTGTGGCTGGCGTGGAGACCCTCGTGGACATTTATGGGTCA
GTGCCTGAGGGCACAGAAATGTTTGAGGTCTATGGGACGCCTGGCGTGGACATCTACATCTCTCCCAACATGGAGAGGGGCCGGGAGCGTGCAGACACCAGGCGGTG
GCGCTTTGACGCGACTTTGGAGATCATCGTGGTCATGAACTCCCCCAGCAATGACCTCAACGACAGCCATGTTCAGATTTCCTACCACTCCAGCCATGAGCCTCTGC
CCCTGGCCTATGCGGTGCTCTACCTCACCTGTGTTGACATCTCTCTGGATTGCGACCTGAACTGTGAGGGAAGGCAGGACAGGAACTTTGTAGACAAGCGGCAGTGG
GTCTGGGGGCCCAGTGGGTATGGCGGCATCTTGCTGGTGAACTGTGACCGTGATGATCCGAGCTGTGATGTCCAGGACAATTGTGACCAGCACGTGCACTGCCTGCA
AGACCTGGAAGACATGTCTGTCATGGTCCTGCGGACGCAGGGCCCTGCAGCCCTCTTTGATGACCACAAACTTGTCCTCCATACCTCCAGCTATGATGCCAAACGGG
CACAGGTCTTCCACATCTGCGGTCCTGAGGATGTGTGTGAGGCCTATAGGCATGTGCTGGGCCAAGATAAGGTGTCCTATGAGGTACCCCGCTTGCATGGGGATGAG
GAGCGCTTCTTCGTGGAAGGCCTGTCCTTCCCTGATGCCGGCTTCACAGGACTCATCTCCTTCCATGTCACTCTGCTGGACGACTCCAACGAGGATTTCTCGGCATC
CCCTATCTTCACTGACACTGTGGTGTTCCGAGTGGCACCCTGGATCATGACGCCCAGCACTCTGCCACCCCTAGAGGTGTATGTGTGCCGTGTGAGGAACAACACGT
GTTTTGTGGATGCGGTGGCAGAGCTGGCCAGGAAGGCCGGCTGCAAGCTGACCATCTGCCCACAGGCCGAGAACCGCAACGACCGCTGGATCCAGGATGAGATGGAG
CTGGGCTACGTTCAGGCGCCGCACAAGACCCTCCCGGTGGTCTTTGACTCCCCAAGGAATGGGGAACTGCAGGATTTCCCTTACAAAAGAATCCTGGGTCCAGATTT
TGGTTACGTGACTCGGGAACCACGCGACAGGTCTGTGAGTGGCCTGGACTCCTTTGGGAACCTGGAGGTCAGCCCTCCAGTGGTGGCCAATGGAAAGAGTACCCCC
TGGGGAGGATCCTCATTGGGGGCAACCTGCCTGGGTCAAGTGGCCGCAGGGTCACCCAGGTGGTGCGGGACTTCCTCCATGCCCAGAAGGTGCAGCCCCCCGTGGAG
CTCTTTGTGGACTGGTTGGCCGTGGGCCATGTGGATGAGTTTCTGAGCTTTGTCCCTGCCCCCGATGGGAAGGGCTTCCGGATGCTCCTGGCCAGCCCTGGGGCCTG
CTTCAAGCTCTTCCAGGAAAAGCAGAAGTGTGGCCACGGGAGGGCCCTCCTGTTCCAGGGGGTTGTTGATGATGAGCAGGTCAAGACCATCTCCATCAACCAGGTGC
TCTCCAATAAAGACCTCATCAACTACAATAAGTTTGTGCAGAGCTGCATCGACTGGAACCGTGAGGTGCTGAAGCGGGAGCTGGGCCTGGCAGAGTGTGACATCATT
GACATCCCACAGCTCTTCAAGACCGAGAGGAAAAAAGCAACGGCCTTCTTCCCTGACTTGGTGAACATGCTGGTGCTGGGGAAGCACCTGGGCATCCCCAAGCCCTT
TGGGCCCATCATCAATGGCTGCTGCTGCCTGGAGGAGAAGGTGCGGTCCCTGCTGGAGCCTCTGGGCCTCCACTGCACCTTCATTGATGACTTCACTCCATACCACA
TGCTGCATGGGGAGGTGCACTGTGGCACCAATGTGTGCAGAAAGCCCTTCTCTTTCAAGTGGTGGAACATGGTGCCCTGAAATTCAGGGTACCCAATAAGCTTACAA
TTGGTGGTACATAGAATGGTTAACTTTAACAAGGAGAAAAACATGAAGGATAACACCGTGCCACTGAAATTGATTGCCCTGTTAGCGAACGGTGAATTTCACTCTGG
CGAGCAGTTGGGTGAAACGCTGGGAATGAGCCGGGCGGCTATTAATAAACACATTCAGACACTGCGTGACTGGGGCGTTGATGTCTTTACCGTTCCGGGTAAAGGAT
ACAGCCTGCCTGAGCCCATCCAGTTACTTAATGCTGAACAGATATTGGGTCAGCTGGATGGCGGTAGTGTAGCCGTGCTGCCAGTTATTGACTCCACGAATCAGTA
```

His Tag
Avi Tag
hPADI3

hPADI4

CCCGCTAACAGCGCGATTTGCTGGTGACCCAATGCGACCAGATGCTCCACGCCCAGTCGCGTACCGTCTTCATGGGAGAAAATAATACTGTTGATGGGTGTCTGGTC
AGAGACATCAAGAAATAACGCCGGAACATTAGTGCAGGCAGCTTCCACAGCAATGGCATCCTGGTCATCCAGCGGATAGTTAATGATCAGCCCACTGACGCGTTGCG
CGAGAAGATTGTGCACCGCCGCTTTACAGGCTTCGACGCCGCTTCGTTCTACCATCGACACCACCACGCTGGCACCCAGTTGATCGGCGCGAGATTTAATCGCCGCG
ACAATTTGCGACGGCGCGTGCAGGGCCAGACTGGAGGTGGCAACGCCAATCAGCAACGACTGTTTGCCCGCCAGTTGTTGTGCCACGCGGTTGGGAATGTAATTCAG
CTCCGCCATCGCCGCTTCCACTTTTTCCCGCGTTTTCGCAGAAACGTGGCTGGCCTGGTTCACCACGCGGGAAACGGTCTGATAAGAGACACCGGCATACTCTGCGA
CATCGTATAACGTTACTGGTTTCACATTCACCACCCTGAATTGACTCTCTTCCGGGCGCTATCATGCCATACCGCGAAAGGTTTTGCGCCATTCGATGGTGTCCGGG
ATCTCGACGCTCTCCCTTATGCGACTCCTGCATTAGGAAGCAGCCCAGTAGTAGGTTGAGGCCGTTGAGCACCGCCGCCGCAAGGAATGGTGCATGCAAGGAGATGG
CGCCCAACAGTCCCCCGGCCACGGGGCCTGCCACCATACCCACGCCGAAACAAGCGCTCATGAGCCCGAAGTGGCGAGCCCGATCTTCCCCATCGGTGATGTCGGCG
ATATAGGCGCCAGCAACCGCACCTGTGGCGCCGGTGATGCCGGCCACGATGCGTCCGGCGTAGAGGATCGAGATCTCGATCCCGCGAAATTAATACGACTCACTATA
GGGGAATTGTGAGCGGATAACAATTCCCCTCTAGAAATAATTTTGTTTAACTTTAAGAAGGAGATATACCATGAGCGGCCTGAACGATATTTTTGAAGCGCAGAAAA
TTGAATGGCATGAAGGCAGCGCTGGAGGTTCAGGTCATCACCACCATCATCACGGTAGTGGTGGAATGGCCCAGGGGACATTGATCCGTGTGACCCCAGAGCAGCCC
ACCCATGCCGTGTGTGTGCTGGGCACCTTGACTCAGCTTGACATCTGCAGCTCTGCCCCTGAGGACTGCACGTCCTTCAGCATCAACGCCTCCCCAGGGGTGGTCGT
GGATATTGCCCACAGCCCTCCAGCCAAGAAGAAATCCACAGGTTCCTCCACATGGCCCCTGGACCCTGGGGTAGAGGTGACCCTGACGATGAAAGCGGCCAGTGGTA
GCACAGGCGACCAGAAGGTTCAGATTTCATACTACGGACCCAAGACTCCACCAGTCAAAGCTCTACTCTACCTCACCGCGGTGGAAATCTCCCTGTGCGCAGACATC
ACCCGCACCGGCAAAGTGAAGCCAACCAGAGCTGTGAAAGATcAGAGGACCTGGACCTGGGGCCCTTGTGGACAGGGTGCCATCCTGCTGGTGAACTGTGACAGAGA
CAATCTCGAATCTTCTGCCATGGACTGCGAGGATGATGAAGTGCTTGACAGCGAAGACCTGCAGGACATGTCGCTGATGACCCTGAGCACGAAGACCCCCAAGGACT
TCTTCACAAACCATACACTGGTGCTCCACGTGGCCAGGTCTGAGATGGACAAAGTGAGGGTGTTTCAGGCCACACGGGGCAAACTGTCCTCCAAGTGCAGCGTAGTC
TTGGGTCCCAAGTGGCCCTCTCACTACCTGATGGTCCCCGGTGGAAAGCACAACATGGACTTCTACGTGGAGGCCCTCGCTTTCCCGGACACCGACTTCCCGGGGCT
CATTACCCTCACCATCTCCCTGCTGGACACGTCCAACCTGGAGCTCCCCGAGGCTGTGGTGTTCCAAGACAGCGTGGTCTTCCGCGTGGCGCCCTGGATcATGACCC
CCAACACCCAGCCCCCGCAGGAGGTGTACGCGTGCAGTATTTTTGAAAATGAGGACTTCCTGAAGTCAGTGACTACTCTGGCCATGAAAGCCAAGTGCAAGCTGACC
ATCTGCCCTGAGGAGGAGAACATGGATGACCAGTGGATGCAGGATGAAATGGAGATcGGCTACATCCAAGCCCCACACAAAACGCTGCCCGTGGTCTTCGACTCTCC
AAGGAACAGAGGCCTGAAGGAGTTTCCCATCAAACGAGTGATGGGTCCAGATTTTGGCTATGTAACTCGAGGGCCCCAAACAGGGGGTATCAGTGGACTGGACTCCT
TTGGGAACCTGGAAGTGAGCCCCCCAGTCACAGTCAGGGGCAAGGAATACCCGCTGGGCAGGATTCTCTTCGGGGACAGCTGTTATCCCAGCAATGACAGCCGGCAG
ATGCACCAGGCCCTACAGGACTTCCTCAGTGCCCAGCAGGTGCAGGCCCCTGTGAAGCTCTATTCTGACTGGCTGTCCGTGGGCCACGTGGACGAGTTCCTGAGCTT
TGTGCCAGCACCCGACAGGAAGGGCTTCCGGCTGCTCCTGGCCAGCCCCAGGTCCTGCTACAAACTGTTCCAGGAGCAGCAGAATGAGGGCCACGGGGAGGCCCTGC
TGTTCGAAGGGATCAAGAAAAAAAAACAGCAGAAAATAAAGAACATTCTGTCAAACAAGACATTGAGAGAACATAATTCATTTGTGGAGAGATGCATCGACTGGAAC
CGCGAGCTGCTGAAGCGGGAGCTGGGCCTGGCCGAGAGTGACATCATTGACATCCCGCAGCTCTTCAAGCTCAAAGAGTTCTCTAAGGCGGAAGCTTTTTTCCCCAA
CATGGTGAACATGCTGGTGCTAGGGAAGCACCTGGGCATCCCCAAGCCCTTCGGGCCCGTCATCAACGGCCGCTGCTGCCTGGAGGAGAAGGTGTGTTCCCTGCTGG
AGCCACTGGGCCTCCAGTGCACCTTCATCAACGACTTCTTCACCTACCACATCAGGCATGGGGAGGTGCACTGCGGCACCAACGTGCGCAGAAAGCCCTTCTCCTTC
AAGTGGTGGAACATGGTGCCCTGAGGTGGAAGTGGTCATCACCACCATCATCACTGATGACGAAGCTTGCGGCCGCACTCGAGCACCACCACCACCACCACTGAGAT
CCGGCTGCTAACAAAGCCCGAAAGGAAGCTGAGTTGGCTGCTGCCACCGCTGAGCAATAACTAGCATAACCCCTTGGGGCCTCTAAACGGGTCTTGAGGGGTTTTTT
GCTGAAAGGAGGAACTATATCCGGATTGGCGAATGGGACGCGCCCTGTAGCGGCGCATTAAGCGCGGCGGGTGTGGTGGTTACGCGCAGCGTGACCGCTACACTTGC
CAGCGCCCTAGCGCCCGCTCCTTTCGCTTTCTTCCCTTCCTTTCTCGCCACGTTCGCCGGCTTTCCCCGTCAAGCTCTAAATCGGGGGCTCCCTTTAGGGTTCCGAT
TTAGTGCTTTACGGCACCTCGACCCCAAAAAACTTGATTAGGGTGATGGTTCACGTAGTGGGCCATCGCCCTGATAGACGGTTTTTCGCCCTTTGACGTTGGAGTCC
ACGTTCTTTAATAGTGGACTCTTGTTCCAAACTGGAACAACACTCAACCCTATCTCGGTCTATTCTTTTGATTTATAAGGGATTTTGCCGATTTCGGCCTATTGGTT
AAAAAATGAGCTGATTTAACAAAAATTTAACGCGAATTTTAACAAAATATTAACGCTTACAATTTAGGTGGCACTTTTCGGGGAAATGTGCGCGGAACCCCTATTTG
TTTATTTTTCTAAATACATTCAAATATGTATCCGCTCATGAATTAATTCTTAGAAAAACTCATCGAGCATCAAATGAAACTGCAATTTATTCATATCAGGATTATCA
ATACCATATTTTTGAAAAAGCCGTTTCTGTAATGAAGGAGAAAACTCACCGAGGCAGTTCCATAGGATGGCAAGATCCTGGTATCGGTCTGCGATTCCGACTCGTCC
AACATCAATACAACCTATTAATTTCCCCTCGTCAAAAATAAGGTTATCAAGTGAGAAATCACCATGAGTGACGACTGAATCCGGTGAGAATGGCAAAAGTTTATGCA
TTTCTTTCCAGACTTGTTCAACAGGCCAGCCATTACGCTCGTCATCAAAATCACTCGCATCAACCAAACCGTTATTCATTCGTGATTGCGCCTGAGCGAGACGAAAT
ACGCGATCGCTGTTAAAAGGACAATTACAAACAGGAATCGAATGCAACCGGCGCAGGAACACTGCCAGCGCATCAACAATATTTTCACCTGAATCAGGATATTCTTC
TAATACCTGGAATGCTGTTTTCCCGGGGATCGCAGTGGTGAGTAACCATGCATCATCAGGAGTACGGATAAAATGCTTGATGGTCGGAAGAGGCATAAATTCCGTCA
GCCAGTTTAGTCTGACCATCTCATCTGTAACATCATTGGCAACGCTACCTTTGCCATGTTTCAGAAACAACTCTGGCGCATCGGGCTTCCCATACAATCGATAGATT
GTCGCACCTGATTGCCCGACATTATCGCGAGCCCATTTATACCCATATAAATCAGCATCCATGTTGGAATTTAATCGCGGCCTAGAGCAAGACGTTTCCCGTTGAAT
ATGGCTCATAACACCCCTTGTATTACTGTTTATGTAAGCAGACAGTTTTATTGTTCATGACCAAAATCCCTTAACGTGAGTTTTCGTTCCACTGAGCGTCAGACCCC
GTAGAAAAGATCAAAGGATCTTCTTGAGATCCTTTTTTTCTGCGCGTAATCTGCTGCTTGCAAACAAAAAAACCACCGCTACCAGCGGTGGTTTGTTTGCCGGATCA
AGAGCTACCAACTCTTTTTCCGAAGGTAACTGGCTTCAGCAGAGCGCAGATACCAAATACTGTCCTTCTAGTGTAGCCGTAGTTAGGCCACCACTTCAAGAACTCTG
TAGCACCGCCTACATACCTCGCTCTGCTAATCCTGTTACCAGTGGCTGCTGCCAGTGGCGATAAGTCGTGTCTTACCGGGTTGGACTCAAGACGATAGTTACCGGAT
AAGGCGCAGCGGTCGGGCTGAACGGGGGGTTCGTGCACACAGCCCAGCTTGGAGCGAACGACCTACACCGAACTGAGATACCTACAGCGTGAGCTATGAGAAAGCGC
CACGCTTCCCGAAGGGAGAAAGGCGGACAGGTATCCGGTAAGCGGCAGGGTCGGAACAGGAGAGCGCACGAGGGAGCTTCCAGGGGGAAACGCCTGGTATCTTTATA
GTCCTGTCGGGTTTCGCCACCTCTGACTTGAGCGTCGATTTTTGTGATGCTCGTCAGGGGGGCGGAGCCTATGGAAAAACGCCAGCAACGCGGCCTTTTTACGGTTC
CTGGCCTTTTGCTGGCCTTTTGCTCACATGTTCTTTCCTGCGTTATCCCCTGATTCTGTGGATAACCGTATTACCGCCTTTGAGTGAGCTGATACCGCTCGCCGCAG
CCGAACGACCGAGCGCAGCGAGTCAGTGAGCGAGGAAGCGGAAGAGCGCCTGATGCGGTATTTTCTCCTTACGCATCTGTGCGGTATTTCACACCGCAATGGTGCAC
TCTCAGTACAATCTGCTCTGATGCCGCATAGTTAAGCCAGTATACACTCCGCTATCGCTACGTGACTGGGTCATGGCTGCGCCCCGACACCCGCCAACACCCGCTGA
CGCGCCCTGACGGGCTTGTCTGCTCCCGGCATCCGCTTACAGACAAGCTGTGACCGTCTCCGGGAGCTGCATGTGTCAGAGGTTTTCACCGTCATCACCGAAACGCG
CGAGGCAGCTGCGGTAAAGCTCATCAGCGTGGTCGTGAAGCGATTCACAGATGTCTGCCTGTTCATCCGCGTCCAGCTCGTTGAGTTTCTCCAGAAGCGTTAATGTC
TGGCTTCTGATAAAGCGGGCCATGTTAAGGGCGGTTTTTTCCTGTTTGGTCACTGATGCCTCCGTGTAAGGGGGATTTCTGTTCATGGGGGTAATGATACCGATGAA
ACGAGAGAGGATGCTCACGATACGGGTTACTGATGATGAACATGCCCGGTTACTGGAACGTTGTGAGGGTAAACAACTGGCGGTATGGATGCGGCGGGACCAGAGAA
AAATCACTCAGGGTCAATGCCAGCGCTTCGTTAATACAGATGTAGGTGTTCCACAGGGTAGCCAGCAGCATCCTGCGATGCAGATCCGGAACATAATGGTGCAGGGC
GCTGACTTCCGCGTTTCCAGACTTTACGAAACACGGAAACCGAAGACCATTCATGTTGTTGCTCAGGTCGCAGACGTTTTGCAGCAGCAGTCGCTTCACGTTCGCTC
GCGTATCGGTGATTCATTCTGCTAACCAGTAAGGCAACCCCGCCAGCCTAGCCGGGTCCTCAACGACAGGAGCACGATCATGCGCACCCGTGGGGCCGCCATGCCGG
CGATAATGGCCTGCTTCTCGCCGAAACGTTTGGTGGCGGGACCAGTGACGAAGGCTTGAGCGAGGGCGTGCAAGATTCCGAATACCGCAAGCGACAGGCCGATCATC
GTCGCGCTCCAGCGAAAGCGGTCCTCGCCGAAAATGACCCAGAGCGCTGCCGGCACCTGTCCTACGAGTTGCATGATAAAGAAGACAGTCATAAGTGCGGCGACGAT
AGTCATGCCCCGCGCCCACCGGAAGGAGCTGACTGGGTTGAAGGCTCTCAAGGGCATCGGTCGAGATCCCGGTGCCTAATGAGTGAGCTAACTTACATTAATTGCGT
TGCGCTCACTGCCCGCTTTCCAGTCGGGAAACCTGTCGTGCCAGCTGCATTAATGAATCGGCCAACGCGCGGGGAGAGGCGGTTTGCGTATTGGGCGCCAGGGTGGT
TTTTCTTTTCACCAGTGAGACGGGCAACAGCTGATTGCCCTTCACCGCCTGGCCCTGAGAGAGTTGCAGCAAGCGGTCCACGCTGGTTTGCCCCAGCAGGCGAAAAT
CCTGTTTGATGGTGGTTAACGGCGGGATATAACATGAGCTGTCTTCGGTATCGTCGTATCCCACTACCGAGATATCCGCACCAACGCGCAGCCCGGACTCGGTAATG
GCGCGCATTGCGCCCAGCGCCATCTGATCGTTGGCAACCAGCATCGCAGTGGGAACGATGCCCTCATTCAGCATTTGCATGGTTTGTTGAAAACCGGACATGGCACT
CCAGTCGCCTTCCCGTTCCGCTATCGGCTGAATTTGATTGCGAGTGAGATATTTATGCCAGCCAGCCAGACGCAGACGCGCCGAGACAGAACTTAATGGG

His Tag
Avi Tag
hPADI4

hPADI4_C645A

```
GTTTCGGTGATGACGGTGAAAACCTCTGACACATGCAGCTCCCGGAGACGGTCACAGCTTGTCTGTAAGCGGATGCCGGGAGCAGACAAGCCCGTCAGGGCGCGTCA
GCGGGTGTTGGCGGGTGTCGGGGCGCAGCCATGACCCAGTCACGTAGCGATAGCGGAGTGTATACTGGCTTAACTATGCGGCATCAGAGCAGATTGTACTGAGAGTG
CACCATATGCGGTGTGAAATACCGCACAGATGCGTAAGGAGAAAATACCGCATCAGGCGCTCTTCCGCTTCCTCGCTCACTGACTCGCTGCGCTCGGTCGTTCGGCT
GCGGCGAGCGGTATCAGCTCACTCAAAGGCGGTAATACGGTTATCCACAGAATCAGGGGATAACGCAGGAAAGAACATGTGAGCAAAAGGCCAGCAAAAGGCCAGGA
ACCGTAAAAAGGCCGCGTTGCTGGCGTTTTTCCATAGGCTCCGCCCCCCTGACGAGCATCACAAAAATCGACGCTCAAGTCAGAGGTGGCGAAACCCGACAGGACTA
TAAAGATACCAGGCGTTTCCCCCTGGAAGCTCCCTCGTGCGCTCTCCTGTTCCGACCCTGCCGCTTACCGGATACCTGTCCGCCTTTCTCCCTTCGGGAAGCGTGGC
GCTTTCTCATAGCTCACGCTGTAGGTATCTCAGTTCGGTGTAGGTCGTTCGCTCCAAGCTGGGCTGTGTGCACGAACCCCCCGTTCAGCCCGACCGCTGCGCCTTAT
CCGGTAACTATCGTCTTGAGTCCAACCCGGTAAGACACGACTTATCGCCACTGGCAGCAGCCACTGGTAACAGGATTAGCAGAGCGAGGTATGTAGGCGGTGCTACA
GAGTTCTTGAAGTGGTGGCCTAACTACGGCTACACTAGAAGGACAGTATTTGGTATCTGCGCTCTGCTGAAGCCAGTTACCTTCGGAAAAAGAGTTGGTAGCTCTTG
ATCCGGCAAACAAACCACCGCTGGTAGCGGTGGTTTTTTTGTTTGCAAGCAGCAGATTACGCGCAGAAAAAAAGGATCTCAAGAAGATCCTTTGATCTTTTCTACGG
GGTCTGACGCTCAGTGGAACGAAAACTCACGTTAAGGGATTTTGGTCATGAGATTATCAAAAAGGATCTTCACCTAGATCCTTTTAAATTAAAAATGAAGTTTTAAA
TCAATCTAAAGTATATATGAGTAAACTTGGTCTGACAGTTACCAATGCTTAATCAGTGAGGCACCTATCTCAGCGATCTGTCTATTTCGTTCATCCATAGTTGCCTG
ACTCCCCGTCGTGTAGATAACTACGATACGGGAGGGCTTACCATCTGGCCCCAGTGCTGCAATGATACCGCGAGACCCACGCTCACCGGCTCCAGATTTATCAGCAA
TAAACCAGCCAGCCGGAAGGGCCGAGCGCAGAAGTGGTCCTGCAACTTTATCCGCCTCCATCCAGTCTATTAATTGTTGCCGGGAAGCTAGAGTAAGTAGTTCGCCA
GTTAATAGTTTGCGCAACGTTGTTGCCATTGCTACAGGCATCGTGGTGTCACGCTCGTCGTTTGGTATGGCTTCATTCAGCTCCGGTTCCCAACGATCAAGGCGAGT
TACATGATCCCCCATGTTGTGCAAAAAAGCGGTTAGCTCCTTCGGTCCTCCGATCGTTGTCAGAAGTAAGTTGGCCGCAGTGTTATCACTCATGGTTATGGCAGCAC
TGCATAATTCTCTTACTGTCATGCCATCCGTAAGATGCTTTTCTGTGACTGGTGAGTACTCAACCAAGTCATTCTGAGAATAGTGTATGCGGCGACCGAGTTGCTCT
TGCCCGGCGTCAATACGGGATAATACCGCGCCACATAGCAGAACTTTAAAAGTGCTCATCATTGGAAAACGTTCTTCGGGGCGAAAACTCTCAAGGATCTTACCGCT
GTTGAGATCCAGTTCGATGTAACCCACTCGTGCACCCAACTGATCTTCAGCATCTTTTACTTTCACCAGCGTTTCTGGGTGAGCAAAAACAGGAAGGCAAAATGCCG
CAAAAAAGGGAATAAGGGCGACACGGAAATGTTGAATACTCATACTCTTCCTTTTTCAATATTATTGAAGCATTTATCAGGGTTATTGTCTCATGAGCGGATACATA
TTTGAATGTATTTAGAAAAATAAACAAATAGGGGTTCCGCGCACATTTCCCCGAAAAGTGCCACCTGACGTCTAAGAAACCATTATTATCATGACATTAACCTATAA
AAATAGGCGTATCACGAGGCCCTTTCGTCTTCACCTCGATTCGTCTTCACCTCGAGAAATCATAAAAAATTTATTTGCTTTGTGAGCGGATAACAATTATAATAGAT
TCAATTGTGAGCGGATAACAATTTCACACAGAATTTATTAAAGAGGAGAAATTACATATGTCCGGCCTGAACGACATCTTCGAGGCTCAGAAAATCGAATGGCACGA
AGGTCACCATCATCACCACCACGGATCCATGGCCCAGGGGACATTGATCCGTGTGACCCCAGAGCAGCCCACCCATGCCGTGTGTGTGCTGGGCACCTTGACTCAGC
TTGACATCTGCAGCTCTGCCCCTGAGGACTGCACGTCCTTCAGCATCAACGCCTCCCCAGGGGTGGTCGTGGATATTGCCCACAGCCCTCCAGCCAAGAAGAAATCC
ACAGGTTCCTCCACATGGCCCCTGGACCCTGGGGTAGAGGTGACCCTGACGATGAAAGCGGCCAGTGGTAGCACAGGCGACCAGAAGGTTCAGATTTCATACTACGG
ACCCAAGACTCCACCAGTCAAAGCTCTACTCTACCTCACCGCGGTGGAAATCTCCCTGTGCGCAGACATCACCCGCACCGGCAAAGTGAAGCCAACCAGAGCTGTGA
AAGATCAGAGGACCTGGACCTGGGGCCCTTGTGGACAGGGTGCCATCCTGCTGGTGAACTGTGACAGAGACAATCTCGAATCTTCTGCCATGGACTGCGAGGATGAT
GAAGTGCTTGACAGCGAAGACCTGCAGGACATGTCGCTGATGACCCTGAGCACGAAGACCCCCAAGGACTTCTTCACAAACCATACACTGGTGCTCCACGTGGCCAG
GTCTGAGATGGACAAAGTGAGGGTGTTTCAGGCCACACGGGGCAAACTGTCCTCCAAGTGCAGCGTAGTCTTGGGTCCCAAGTGGCCCTCTCACTACCTGATGGTCC
CCGGTGGAAAGCACAACATGGACTTCTACGTGGAGGCCCTCGCTTTCCCGGACACCGACTTCCCGGGGCTCATTACCCTCACCATCTCCCTGCTGGACACGTCCAAC
CTGGAGCTCCCCGAGGCTGTGGTGTTCCAAGACAGCGTGGTCTTCCGCGTGGCGCCCTGGATCATGACCCCCAACACCCAGCCCCCGCAGGAGGTGTACGCGTGCAG
TATTTTTGAAAATGAGGACTTCCTGAAGTCAGTGACTACTCTGGCCATGAAAGCCAAGTGCAAGCTGACCATCTGCCCTGAGGAGGAGAACATGGATGACCAGTGGA
TGCAGGATGAAATGGAGATCGGCTACATCCAAGCCCCACACAAAACGCTGCCCGTGGTCTTCGACTCTCCAAGGAACAGAGGCCTGAAGGAGTTTCCCATCAAACGA
GTGATGGGTCCAGATTTTGGCTATGTAACTCGAGGGCCCCAAACAGGGGGTATCAGTGGACTGGACTCCTTTGGGAACCTGGAAGTGAGCCCCCCAGTCACAGTCAG
GGGCAAGGAATACCCGCTGGGCAGGATTCTCTTCGGGGACAGCTGTTATCCCAGCAATGACAGCCGGCAGATGCACCAGGCCCTACAGGACTTCCTCAGTGCCCAGC
AGGTGCAGGCCCCTGTGAAGCTCTATTCTGACTGGCTGTCCGTGGGCCACGTGGACGAGTTCCTGAGCTTTGTGCCAGCACCCGACAGGAAGGGCTTCCGGCTGCTC
CTGGCCAGCCCCAGGTCCTGCTACAAACTGTTCCAGGAGCAGCAGAATGAGGGCCACGGGGAGGCCCTGCTGTTCGAAGGGATCAAGAAAAAAAAACAGCAGAAAAT
AAAGAACATTCTGTCAAACAAGACATTGAGAGAACATAATTCATTTGTGGAGAGATGCATCGACTGGAACCGCGAGCTGCTGAAGCGGGAGCTGGGCCTGGCCGAGA
GTGACATCATTGACATCCCGCAGCTCTTCAAGCTCAAAGAGTTCTCTAAGGCGGAAGCTTTTTTCCCCAACATGGTGAACATGCTGGTGCTAGGGAAGCACCTGGGC
ATCCCCAAGCCCTTCGGGCCCGTCATCAACGGCCGCTGCTGCCTGGAGGAGAAGGTGTGTTCCCTGCTGGAGCCACTGGGCCTCCAGTGCACCTTCATCAACGACTT
CTTCACCTACCACATCAGGCATGGGGAGGTGCACGCCGGCACCAACGTGCGCAGAAAGCCCTTCTCCTTCAAGTGGTGGAACATGGTGCCCTAAAATTCAGGGTACC
CAATAAGCTTACAATTGGTGGTACATAGAATGGTTAACTTTAACAAGGAGAAAAACATGAAGGATAACACCGTGCCACTGAAATTGATTGCCCTGTTAGCGAACGGT
GAATTTCACTCTGGCGAGCAGTTGGGTGAAACGCTGGGAATGAGCCGGGCGGCTATTAATAAACACATTCAGACACTGCGTGACTGGGGCGTTGATGTCTTTACCGT
TCCGGGTAAAGGATACAGCCTGCCTGAGCCCATCCAGTTACTTAATGCTGAACAGATATTGGGTCAGCTGGATGGCGGTAGTGTAGCCGTGCTGCCAGTTATTGACT
CCACGAATCAGTACCTTCTTGATCGTATCGGAGAGCTTAAATCGGGCGATGCCTGTGTTGCAGAATACCAGCAGGCTGGCCGTGGTCGCCGGGGGCGGAAATGGTTT
TCGCCTTTTGGCGCAAACTTATATTTGTCGATGTTCTGGCGTCTGGAACAAGGCCCGGCGGCGGCGATTGGTTTAAGTCTGGTTATCGGTATCGTGATGGCGGAAGT
ATTACGCAAGCTGGGAGCAGATAAAGTTCGTGTCAAATGGCCTAATGACCTCTATCTGCAGGATCGCAAGCTGGCAGGCATTCTTGTGGAGCTGACTGGCAAAACTG
GCGATGCGGCGCAAATAGTCATTGGAGCCGGGATCAACATGGCAATGCGCCGTGTTGAAGAGAGTGTCGTTAATCAGGGGTGGATCACGCTGCAGGAAGCGGGGATC
AATCTCGATCGTAATACGTTGGCGGCCATGCTAATACGTGAATTACGTGCTGCGTTGGAACTCTTCGAACAAGAAGGATTGGCACCTTATCTGTCGCGCTGGGAAAA
GCTGGATAATTTTATTAATCGCCCAGTGAAACTTATCATTGGTGATAAAGAAATATTTGGCATTTCACGCGGAATAGACAAACAGGGGGCTTTATTACTTGAGCAGG
ATGGAATAATAAAACCCTGGATGGGCGGTGAAATATCCCTGCGTAGTGCAGAAAAATAAGCGGCCGCAGCTTAATTAGCTGAGCTTGGACTCCTGTTGATAGATCCA
GTAATGACCTCAGAACTCCATCTGGATTTGTTCAGAACGCTCGGTTGCCGCCGGGCGTTTTTTATTGGTGAGAATCCAAGCTAGCTTGGCGAGATTTTCAGGAGCTA
AGGAAGCTAAAATGGAGAAAAAAATCACTGGATATACCACCGTTGATATATCCCAATGGCATCGTAAAGAACATTTTGAGGCATTTCAGTCAGTTGCTCAATGTACC
TATAACCAGACCGTTCAGCTGGATATTACGGCCTTTTTAAAGACCGTAAAGAAAAATAAGCACAAGTTTTATCCGGCCTTTATTCACATTCTTGCCCGCCTGATGAA
TGCTCATCCGGAATTTCGTATGGCAATGAAAGACGGTGAGCTGGTGATATGGGATAGTGTTCACCCTTGTTACACCGTTTTCCATGAGCAAACTGAAACGTTTTCAT
CGCTCTGGAGTGAATACCACGACGATTTCCGGCAGTTTCTACACATATATTCGCAAGATGTGGCGTGTTACGGTGAAAACCTGGCCTATTTCCCTAAAGGGTTTATT
GAGAATATGTTTTTCGTCTCAGCCAATCCCTGGGTGAGTTTCACCAGTTTTGATTTAAACGTGGCCAATATGGACAACTTCTTCGCCCCCGTTTTCACCATGGGCAA
ATATTATACGCAAGGCGACAAGGTGCTGATGCCGCTGGCGATTCAGGTTCATCATGCCGTTTGTGATGGCTTCCATGTCGGCAGAATGCTTAATGAATTACAACAGT
ACTGCGATGAGTGGCAGGGCGGGGCGTAATTTTTTTAAGGCAGTTATTGGTGCCCTTAAACGCCTGGGGTAATGACTCTCTAGCTTGAGGCATCAAATAAAACGAAA
GGCTCAGTCGAAAGACTGGGCCTTTCGTTTTATCTGTTGTTTGTCGGTGAACGCTCTCCTGAGTAGGACAAATCCGCCCTCTAGATTACGTGCAGTCGATGATAAGC
TGTCAAACATGAGAATTGTGCCTAATGAGTGAGCTAACTTACATTAATTGCGTTGCGCTCACTGCCCGCTTTCCAGTCGGGAAACCTGTCGTGCCAGCTGCATTAAT
GAATCGGCCAACGCGCGGGGAGAGGCGGTTTGCGTATTGGGCGCCAGGGTGGTTTTTCTTTTCACCAGTGAGACGGGCAACAGCTGATTGCCCTTCACCGCCTGGCC
CTGAGAGAGTTGCAGCAAGCGGTCCACGCTGGTTTGCCCCAGCAGGCGAAAATCCTGTTTGATGGTGGTTAACGGCGGGATATAACATGAGCTGTCTTCGGTATCGT
CGTATCCCACTACCGAGATATCCGCACCAACGCGCAGCCCGGACTCGGTAATGGCGCGCATTGCGCCCAGCGCCATCTGATCGTTGGCAACCAGCATCGCAGTGGGA
ACGATGCCCTCATTCAGCATTTGCATGGTTTGTTGAAAACCGGACATGGCACTCCAGTCGCCTTCCCGTTCCGCTATCGGCTGAATTTGATTGCGAGTGAGATATTT
ATGCCAGCCAGCCAGACGCAGACGCGCCGAGACAGAACTTAATGGGCCCGCTAACAGCGCGATTTGCTGGTGACCCAATGCGACCAGATGCTCCACGCCCAGTCGCG
TACCGTCTTCATGGGAGAAAATAATACTGTTGATGGGTGTCTGGTCAGAGACATCAAGAAATAACGCCGGAACATTAGTGCAGGCAGCTTCCACAGCAATGGCATCC
TGGTCATCCAGCGGATAGTTAATGATCAGCCCACTGACGCGTTGCGCGAGAAGATTGTGCACCGCCGCTTTACAGGCTTCGACGCCGCTTCGTTCTACCATCATCAC
CACCACGCTGGCACCCAGTTGATCGGCGCGAGATTTAATCGCCGCGACAATTTGCGACGGCGCGTGCAGGGCCAGACTGGAGGTGGCAACGCCAATCAGCAACGACT
GTTTGCCCGCCAGTTGTTGTGCCACGCGGTTGGGAATGTAATTCAGCTCCGCCATCGCCGCTTCCACTTTTTCCCGCGTTTTCGCAGAAACGTGGCTGGCCTGGTTC
ACCACGCGGGAAACGGTCTGATAAGAGACACCGGCATACTCTGCGACATCGTATAACGTTACTGGTTTCACATTCACCACCCTGAATTGACTCTCTTCCGGGCGCTA
TCATGCCATACCGCGAAAGGTTTTGCACCATTCGATGGTGTCGGAATTTCGGGCAGCGTTGGGTCCTGGCCACGGGTGCGCATGATCTAGAGCTGCCTCGCGC
```

His Tag
Avi Tag
hPADI4_C645A
