# Supplementary material for: Discovering covalent cyclic peptide inhibitors of peptidyl arginine deiminase 4 (PADI4) using mRNA-display with a genetically encoded electrophilic warhead
Source: Commun Chem. 2024 Dec 19;7:304. doi: 10.1038/s42004-024-01388-9 (PMC11659602; doi:10.1038/s42004-024-01388-9)
Supplement: Supplementary file 3 — Description of Additional Supplementary Files [file 42004_2024_1388_MOESM3_ESM.pdf]

Description of Additional Supplementary Files

File name: Supplementary Data 1

Description: Round 1 to 5 sequencing data for selection against PADI4, converted into peptide sequences.

File name: Supplementary Data 2

Description: Round 2 to 6 sequencing data for selection against PADI4, converted into peptide sequences.

File name: Supplementary Data 3

Description: Round 1 to 6 sequencing data for selection against PADI4, converted into peptide sequences.

File name: Supplementary Data 4

Description: NMR spectra

File name: Supplementary Data 5

Description: Source Data
