# Supplementary material for: Discovering covalent cyclic peptide inhibitors of peptidyl arginine deiminase 4 (PADI4) using mRNA-display with a genetically encoded electrophilic warhead
Source: Commun Chem. 2024 Dec 19;7:304. doi: 10.1038/s42004-024-01388-9 (PMC11659602; doi:10.1038/s42004-024-01388-9)

**Supplementary Data 4- NMR Spectra.**

Isabel R. Mathiesen,1,2 Ewen D. D. Calder,1,2 Simone Kunzelmann,^3^ Louise J. Walport1,2*

1 Protein-Protein Interaction Laboratory, The Francis Crick Institute, London NW1 1AT, United Kingdom

2 Department of Chemistry, Molecular Sciences Research Hub, Imperial College London, London W12 0BZ, United Kingdom

^3^Structural Biology Scientific Technology Platform, The Francis Crick Institute, London NW1 1AT, United Kingdom

*To whom correspondence should be addressed: [l.walport@imperial.ac.uk](mailto:l.walport@imperial.ac.uk)

^1^H NMR (400 MHz, CD_3_OD) - Ethyl 2-fluoroethanimidate hydrochloride (**S1**).

^19^F NMR (376 MHz, CD_3_OD) - Ethyl 2-fluoroethanimidate hydrochloride (**S1**).


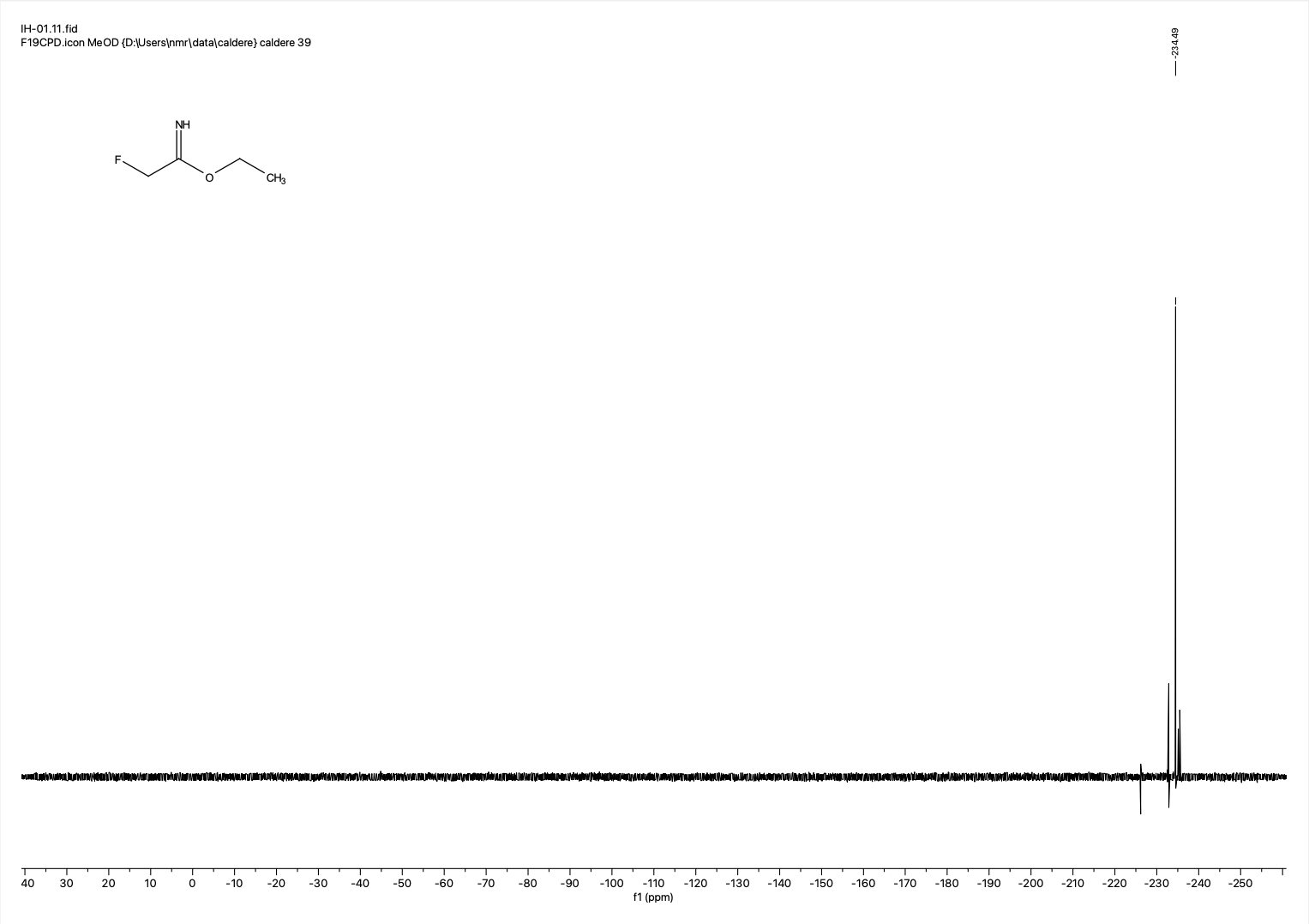


^1^H NMR (400 MHz, DMSO-*d_6_*) - 2,2,2-trichloroethyl acetimidate hydrochloride (**S2**).

^1^H NMR (400 MHz, CD_3_OD) – Ethyl 2-chloroethanimidate hydrochloride (**S3**).

^1^H NMR (400 MHz, CD_3_OD) - *N*-α-Boc-*N*-δ-(2- fluoroacetimidoyl)-ornithine (**S4**).

^13^C NMR (101 MHz, DMSO-*d*_6_) - *N*-α-Boc-*N*-δ-(2- fluoroacetimidoyl)-ornithine (**S4**).


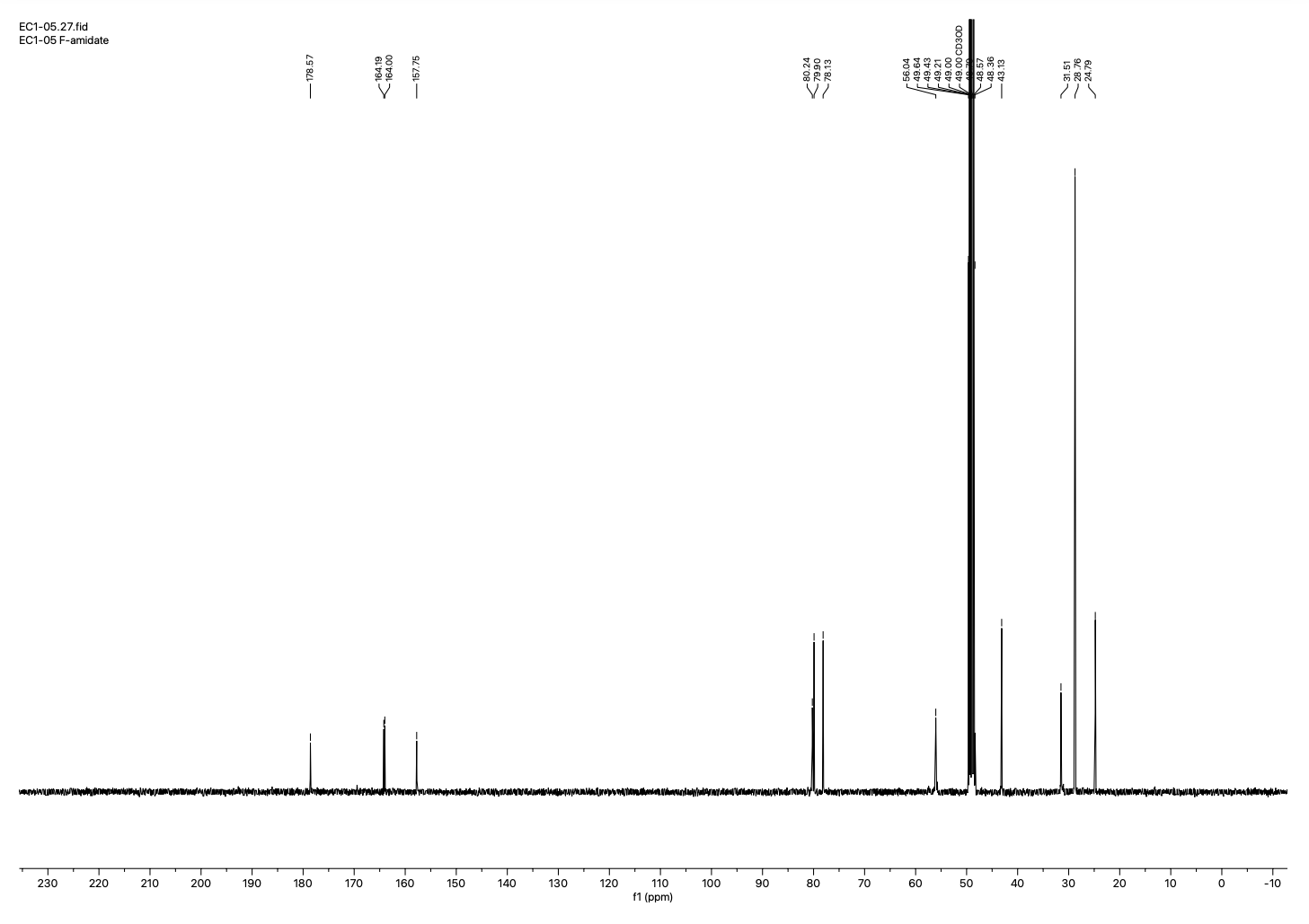


^19^F NMR (376 MHz, CDCl_3_) - *N*-α-Boc-*N*-δ-(2- fluoroacetimidoyl)-ornithine (**S4**).


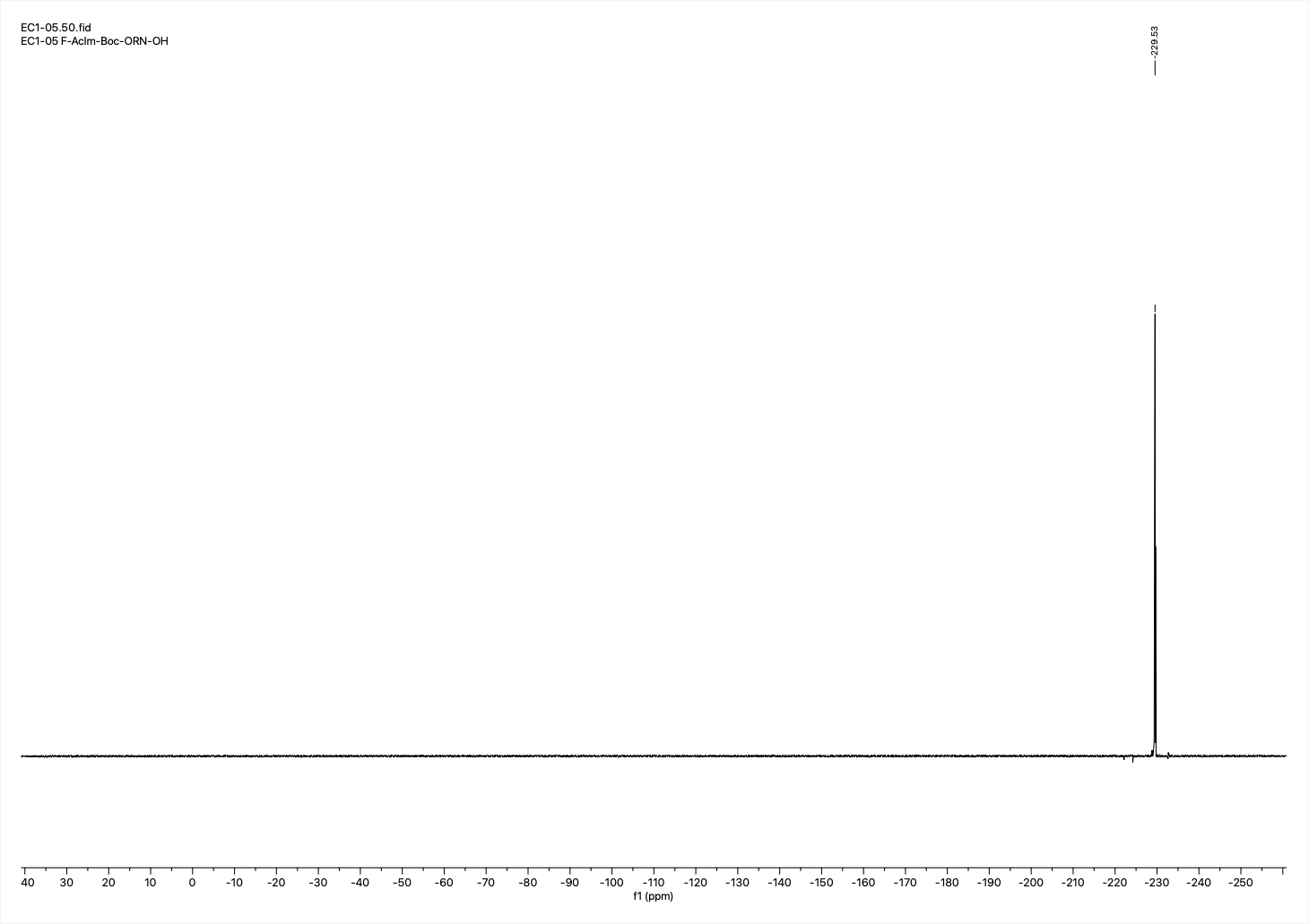


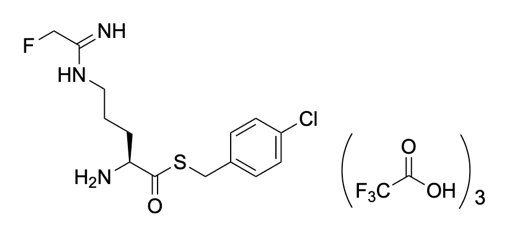
^1^H NMR (400 MHz, DMSO-*d*_6_) - *N*-δ-(2-fluoroacetimidoyl)-ornithine chlorobenzylthioester FAO-CBT (**2**).

^13^C NMR (101 MHz, DMSO-*d*_6_) - *N*-δ-(2-fluoroacetimidoyl)-ornithine chlorobenzylthioester FAO-CBT (**2**).


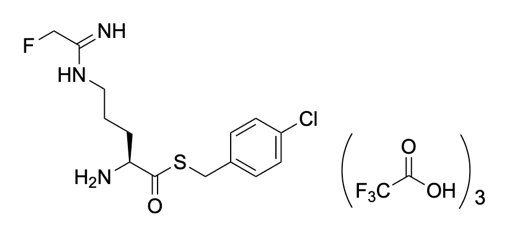


^19^F NMR (376 MHz, CDCl_3_) - *N*-δ-(2-fluoroacetimidoyl)-ornithine chlorobenzylthioester FAO-CBT (**2**).


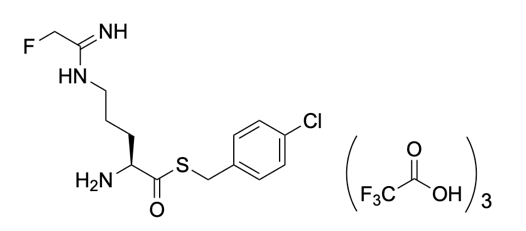

Supplement: Supplementary file 7 — Supplementary Data 4 [file 42004_2024_1388_MOESM7_ESM.docx]
